# Iodine as an efficient initiator for the transfer-hydrogenation from 1,4-cyclohexadiene to aryl-substituted alkenes and the deoxygenation of benzylic alcohols

**DOI:** 10.1039/d5ra07048a

**Published:** 2025-11-27

**Authors:** Sascha Kail, Gerhard Hilt

**Affiliations:** a Institute of Chemistry, Carl von Ossietzky University Oldenburg Carl-von-Ossietzky-Str. 9-11 26129 Oldenburg Germany Gerhard.Hilt@uni-oldenburg.de

## Abstract

The transfer-hydrogenation emerged as an interesting alternative to traditional hydrogenations with H_2_ under transition metal catalysis. Herein, we describe I_2_ as an initiator for the catalysis in CH_2_Cl_2_ for the transfer-hydrogenation from 1,4-cyclohexadiene to aryl-substituted alkenes as well as for the deoxygenation of benzylic alcohols at room temperature.

## Abstract 2

The transfer-hydrogenation from cyclohexadiene to aryl-substituted alkenes as well as the deoxygenation of benzylic alcohols in dichloromethane is realised with a catalytic amount of iodine at ambient temperatures.

## Graphical abstract

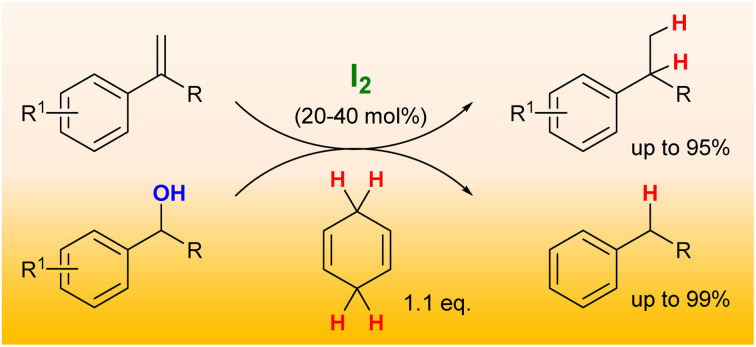

Historically, there is a one-reaction-publication by Eberhardt in 1967 reporting the transfer-hydrogenation from CHD to styrene utilising a I_2_/CHD system to generate ethylbenzene.^1^ However a broader investigation on the scope and limitations of this very simple and easy applicable system is missing in the literature. The transfer-hydrogenation from dihydroaromatic compounds to carbon–carbon double bonds was investigated in great detail by Oestreich in utilising boron-based Lewis acids, such as B(C_6_F_5_)_3_, as catalysts (Scheme 1).^2–6^ Thereafter several reports appeared with different Lewis acids, such as InBr_3_,^7^ [IPrGaCl_2_][SbF_6_]^8^ or alkaline-earth metal catalysts^9^ which led to milder reaction conditions and an increased substrate scope.

**Scheme 1:**
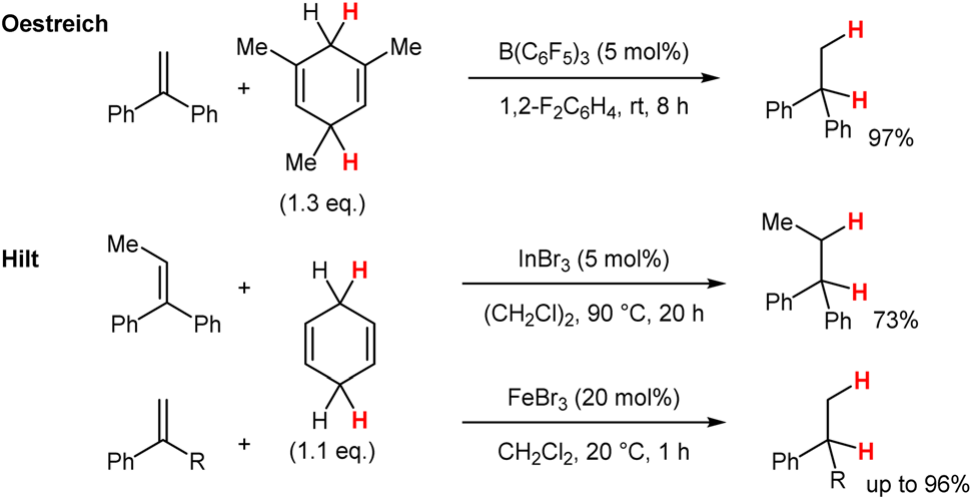
Transfer-hydrogenations with Lewis acid catalysts.

Eventually, in 2024 we reported the FeBr_3_ catalysed transfer-hydrogenation with 1,4-cyclohexadiene (CHD) which could be performed at ambient temperature.^10^ Meanwhile the regioselective H-D addition to alkenes^11^ as well as an asymmetric version for the transfer-hydrogenation to alkenes were also reported.^12^

In a recent report, Xu and Liu disclosed the transfer-hydrogenation utilizing cyclic ketones, such as cycloheptanone or cyclododecanone, as the hydrogen donor for the hydrogenation of 1,1-diarylethene derivatives (Scheme 2).^13^ Unfortunately, high reaction temperatures and sometimes prolonged reaction times are needed to accomplish this highly interesting transformation.

**Scheme 2:**
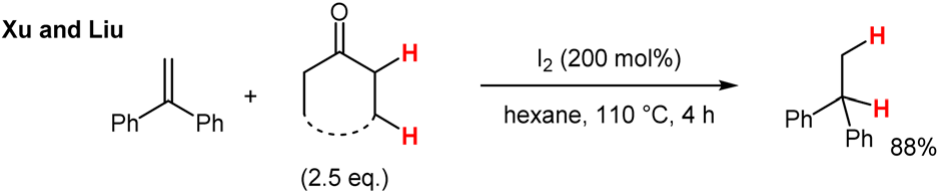
Transfer-hydrogenation from ketones to alkenes with I_2_ as the “catalyst”.

However, the fact that the α-position of ketones can be easily transformed into α-perdeuterated ketones and these derivatives were applied in the regioselective H-D transfer to the 1,1-diarylethenes is of considerable interest.

Based on these latest reports, and the initial report by Eberhardt,^1^ we became interested in expanding the application of I_2_ as the catalyst (or initiator) for the transfer-hydrogenation from CHD as H_2_-surrogate to a broader range of alkenes. For this purpose, we selected 1,1-diphenylethene 1a as a substrate for the conversion with CHD (1.1 eq.) under I_2_ catalysis for the optimisation of the reaction conditions (Table 1).

**Table 1:** Optimisation of the transfer-hydrogenation from CHD to alkenes with I_2_ as the catalyst/initiator under a N_2_ atmosphere

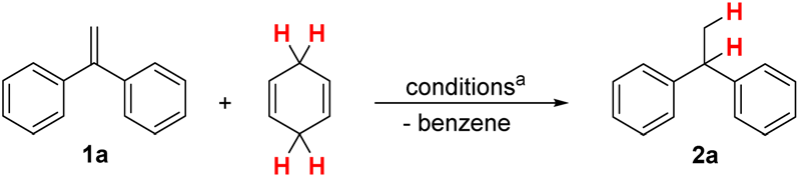				
Entry	I_2_ loading (mol%)	Solvent	Yield^b^ (after 15 min)	Yield^b^ (after 1 h)
1	5	CH_2_Cl_2_	42%	59%
2	12	CH_2_Cl_2_	55%	68%
3	15	CH_2_Cl_2_	67%	78%
**4**	**20**	**CH**_**2**_ **Cl**_**2**_	**95%**	**95%**
5	20	Hexane	95%	95%
6	20	CHCl_3_	56%	73%
7	20	THF	0%	0%
8	20	MeCN	0%	0%
9	20	DMF	0%	0%
10	20	DMSO	0%	0%

a Reaction conditions: 1a (0.5 mmol), CHD (0.55 mmol), solvent (0.5 mL), N_2_ atm., rt.

b Determined by GC analysis of the crude mixture with mesitylene as internal standard, added from a stock solution after the reaction.

The optimisation of the transfer-hydrogenation from CHD to aryl-substituted alkenes, such as 1a, at ambient temperatures was initiated by test reactions in dichloromethane utilising increasing amounts of I_2_ (Table 1, entries 1–4). The best results were obtained when 20 mol% of I_2_ were employed which led to 95% yield determined by GC analysis after 15 minutes reaction time (isolated yield of 2a: 95%; see Scheme 3). Similar results were obtained for hexane as solvent (entry 5) which gave 95% yield after 15 minutes as well. Other solvents, such as chloroform (entry 6) were inferior and more polar solvents (entries 7–10) prohibited the desired transformation completely.

**Scheme 3:**
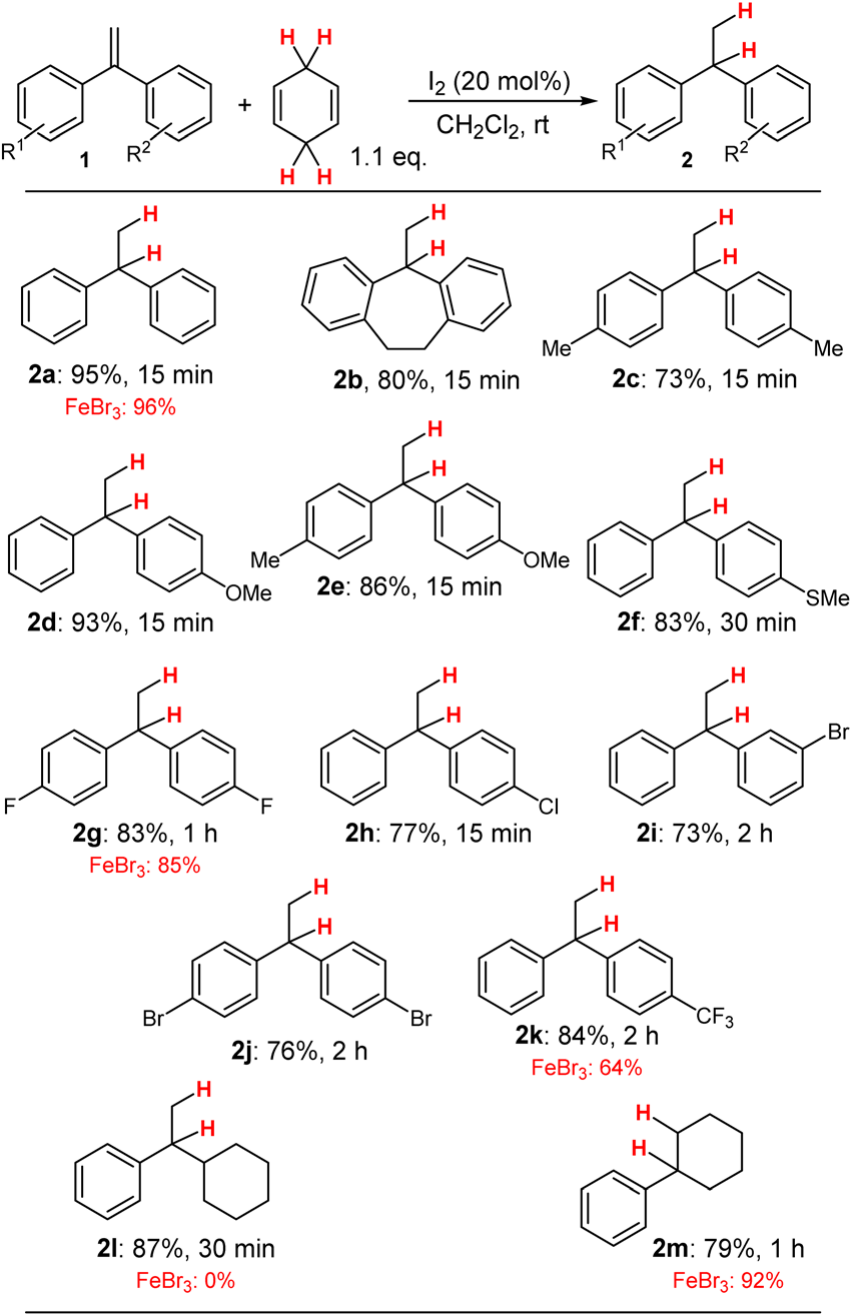
Scope of the transfer-hydrogenation from CHD to aryl-substituted utilising I_2_ as catalyst under N_2_ atmosphere. Reaction conditions: 1 (0.5 mmol), CHD (0.55 mmol), CH_2_Cl_2_ (0.5 mL), N_2_ atm., rt.

With the optimised reaction conditions for the transformation of 1a in hand, we then investigated other substrates of type 1 for the transfer-hydrogenation from CHD to these aryl-substituted alkenes (Scheme 3). For this set of experiments, we applied 20 mol% of I_2_ in dichloromethane as solvent at ambient temperature. Further substituents (R^1^/R^2^) were incorporated to determine the electronic effects rather than steric effects on the transfer-hydrogenation. The results for these transformations are given together with our previous results utilising FeBr_3_ as Lewis acid catalyst when available for comparison.^10^

The basic system 1a gave the desired product 2a in a very similar yield as the previously described FeBr_3_ catalyst system. But since I_2_ is less sensitive to water compared to anhydrous FeBr_3_, this is a significant advantage over the FeBr_3_ system. Then simple alkyl-substituted diaryl alkenes (1b and 1c) were tested and, in these transformations, the products 2b and 2c could be isolated in good yields after 15 minutes reaction time. When methoxy groups were incorporated in the substrates, such as in 2d and 2e, the reaction led to high conversions, as well as with a thioether substituent in 2f affording product 2f in 83% yield, although the reaction time had to be slightly expanded to 30 minutes.

Then, we tested additional halide substituents on the phenyl rings to determine their influence on the reaction performance. The 4,4′-difluoro derivative 2g was obtained in similar yields compared to the FeBr_3_ system which resulted in similar results after 1 h reaction time. The conversion of the 4-chloro derivative 1h for the formation of 2h, the 3-bromo derivative to generate product 2i as well as the 4,4′-dibromo derivative 2j were successful and the desired products could be isolated in overall good yields. Surprisingly, when a substrate with a strong electron-withdrawing substituent in the *para*-position of the arene moiety was investigated, herein the CF_3_-substituted substrate 1k, which destabilize the proposed carbenium ion intermediate (see below), the desired product 2k could be isolated in a good yield for of 84%, which is superior to the FeBr_3_/CHD catalysed system.

For this type of transformation, where the stability of a transition carbenium ions is essential, the less stabilizing arene substituents are encountered, the less efficient the hydrogen-transfer reaction will be performed.

Therefore, we became increasingly interested, whether I_2_/CHD or the FeBr_3_/CHD system would make any difference. Compared to the FeBr_3_/CHD system, the I_2_/CHD system performed very efficient for the synthesis of product 2l but a little less effective for the synthesis of 2m, which was isolated in 79% yield compared to 92% when the reaction was catalysed by FeBr_3_.

Nevertheless, the I_2_/CHD system exemplified itself as an easily applicable system which is highly cost-efficient and of low harm and is an excellent method for the transfer-hydrogenation from CHD to aryl-substituted alkenes. Therefore, we also became interested in similar transformations which were realised with I_2_ as a reagent or catalyst to realise reductions.^14,15^

Under these preconditions, we came across I_2_-promoted transformations for the deoxygenation of alcohols and ketones. In this context, in the past there were several remarkable reports from the Fry group regarding the deoxygenation of 1,1-diarylketones^16^ and the deoxygenation of 1,1-diarylmethanol derivatives with I_2_ and over-stoichiometric amounts of H_3_PO_2_ (Scheme 4).^17–19^ Also remarkable are reports for the reductive deoxygenation utilising silanes, such as polymethylsiloxane (PMHS) as reducing agents under iodide catalysis (HI)^20^ and the divergent transformations of benzylic alcohols under I_2_ catalysis.^21^

**Scheme 4:**
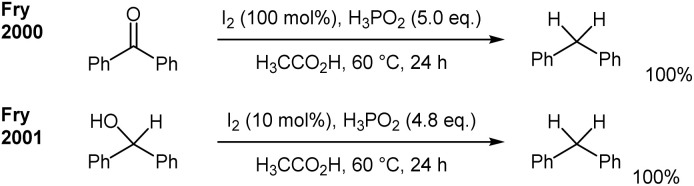
Deoxygenation of benzylic ketones and benzylic alcohols with I_2_/H_3_PO_2_ in acetic acid under reflux conditions.

For the deoxygenation of benzylic alcohols, we choose diphenylmethanol 3a as the test substrate and tested different amounts of I_2_ in a small number of solvents under ambient temperatures. The results of this optimisation are summarised in Table 2.

**Table 2:** Optimisation of the deoxygenation with I_2_ and CHD

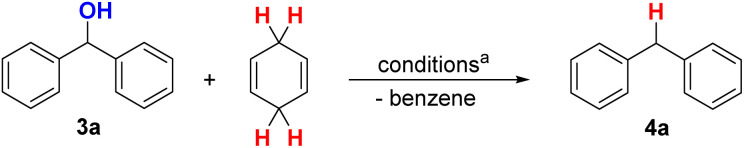							
Entry	I_2_ loading (mol%)	Solvent	Yield^b^				
			1 h	2 h	3 h	4 h	20 h
1	10	CH_2_Cl_2_	5%	6%	7%	9%	15%
2	20	CH_2_Cl_2_	16%	26%	37%	48%	68%
3	30	CH_2_Cl_2_	21%	38%	50%	75%	86%
**4**	**40**	**CH**_**2**_ **Cl**_**2**_	**57%**	**85%**	**98%**	**98%**	**98%**
5	40	Hexane	54%	83%	98%	98%	98%
6	20	CHCl_3_	6%	12%	18%	25%	39%
7	20	THF	0%	0%	0%	0%	0%

a Reaction conditions: 3a (0.5 mmol), CHD (0.55 mmol), solvent (0.5 mL), N_2_ atm., rt.

b Determined by GC analysis of the crude mixture with mesitylene as internal standard, added from a stock solution after the reaction.

Compared to the previous disclosed transfer-hydrogenation from CHD to aryl-substituted alkenes, the deoxygenation needed higher I_2_ loadings and extended reaction times. However, when 40 mol% of I_2_ were applied, the desired product 4a was detected by GC analysis in almost quantitative yield (98%) after 3 h reaction time (entry 4).

Again, dichloromethane and hexane (entries 4/5) exhibited similar performance, while chloroform (entry 6) proved to be inferior again and tetrahydrofuran (THF; entry 7) is not suited for this transformation.

The other polar solvents investigated in Table 1 were not tested for this transformation, as dichloromethane and hexane proved to be very well suited for this deoxygenation reaction.

With these reaction conditions in hand, we then tested several carbinol derivatives of type 3 to generate the deoxygenated products of type 4 under mild reaction conditions. The results of these transformations are summarised in Scheme 5.

**Scheme 5:**
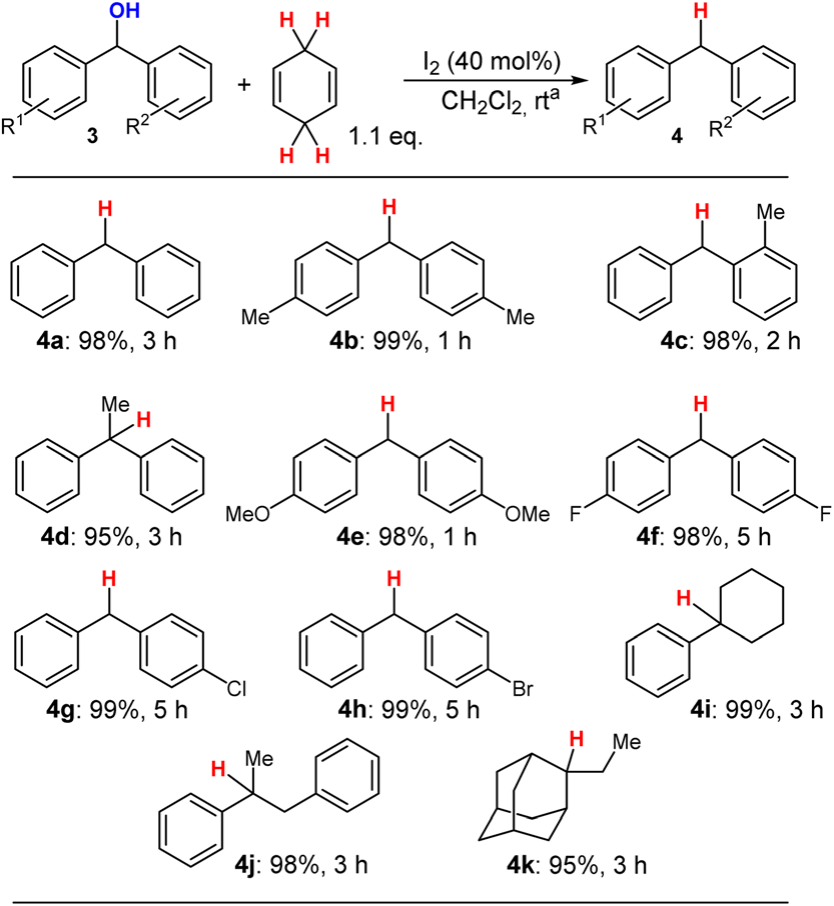
Scope of the I_2_-promoted deoxygenation of alcohols with CHD. ^*a*^Reaction conditions: 3 (0.5 mmol), CHD (0.55 mmol), CH_2_Cl_2_ (0.5 mL), N_2_ atm., rt.

In general, the applied carbinol-type substrates 4a–4h were deoxygenated in very high yields (>95%) and only the reaction time had to be adjusted for each substrate, ranging from 1 to 5 hours. We then tested also aryl-alkyl substituted benzylic alcohols and the products 4i and 4j could be obtained in very high yields (>98%) as well. Also, the adamantly derivative 3k was converted into 4k in an excellent yield of 95% which shows that this transformation has the potential be applicable also for non-benzylic alcohols in the future.

Finally, we also tested a cyclopropyl-substituted starting materials to prove that the reaction mechanism is of ionic character and not following radical mechanisms (see Schemes 6/7). For this purpose, substrates 1n and 3l were used and in contrast to the previous reactions investigated thus far, the following observations were made:

**Scheme 6:**
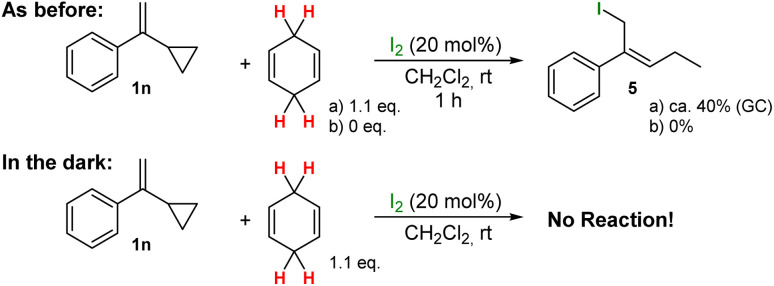
Reactions with cyclopropyl-substituted substrate 1n under the optimised conditions, in the absence of CHD and in the dark.

**Scheme 7:**
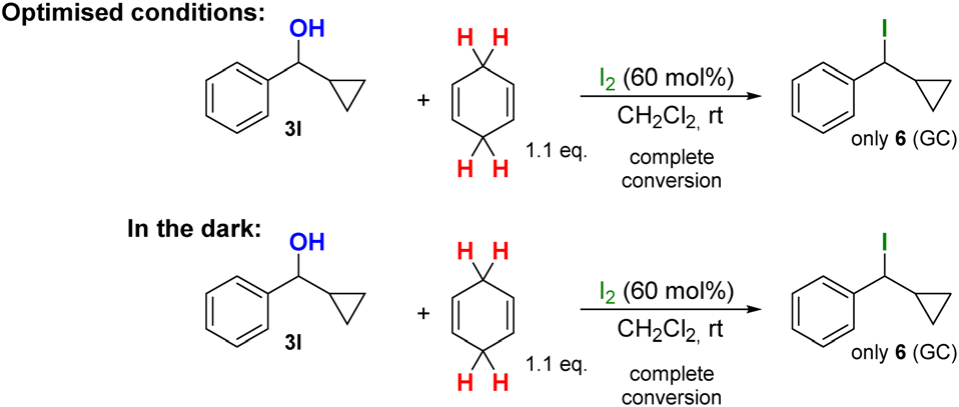
Reactions with cyclopropyl-substituted substrate 3l under the optimised conditions and in the dark with 60 mol% I_2_.

For substrate 1n:

• The hydrogenation of the cyclopropyl-substituted alkene 1n gave the product 5 with iodine incorporated in the product. This incorporation was only observed for this transformation!

• In the absence of CHD no reaction was observed.

• When the reactions of 1n was performed in the dark, no reaction was observed.

For substrate 3l:

• The brown-coloured reaction solution only decolorised with substrate 3l. In all other reactions, the coloured solution persisted.

• Only in product 6, the incorporation of iodine in the product was observed as determined by GCMS and NMR analysis of the crude product.

• When the reactions for the cyclopropyl-substituted alcohol 3l was performed in the dark, in the presence of CHD complete conversion to 6 was observed.

• In several reactions, a bis-carbinol ether intermediate, such as Ph_2_CH–O–CHPh_2_, was detected by GCMS analysis, indicating that the reaction is most likely following an ionic mechanism for those substrates. When the reaction of 3l was performed in the dark in the absence of CHD, only the formation of the corresponding two diastereomeric dimers could be observed.

In general, the mechanistic investigations of reactions with cyclopropyl-substituted substrates are a well-accepted method to differentiate between ionic and radical reaction mechanisms. However, we believe that in this special case, the reaction mechanism is for whatever reason, altered when a cyclopropyl substituent alkene or an alcohol are involved, thus leading to completely different products (=5 and 6) and different chemical behaviour when the reactions are performed in the absence of light or in the absence of CHD. For substrate 1n, light and CHD as H˙ donor seem to be essential for the radical-type ring opening of 1n to 5. In contrast, educt 3l reacts with and without light efficiently to give 6 as single product. Whereas in the absence of CHD, only the formation of the corresponding ether is observed, indicating that this reaction is following an ionic mechanism.

Accordingly, we believe that the transformations outlined in this report are of ionic character. The I_2_ is oxidizing CHD to form benzene and HI in small amounts at first.^22^ The HI initiates the proton catalysis and is reacting with the starting materials of type 1 (and 3 – not shown) leading to stabilized carbenium ions of type A (Scheme 8).

**Scheme 8:**
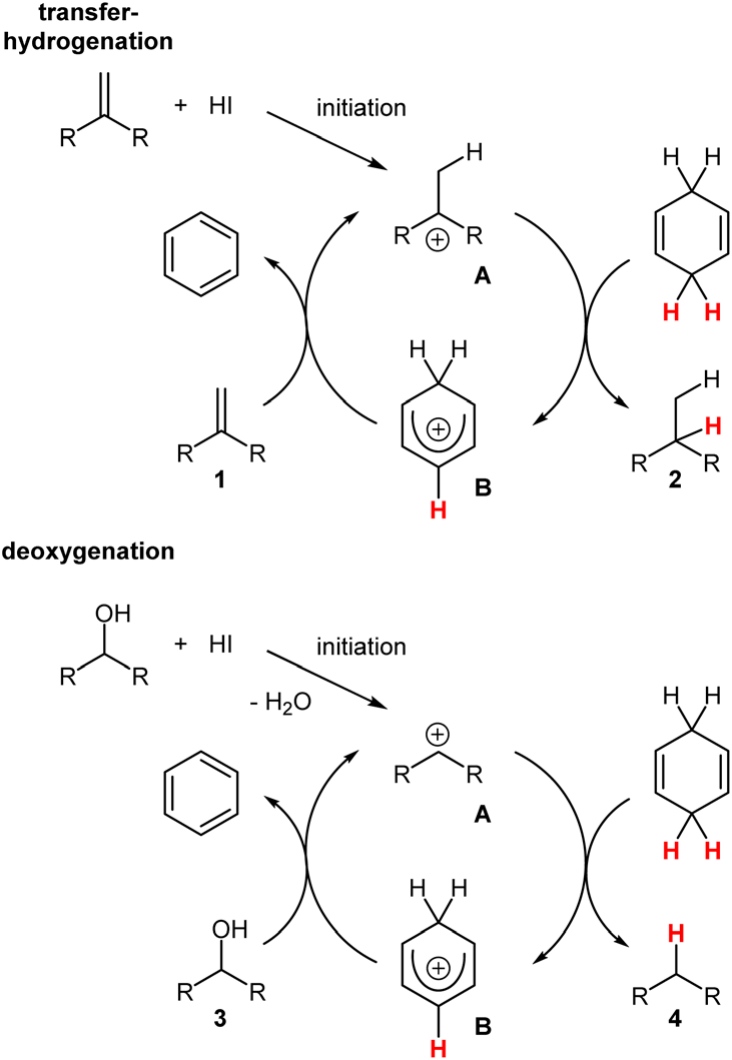
Proposed mechanism of the transfer-hydrogenation and the deoxygenation with the I_2_/CHD system.

The intermediate A then abstracts a hydride ion from CHD to form the desired products 2 and simultaneously generates the arenium ion B (= Wheland complex), which acts as a strong Brönstedt acid, to protonate the starting materials 1 and thereby closes the catalytic cycle. For the starting materials of type 3, water is formed as a good leaving group, but the deoxygenation reaction also follows the same principle. However, in these reactions radical mechanisms cannot be excluded.

In summary, we have developed an I_2_-initiated transfer-hydrogenation of aryl-substituted alkenes under very mild conditions utilising CHD as a H_2_-surrogate. Also, the I_2_-initiated deoxygenation of benzylic alcohols was investigated which led to excellent results when the reaction time is adjusted to the substrate. In addition, this transformation could be conducted ambient reaction conditions and opens the way for further investigations with the I_2_/CHD system for more sophisticated deoxygenations that we have in mind.

## Conflicts of interest

There are no conflicts to declare.

## Supplementary Material

RA-015-D5RA07048A-s001

## Data Availability

The data supporting this article have been included as part of the supplementary information (SI). Supplementary information is available. See DOI: https://doi.org/10.1039/d5ra07048a.
