# Supplementary material for: Iodine as an efficient initiator for the transfer-hydrogenation from 1,4-cyclohexadiene to aryl-substituted alkenes and the deoxygenation of benzylic alcohols
Source: RSC Adv. 2025 Nov 27;15(55):46838–42. doi: 10.1039/d5ra07048a (PMC12658735; doi:10.1039/d5ra07048a)
Supplement: RA-015-D5RA07048A-s001 [file RA-015-D5RA07048A-s001.pdf]

This journal is © The Royal Society of Chemistry 2025

Table of Content

1. General Information 2
2. Iodine-catalysed Transfer-Hydrogenation 3
2.1 Synthesised Products 3
3. Iodine-catalysed Deoxygenation 10
3.1 Synthesised Products 10
4. Control experiments with cyclopropane derivatives 15
5. NMR spectra of all synthesised products 18
5. References 41

1. General Information

All solvents were commercially available and have been distilled under reduced pressure prior to use. Solvents were dried over 3 Å molecular sieves. All chemicals or reagents were purchased from commercial suppliers without further purification, if not otherwise stated, or were prepared according to known literature procedures. If water or air sensitive compounds have been used, the experiments were carried out in oven-dried glassware using conventional SCHLENK techniques under nitrogen atmosphere. All known compounds were characterized by ^{1}H and ^{13}C NMR and ^{19}F NMR if applicable. Unknown compounds were identified by ^{1}H NMR, ^{13}C NMR, ^{19}F NMR if applicable, IR and HRMS.

NMR spectroscopy: NMR spectra were recorded either on a Bruker Avance 300 (300 MHz), on a Bruker Avance III (500 MHz) or on a Bruker Avance DRX (500 MHz). Chemical shifts are reported in parts per million (ppm). The spectra are referenced to the residual solvent peak of $CDCl_3$ or CD_2Cl_2. In the ^{1}H NMR spectra this corresponds with the singlet of the solvent signal of $CDCl_3$ at δ = 7.26 ppm and CD_2Cl_2 at δ = 5.32 ppm. The ^{13}C NMR spectra were referenced to the central line of the triplet of $CDCl_3$ at δ = 77.16 ppm or of the quintet of CD_2Cl_2 at δ = 53.84 ppm. The stated form of the signal describes the appearance of the signal and not the theoretically expected form.

Chromatography: Flash chromatography was carried out using Macherey-Nagel silica gel 60 (0.040-0.063 mm). Thin layer chromatography was carried out on Merck TLC plates coated with silica gel 60 F_{254} with fluorescence indicator. For the detection of the signals ultraviolet light (λ = 254 nm) or GC analysis were used or heating after the plate has been dipped into a $KMnO_4$ solution.

2. Iodine-catalysed Transfer-Hydrogenation

General Procedure 1:

An oven dried sealed tube was flushed with nitrogen and charged with a stir bar and 1 mL DCM. Then the corresponding alkene (1 mmol, 1.0 equiv.), 1,4-cyclohexadiene (104 µL, 1.1 mmol, 1.1 equiv.) and iodine (50.8 mg, 0.2 mmol, 20 mol%) were added in sequence. The mixture was stirred at 20°C and the progress of the reaction was monitored by GC-MS. After complete conversion, a saturated sodium thiosulphate solution (10 mL) was added to the reaction mixture and the aqueous phase was extracted with DCM (10 mL). The combined organic phase was dried over magnesium sulfate and filtered. The solvent was removed under reduced pressure and the crude product was purified by flash column chromatography.

2.1 Synthesised Products

Ethane-1,1-diyldibenzene (2a)

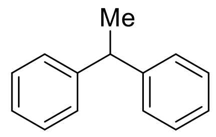

According to the general procedure 1 the title compound was prepared using ethene-1,1-diyldibenzene (180 mg, 1.0 mmol, 1.0 equiv.) at 20°C in DCM (15 min). The product was obtained as a colourless liquid (173 mg, 0.95 mmol, 95%).

R*f* (SiO_2, *n*-pentane) = 0.33.

¹H-NMR (500 MHz, $CDCl_3$): δ = 7.36-7.31 (m, 4H), 7.30-7.27 (m, 4H), 7.25-7.21 (m, 2H), 4.21 (q, *J* = 7.2 Hz, 1H), 1.70 (d, *J* = 7.2 Hz, 3H) ppm.

¹³C-NMR (125 MHz, $CDCl_3$): δ = 146.5, 128.5, 127.8, 126.1, 44.9, 22.0 ppm.

The analytical data are in accordance with the literature.[1]

5-Methyl-10,11-dihydro-5H-dibenzo[a,d][7]annulene (2b)

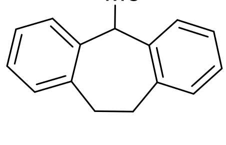

According to the general procedure 1 the title compound was prepared using 5-methylene-10,11-dihydro-5H-dibenzo[a,d]cycloheptene (206 mg, 1.0 mmol, 1.0 equiv.) at 20°C in DCM (15 min). The product was obtained as a colourless oil (166 mg, 0.80 mmol, 80%).

R_f (SiO_2, *n*-pentane) = 0.26.

^{1}H-NMR (500 MHz, $CDCl_3$): δ = 7.28-7.23 (m, 2H), 7.19-7.10 (m, 6H), 4.46 (q, *J* = 7.4 Hz, 1H), 3.30-3.19 (m, 4H), 1.75 (d, *J* = 7.4 Hz, 3H) ppm.

^{13}C-NMR (125 MHz, $CDCl_3$): δ = 143.3, 139.4, 130.2, 127.5, 126.4, 126.3, 43.8, 33.3, 22.1 ppm.

The analytical data are in accordance with the literature.[2]

1,1-Di(4-methylphenyl)ethane (2c)

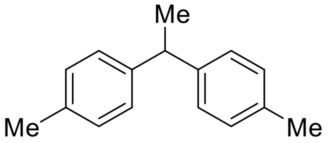

According to the general procedure 1 the title compound was prepared using 1,1-di(p-tolyl)ethylene (208 mg, 1.0 mmol, 1.0 equiv.) at 20°C in DCM (15 min). The product was obtained as a colourless oil (154 mg, 0.73 mmol, 73%).

R_f (SiO_2, *n*-pentane) = 0.35.

^{1}H-NMR (500 MHz, $CDCl_3$): δ = 7.19-7.12 (m, 8H), 4.14 (q, *J* = 7.2 Hz, 1H), 2.36 (s, 6H), 1.67 (d, *J* = 7.2 Hz, 3H) ppm.

^{13}C-NMR (125 MHz, $CDCl_3$): δ = 143.8, 135.5, 129.2, 127.6, 44.1, 22.1, 21.1 ppm.

The analytical data are in accordance with the literature.[2]

1-Methoxy-4-(1-phenylethyl)benzene (2d)

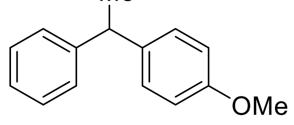

According to the general procedure 1 the title compound was prepared using 1-(4-methoxyphenyl)-1-phenylethene (210 mg, 1.0 mmol, 1.0 equiv.) at 20°C in DCM (15 min). The product was obtained as a colourless oil (198 mg, 0.93 mmol, 93%).

R_f (SiO_2, *n*-pentane/Et_2O, 20:1) = 0.78.

^{1}H-NMR (500 MHz, $CDCl_3$): δ = 7.34-7.29 (m, 2H), 7.27-7.16 (m, 5H), 6.89-6.85 (m, 2H), 4.15 (q, *J* = 7.2 Hz, 1H), 3.81 (s, 3H), 1.66 (d, *J* = 7.2 Hz, 3H) ppm.

^{13}C-NMR (125 MHz, $CDCl_3$): δ = 158.0, 146.9, 138.7, 128.6, 128.5, 127.7, 126.1, 113.8, 55.3, 44.1, 22.2 ppm.

The analytical data are in accordance with the literature.[3]

1-Methoxy-4-(1-(p-tolyl)ethyl)benzene (2e)

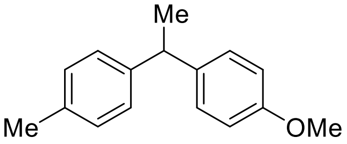

According to the general procedure 1 the title compound was prepared using 1-methoxy-4-(1-(p-tolyl)vinyl)benzene (224 mg, 1.0 mmol, 1.0 equiv.) at 20°C in DCM (15 min). The product was obtained as a colourless oil (195 mg, 0.86 mmol, 86%).

R_f (SiO_2, *n*-pentane/Et_2O, 20:1) = 0.70.

^{1}H-NMR (500 MHz, $CDCl_3$): δ = 7.20-7.11 (m, 6H), 6.89-6.84 (m, 4H), 4.11 (q, *J* = 7.2 Hz, 1H), 3.81 (s, 3H), 2.35 (s, 3H), 1.64 (d, *J* = 7.2 Hz, 3H) ppm.

^{13}C-NMR (125 MHz, $CDCl_3$): δ = 157.9, 143.9, 138.9, 135.5, 129.2, 128.6, 127.5, 113.8, 55.3, 43.6, 22.3, 21.1 ppm.

The analytical data are in accordance with the literature.[3]

Methyl(4-(1-phenylethyl)phenyl)sulfane (2f)

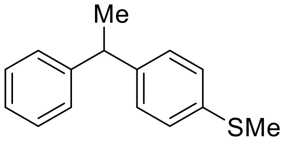

According to the general procedure 1 the title compound was prepared using methyl(4-(1-phenylvinyl)phenyl)sulfane (226 mg, 1.0 mmol, 1.0 equiv.) at 20°C in DCM (30 min). The product was obtained as a pale yellow oil (190 mg, 0.83 mmol, 83%).

R_f (SiO_2, *n*-pentane/Et_2O, 100:1) = 0.48.

^{1}H-NMR (500 MHz, $CDCl_3$): δ = 7.34-7.28 (m, 2H), 7.25-7.15 (m, 7H), 4.14 (q, *J* = 7.2 Hz, 1H), 2.48 (s, 3H), 1.65 (d, *J* = 7.2 Hz, 3H) ppm.

^{13}C-NMR (125 MHz, $CDCl_3$): δ = 146.3, 143.6, 135.7, 128.5, 128.3, 127.7, 127.1, 126.2, 44.4, 21.9, 16.3 ppm.

The analytical data are in accordance with the literature.[3]

4,4'-(Ethane-1,1-diyl)bis(fluorobenzene) (2g)

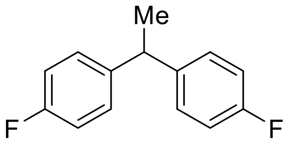

According to the general procedure 1 the title compound was prepared using 1,1-bis(4-fluorophenyl)ethene (216 mg, 1.0 mmol, 1.0 equiv.) at 20°C in DCM (1 h). The product was obtained as a colourless oil (181 mg, 0.83 mmol, 83%).

R*f*(SiO$_2$, *n*-pentane) = 0.50.

^{1}H-NMR (500 MHz, CDCl$_3$): δ = 7.21-7.14 (m, 4H), 7.04-6.97 (m, 4H), 4.15 (q, *J* = 7.2 Hz, 1H), 1.63 (t, *J* = 7.3 Hz, 3H) ppm.

^{13}C-NMR (125 MHz, CDCl$_3$): δ = 162.5 (d, *J* = 224.1 Hz), 142.0 (d, *J* = 2.8 Hz), 129.1 (d, *J* = 7.9 Hz), 115.3 (d, *J* = 21.2 Hz), 43.6, 22.3 ppm.

^{19}F-NMR (470 MHz, CDCl$_3$): δ = -117.16 ppm.

The analytical data are in accordance with the literature.[1]

1-Chloro-4-(1-phenylethyl)benzene (2h)

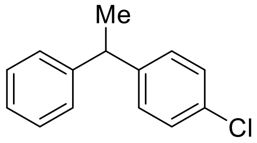

According to the general procedure 1 the title compound was prepared using 1-(4-chlorophenyl)-1-phenylethene (215 mg, 1.0 mmol, 1.0 equiv.) at 20°C in DCM (15 min). The product was obtained as a colourless oil (166 mg, 0.77 mmol, 77%).

R*f* (SiO$_2$, *n*-pentane) = 0.45.

^{1}H-NMR (500 MHz, CDCl$_3$): δ = 7.34-7.30 (m, 2H), 7.29-7.25 (m, 2H), 7.24-7.20 (m, 3H), 7.19-7.15 (m, 2H), 4.15 (q, *J* = 7.2 Hz, 1H), 1.65 (d, *J* = 7.3 Hz, 3H) ppm.

^{13}C-NMR (125 MHz, CDCl$_3$): δ = 145.9, 145.0, 131.9, 129.1, 128.6, 127.7, 126.4, 44.3, 21.9 ppm.

The analytical data are in accordance with the literature.[2]

1-Bromo-3-(1-phenylethyl)benzene (2i)

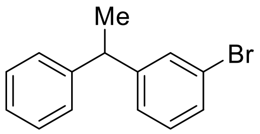

According to the general procedure 1 the title compound was prepared using 1-bromo-3-(1-phenylvinyl)benzene (259 mg, 1.0 mmol, 1.0 equiv.) at 20°C in DCM (2 h). The product was obtained as a colourless oil (191 mg, 0.73 mmol, 73%).

$\mathbf{R}_f$ (SiO_2, *n*-pentane) = 0.48.

^{1}H-NMR (500 MHz, $CDCl_3$): δ = 7.41-7.38 (m, 1H), 7.36-7.29 (m, 3H), 7.25-7.20 (m, 3H), 7.19-7.14 (m, 2H), 4.14 (q, *J* = 7.2 Hz, 1H), 1.64 (d, *J* = 7.3 Hz, 3H) ppm.

^{13}C-NMR (125 MHz, $CDCl_3$): δ = 148.9, 145.6, 130.8, 130.1, 129.3, 128.6, 127.7, 126.5, 126.5, 122.7, 44.7, 21.8 ppm.

The analytical data are in accordance with the literature.[4]

4,4'-(Ethane-1,1-diyl)bis(1-bromobenzene) (2j)

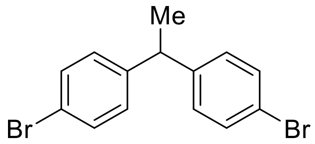

According to the general procedure 1 the title compound was prepared using 1,1-bis(p-bromophenyl)ethylene (338 mg, 1.0 mmol, 1.0 equiv.) at 20°C in DCM (2 h). The product was obtained as a colourless solid (260 mg, 0.76 mmol, 76%).

$\mathbf{R}_f$ (SiO_2, *n*-pentane) = 0.48.

^{1}H-NMR (500 MHz, $CDCl_3$): δ = 7.43-7.38 (m, 4H), 7.09-7.03 (m, 4H), 4.07 (q, *J* = 7.2 Hz, 1H), 1.59 (d, *J* = 7.2 Hz, 3H) ppm.

^{13}C-NMR (125 MHz, $CDCl_3$): δ = 144.9, 131.7, 129.4, 120.2, 43.8, 21.7 ppm.

The analytical data are in accordance with the literature.[2]

1-(1-Phenylethyl)-4-(trifluoromethyl)benzene (2k)

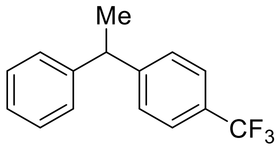

According to the general procedure 1 the title compound was prepared using 1-phenyl-1-(4-trifluoromethylphenyl)ethylene (248 mg, 1.0 mmol, 1.0 equiv.) at 20°C in DCM (2 h). The product was obtained as a colourless liquid (210 mg, 0.84 mmol, 84%).

$\mathbf{R}_f$ (SiO_2, *n*-pentane) = 0.55.

^{1}H-NMR (500 MHz, $CDCl_3$): δ = 7.54 (d, *J* = 8.1 Hz, 2H), 7.35-7.28 (m, 4H), 7.24-7.19 (m, 3H), 4.21 (q, *J* = 7.2 Hz, 1H), 1.67 (t, *J* = 7.2 Hz, 3H) ppm.

^{13}C-NMR (125 MHz, $CDCl_3$): δ = 150.6, 145.4, 128.7, 128.1, 127.7, 126.6, 125.5 (q, *J* = 3.6 Hz), 119.9, 44.9, 21.8 ppm.

^{19}F-NMR (470 MHz, $CDCl_3$): δ = -62.30 ppm.

The analytical data are in accordance with the literature.[1]

(1-Cyclohexylethyl)benzene (2l)

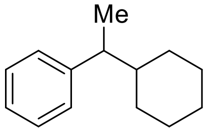

According to the general procedure 1 the title compound was prepared using 1-phenyl-1-cyclohexylethylene (186 mg, 1.0 mmol, 1.0 equiv.) at 20°C for in DCM (30 min). The product was obtained as a colourless liquid (164 mg, 0.87 mmol, 87%).

$\mathbf{R}_f$ (SiO_2, *n*-pentane) = 0.80.

^{1}H-NMR (500 MHz, $CDCl_3$): δ = 7.32-7.27 (m, 2H), 7.22-7.15 (m, 3H) 2.47 (p, *J* = 7.2 Hz, 1H), 1.94-1.88 (m, 1H), 1.81-1.74 (m, 1H), 1.69-1.60 (m, 2H), 1.50-1.37 (m, 2H), 1.26 (d, *J* = 7.1 Hz, 3H), 1.25-1.06 (m, 3H), 1.02-0.91 (m, 1H), 0.88-0.79 (m, 1H) ppm.

^{13}C-NMR (125 MHz, $CDCl_3$): δ = 147.3, 128.2, 127.9, 125.8, 46.1, 44.4, 31.6, 30.8, 26.7, 19.0 ppm.

The analytical data are in accordance with the literature.[5]

1-Phenyl-1-cyclohexane (2m)

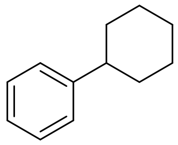

According to the general procedure 1 the title compound was prepared using 1-phenylcyclohexene (158 mg, 1.0 mmol, 1.0 equiv.) at 20°C in DCM (1 h). The product was obtained as a colourless oil (126 mg, 0.79 mmol, 79%).

R*f* (SiO_2, *n*-pentane) = 0.90.

^{1}H-NMR (500 MHz, $CDCl_3$): δ = 7.35-7.30 (m, 2H), 7.27-7.19 (m, 3H), 2.54 (tt, *J* = 11.6, 3.4 Hz, 1H), 1.95-1.85 (m, 4H), 1.80 (dqd, *J* = 12.7, 3.0, 1.5 Hz, 1H), 1.52-1.39 (m, 4H), 1.35-1.27 (m, 1H) ppm.

^{13}C-NMR (125 MHz, $CDCl_3$): δ = 148.2, 128.4, 127.0, 125.9, 44.7, 34.6, 27.1, 26.3 ppm.

The analytical data are in accordance with the literature.[1]

Here are some substrates which could not be converted under the reaction conditions:

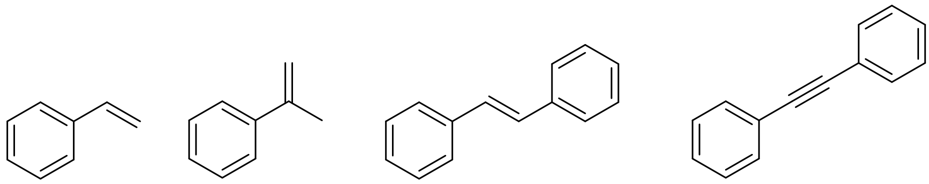

3. Iodine-catalysed Deoxygenation

General Procedure 2:

An oven dried sealed tube was flushed with nitrogen and charged with a stir bar and 1 mL DCM. Then the corresponding alcohol (1 mmol, 1.0 equiv.), 1,4-cyclohexadiene (104 µL, 1.1 mmol, 1.1 equiv.) and iodine (102 mg, 0.4 mmol, 40 mol%) were added in sequence. The mixture was stirred at 20°C and the progress of the reaction was monitored by GC-MS. After complete conversion, a saturated sodium thiosulphate solution (10 mL) was added to the reaction mixture and the aqueous phase was extracted with DCM (10 mL). The combined organic phase was dried over magnesium sulfate and filtered. The solvent was removed under reduced pressure and the product did not need to be further purified.

3.1 Synthesised Products

Diphenylmethane (4a)

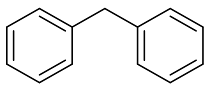

According to the general procedure 2 the title compound was prepared using 1,1-diphenylmethanol (184 mg, 1.0 mmol, 1.0 equiv.) at 20°C in DCM (3 h). The product was obtained as a colourless liquid (165 mg, 0.98 mmol, 98%).

^{1}H-NMR (500 MHz, $CDCl_3$): δ = 7.40-7.34 (m, 4H), 7.32-7.25 (m, 6H), 4.07 (s, 2H) ppm.

^{13}C-NMR (125 MHz, $CDCl_3$): δ = 141.2, 129.1, 128.6, 126.2, 42.1 ppm.

The analytical data are in accordance with the literature.[2]

4,4’-Dimethyldiphenylmethane (4b)

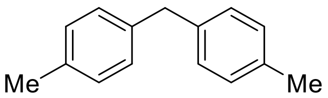

According to the general procedure 2 the title compound was prepared using 4,4’-dimethylbenzhydrol (212 mg, 1.0 mmol, 1.0 equiv.) at 20°C in DCM (1 h). The product was obtained as a colourless liquid (195 mg, 0.99 mmol, 99%).

^{1}H-NMR (500 MHz, $CDCl_3$): δ = 7.16-7.11 (m, 8H), 3.95 (s, 2H), 2.36 (s, 6H) ppm.

^{13}C-NMR (125 MHz, $CDCl_3$): δ = 138.5, 135.5, 129.2, 128.9, 130.1, 128.9, 41.2, 21.1 ppm.

The analytical data are in accordance with the literature.[2]

2-Benzyltoluene (4c)

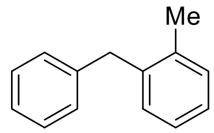

According to the general procedure 2 the title compound was prepared using 2-methylbenzhydrol (198 mg, 1.0 mmol, 1.0 equiv.) at 20°C in DCM (2 h). The product was obtained as a colourless liquid (179 mg, 0.98 mmol, 98%).

^{1}H-NMR (500 MHz, $CDCl_3$): δ = 7.34-7.29 (m, 2H), 7.27-7.10 (m, 7H), 4.04 (s, 2H), 2.30 (s, 3H) ppm.

^{13}C-NMR (125 MHz, $CDCl_3$): δ = 140.5, 139.0, 136.8, 130.4, 130.1, 128.9, 128.5, 126.6, 126.1, 126.0, 39.6, 19.8 ppm.

The analytical data are in accordance with the literature.[6]

Ethane-1,1-diyldibenzene (4d)

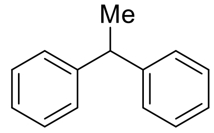

According to the general procedure 2 the title compound was prepared using 1,1-diphenylethanol (198 mg, 1.0 mmol, 1.0 equiv.) at 20°C in DCM (3 h). The product was obtained as a colourless liquid (173 mg, 0.95 mmol, 95%).

^{1}H-NMR (500 MHz, $CDCl_3$): δ = 7.36-7.31 (m, 4H), 7.30-7.27 (m, 4H), 7.25-7.21 (m, 2H), 4.21 (q, J = 7.2 Hz, 1H), 1.70 (d, J = 7.2 Hz, 3H) ppm.

^{13}C-NMR (125 MHz, $CDCl_3$): δ = 146.5, 128.5, 127.8, 126.1, 44.9, 22.0 ppm.

The analytical data are in accordance with the literature.[1]

4,4'-Dianisylmethane (4e)

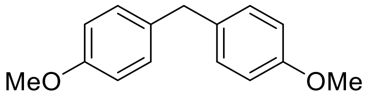

According to the general procedure 2 the title compound was prepared using 4,4'-dimethoxybenzhydrol (244 mg, 1.0 mmol, 1.0 equiv.) at 20°C in DCM (1 h). The product was obtained as a colourless liquid (223 mg, 0.98 mmol, 98%).

^{1}H-NMR (500 MHz, $CDCl_3$): δ = 7.14-7.10 (m, 4H), 6.88-6.84 (m, 4H), 3.89 (s, 2H), 3.80 (s, 6H) ppm.

^{13}**C-NMR** (125 MHz, $CDCl_3$): δ = 158.0, 133.8, 129.8, 113.9, 55.3, 40.2 ppm.

The analytical data are in accordance with the literature.[7]

4,4'-Difluorodiphenylmethane (4f)

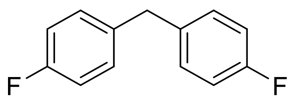

According to the general procedure 2 the title compound was prepared using 4,4'-difluorobenzhydrol (220 mg, 1.0 mmol, 1.0 equiv.) at 20°C in DCM (5 h). The product was obtained as a colourless liquid (201 mg, 0.98 mmol, 98%).

^{1}**H-NMR** (500 MHz, $CDCl_3$): δ = 7.16-7.10 (m, 4H), 7.03-6.96 (m, 4H), 3.94 (s, 2H) ppm.

^{13}**C-NMR** (125 MHz, $CDCl_3$): δ = 161.6 (d, J = 244.3 Hz), 136.7 (d, J = 3.2 Hz), 130.3 (d, J = 7.9 Hz), 115.4 (d, J = 21.2 Hz), 40.4 ppm.

^{19}**F-NMR** (470 MHz, $CDCl_3$): δ = -117.05 ppm.

The analytical data are in accordance with the literature.[2]

4-Chlorophenyl(phenyl)methane (4g)

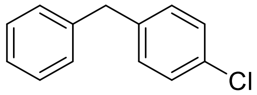

According to the general procedure 2 the title compound was prepared using (4-chlorophenyl)phenylmethanol (219 mg, 1.0 mmol, 1.0 equiv.) at 20°C in DCM (5 h). The product was obtained as a colourless liquid (201 mg, 0.99 mmol, 99%).

^{1}**H-NMR** (500 MHz, $CDCl_3$): δ = 7.34-7.12 (m, 9H), 3.97 (s, 2H) ppm.

^{13}**C-NMR** (125 MHz, $CDCl_3$): δ = 140.7, 139.7, 132.0, 130.4, 129.0, 128.7, 126.4, 41.4 ppm.

The analytical data are in accordance with the literature.[2]

1-Benzyl-4-bromo-benzene (4h)

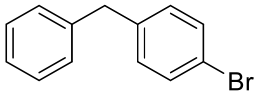

According to the general procedure 2 the title compound was prepared using phenyl(4-bromophenyl)methanol (263 mg, 1.0 mmol, 1.0 equiv.) at 20°C in DCM (5 h). The product was obtained as a colourless liquid (245 mg, 0.99 mmol, 99%).

¹H-NMR (500 MHz, $CDCl_3$): δ = 7.45-7.41 (m, 2H), 7.34-7.29 (m, 2H), 7.27-7.22 (m, 1H), 7.21-7.17 (m, 2H), 7.10-7.06 (m, 2H), 3.95 (s, 2H) ppm.

¹³C-NMR (125 MHz, $CDCl_3$): δ = 140.6, 140.2, 131.6, 130.8, 129.0, 128.7, 126.4, 120.1, 41.4 ppm.

The analytical data are in accordance with the literature.[8]

1-Phenyl-1-cyclohexane (4i)

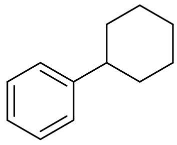

According to the general procedure 2 the title compound was prepared using 1-phenylcyclohexanol (176 mg, 1.0 mmol, 1.0 equiv.) at 20°C in DCM (3 h). The product was obtained as a colourless liquid (159 mg, 0.99 mmol, 99%).

¹H-NMR (500 MHz, $CDCl_3$): δ = 7.35-7.30 (m, 2H), 7.27-7.19 (m, 3H), 2.54 (tt, *J* = 11.6, 3.4 Hz, 1H), 1.95-1.85 (m, 4H), 1.80 (dqd, *J* = 12.7, 3.0, 1.5 Hz, 1H), 1.52-1.39 (m, 4H), 1.35-1.27 (m, 1H) ppm.

¹³C-NMR (125 MHz, $CDCl_3$): δ = 148.2, 128.4, 127.0, 125.9, 44.7, 34.6, 27.1, 26.3 ppm.

The analytical data are in accordance with the literature.[1]

1,2-Diphenylpropane (4j)

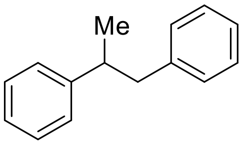

According to the general procedure 2 the title compound was prepared using 1,2-diphenylpropane-2-ol (212 mg, 1.0 mmol, 1.0 equiv.) at 20°C in DCM (3 h). The product was obtained as a colourless oil (193 mg, 0.98 mmol, 98%).

¹H-NMR (500 MHz, $CDCl_3$): δ = 7.41-7.32 (m, 4H), 7.31-7.25 (m, 4H), 7.22-7.17 (m, 2H), 3.15-3.03 (m, 2H), 2.88 (dd, *J* = 13.1, 8.1 Hz, 1H), 1.36 (d, *J* = 6.8 Hz, 3H) ppm.

¹³C-NMR (125 MHz, $CDCl_3$): δ = 147.1, 140.9, 129.3, 128.4, 128.2, 127.2, 126.1, 126.0, 45.2, 42.0, 21.3 ppm.

The analytical data are in accordance with the literature.[1]

2-Ethyladamantane (4k)

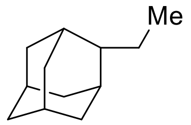

According to the general procedure 2 the title compound was prepared using 2-ethyl-2-adamantol (180 mg, 1.0 mmol, 1.0 equiv.) at 20°C in DCM (3 h). The product was obtained as a colourless liquid (156 mg, 0.95 mmol, 95%).

^{1}H-NMR (500 MHz, $CDCl_3$): δ = 1.89-1.81 (m, 5H), 1.79-1.68 (m, 7H), 1.55-1.40 (m, 5H), 0.88 (t, *J* = 7.3 Hz, 3H) ppm.

^{13}C-NMR (125 MHz, $CDCl_3$): δ = 46.6, 39.5, 38.7, 31.8, 31.7, 28.6, 28.3, 25.5, 12.3 ppm.

The analytical data are in accordance with the literature.[9]

Here are some substrates which could not be converted under the reaction conditions:

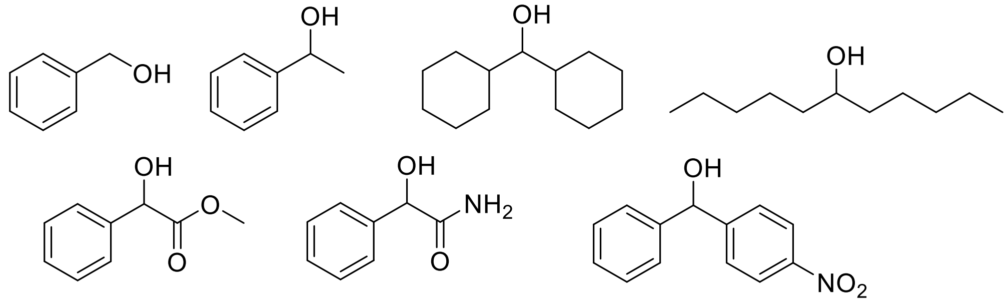

4. Control experiments with cyclopropane derivatives

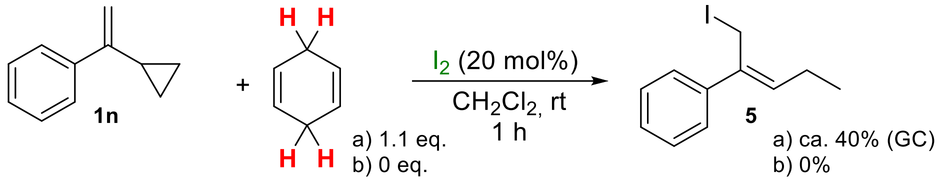

a) GCMS, under the optimised conditions

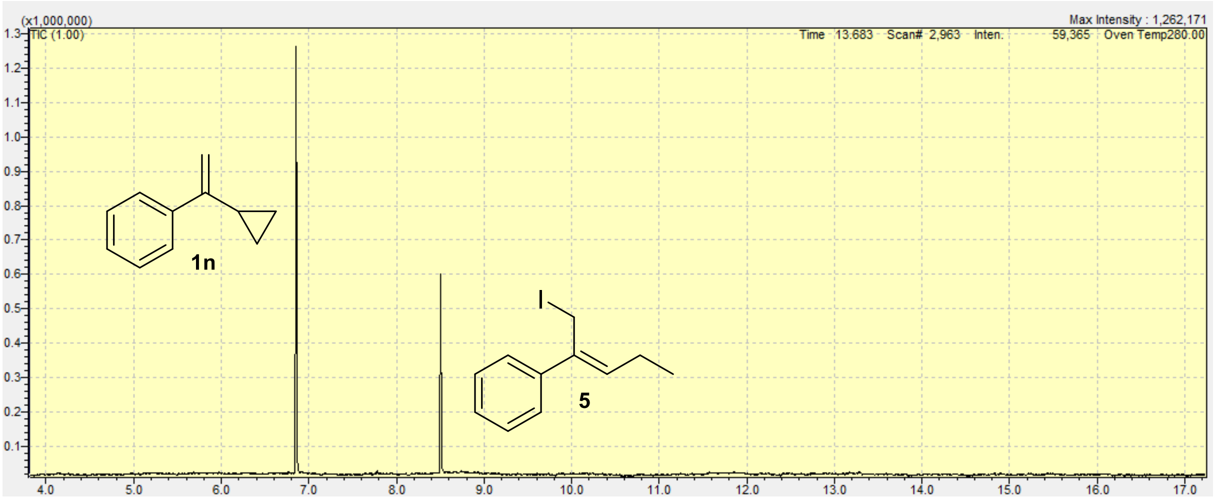

b) GCMS, in the absence of CHD

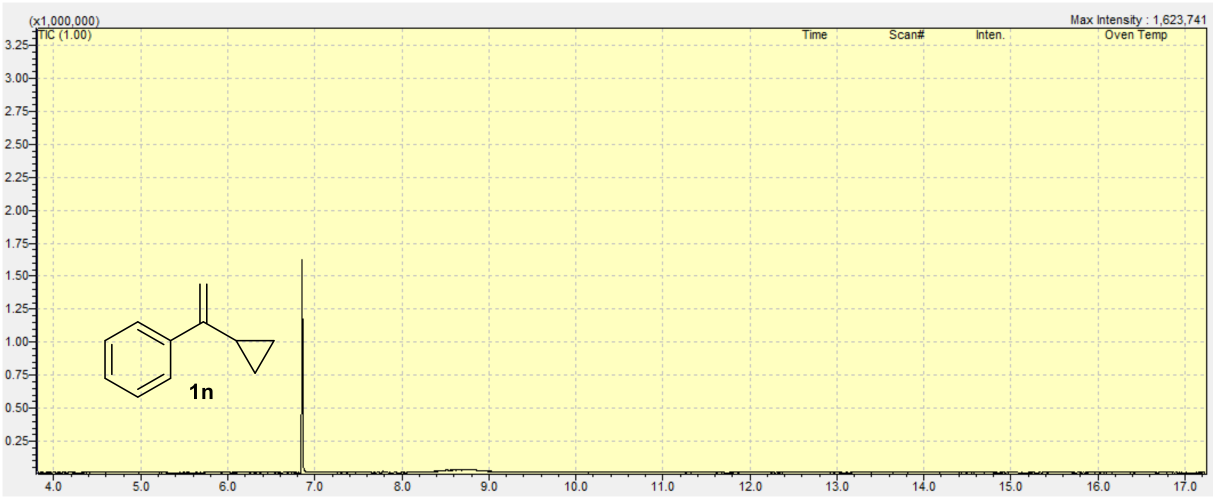

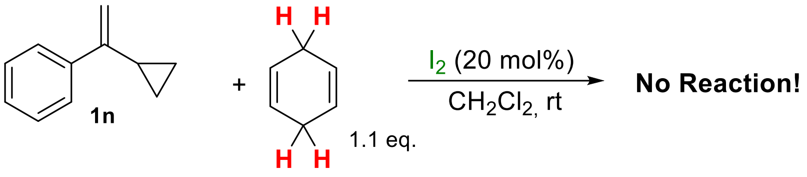

c) GCMS, in the dark

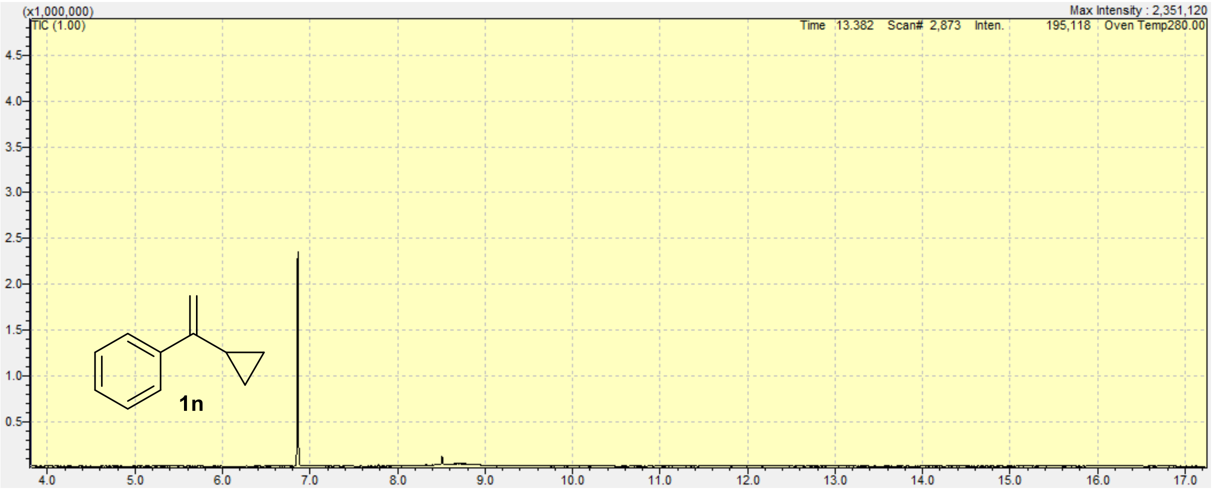

Optimised conditions:

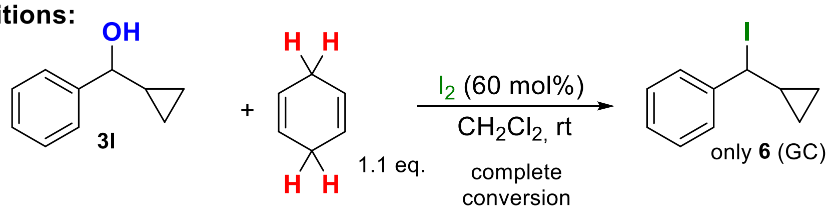

In the dark:

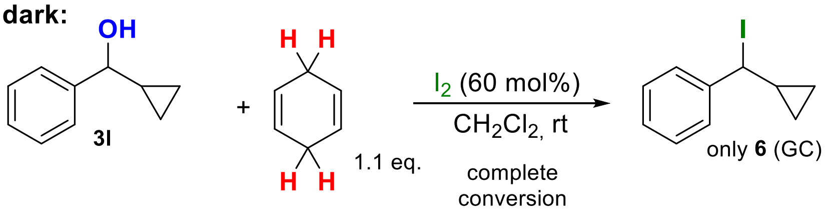

GCMS of **3l**

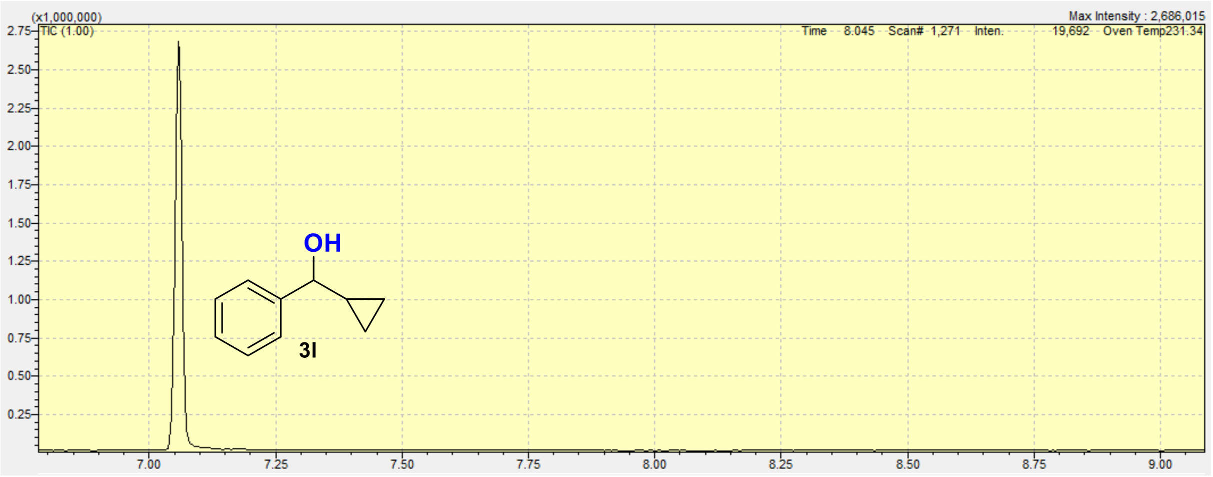

GCMS after the reactions

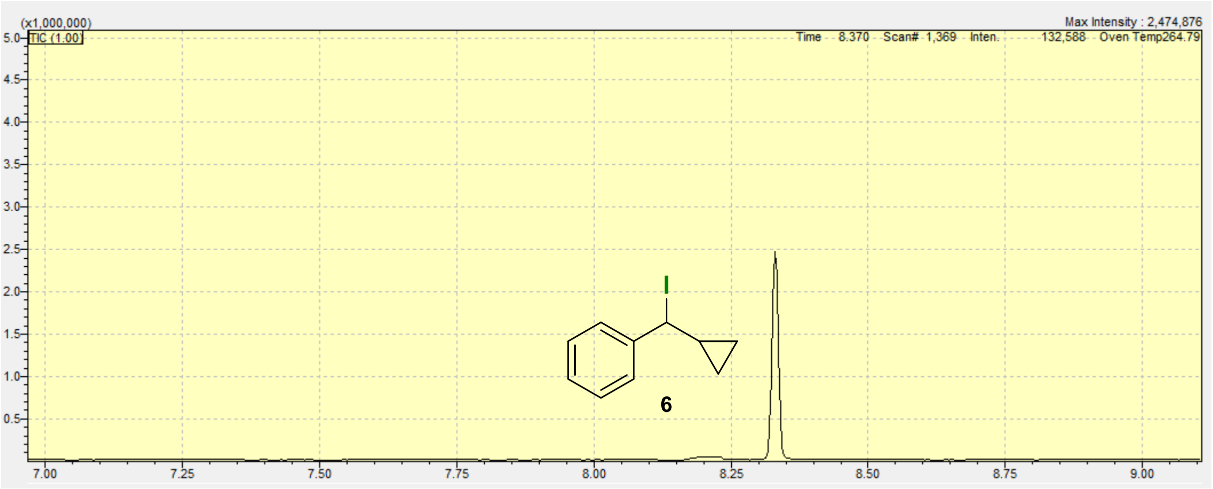

5. NMR spectra of all synthesised products

Ethane-1,1-diyldibenzene (2a, 4d)

^{1}H-NMR spectrum in $CDCl_3$ at 500 MHz and rt.

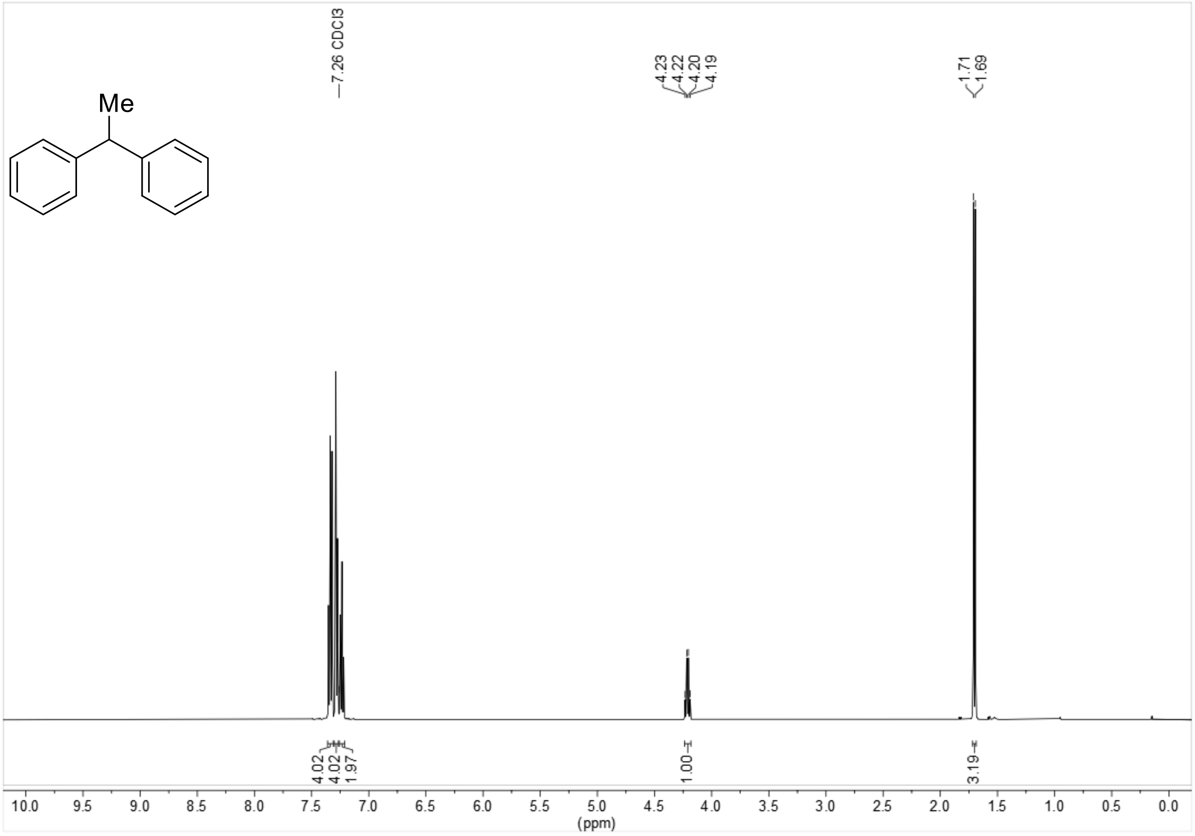

^{13}C-NMR spectrum in $CDCl_3$ at 125 MHz and rt.

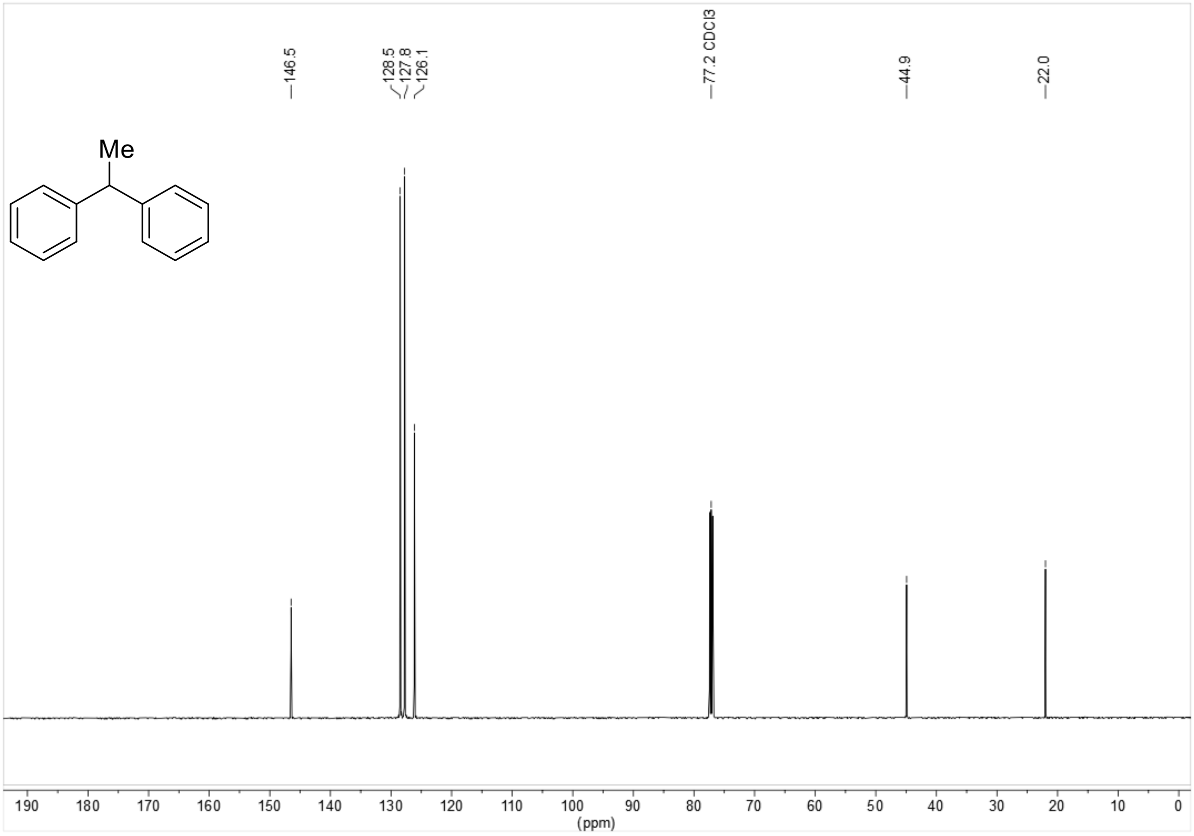

5-Methyl-10,11-dihydro-5H-dibenzo[a,d][7]annulene (2b)

^{1}H-NMR spectrum in $CDCl_3$ at 500 MHz and rt.

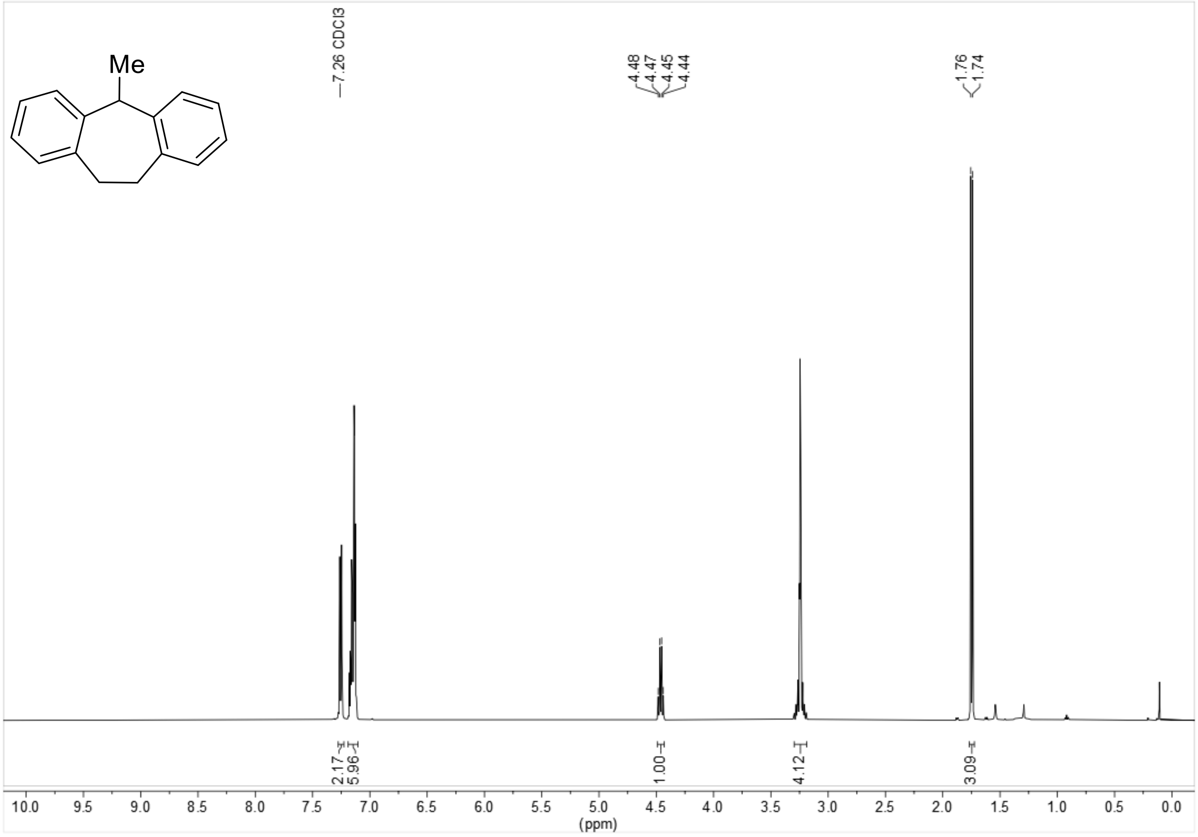

^{13}C-NMR spectrum in $CDCl_3$ at 125 MHz and rt.

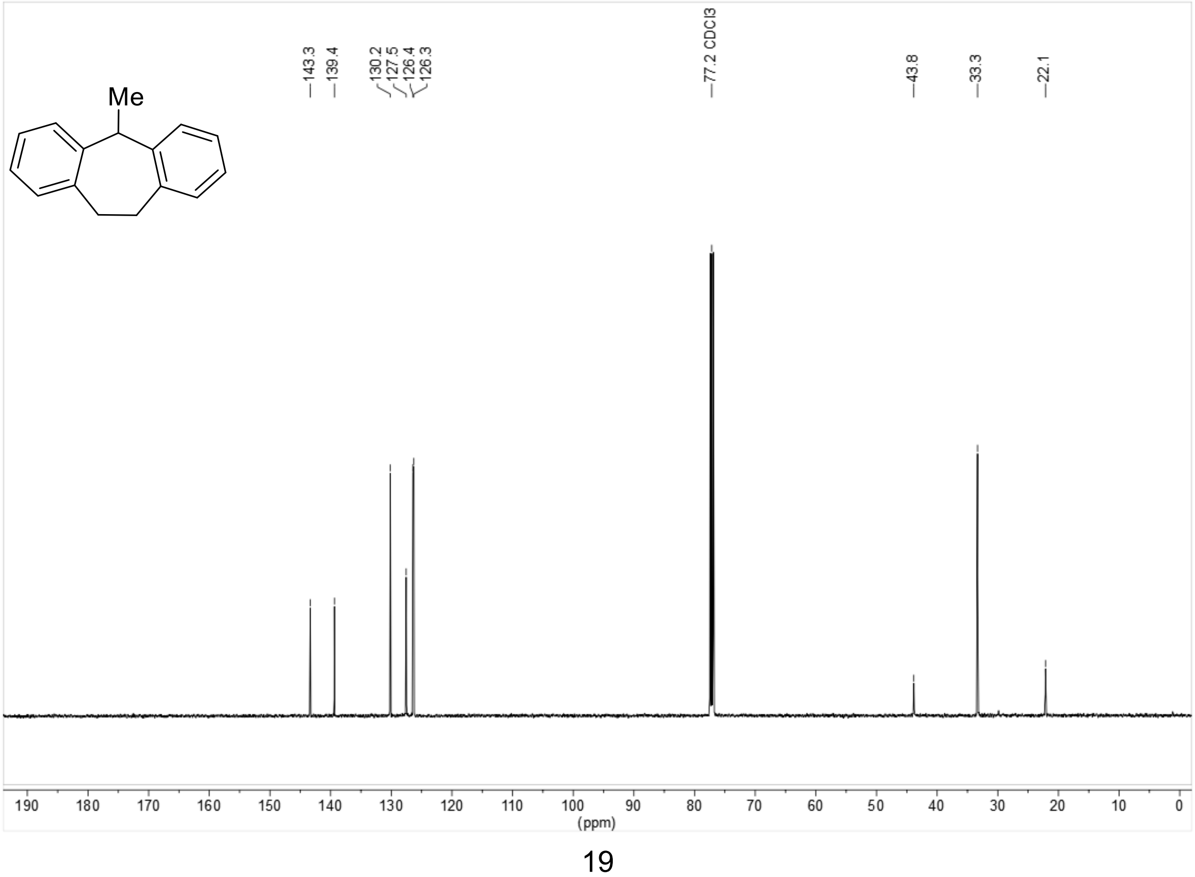

1,1-Di(4-methylphenyl)ethane (2c)

^{1}H-NMR spectrum in $CDCl_3$ at 500 MHz and rt.

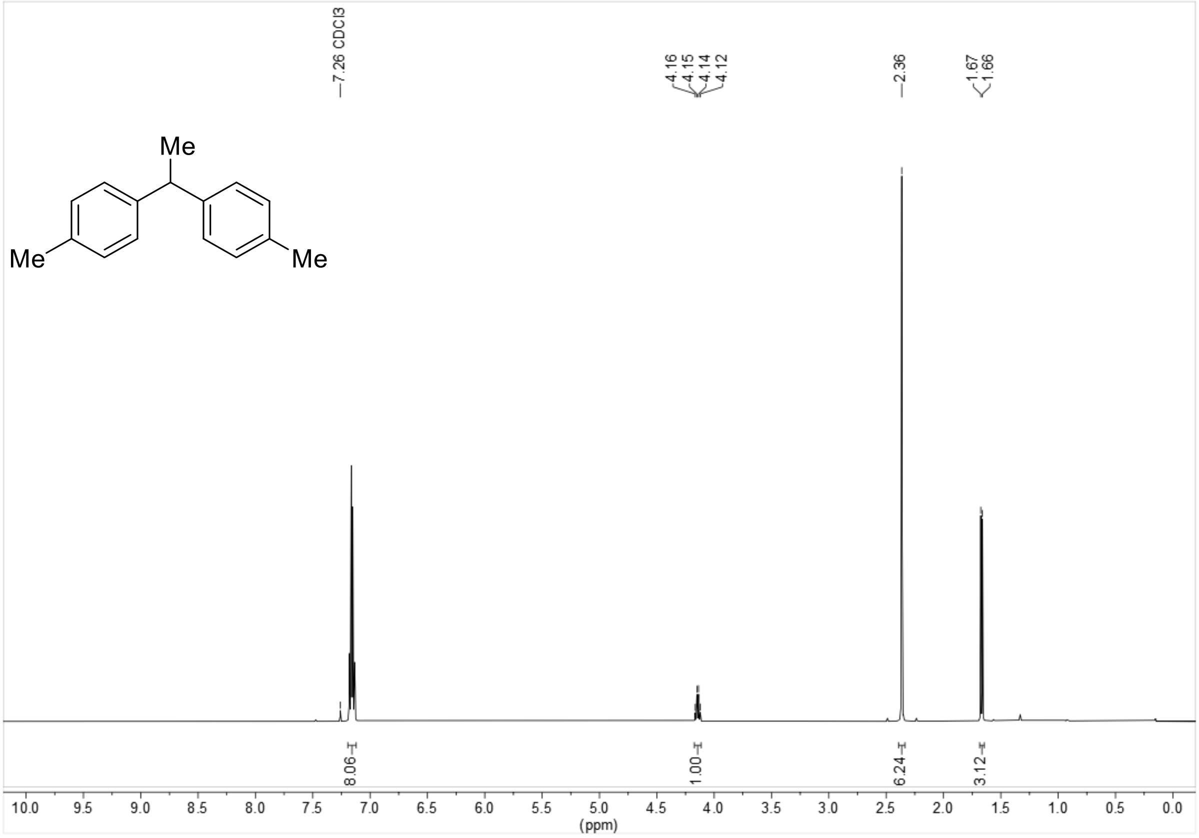

^{13}C-NMR spectrum in $CDCl_3$ at 125 MHz and rt.

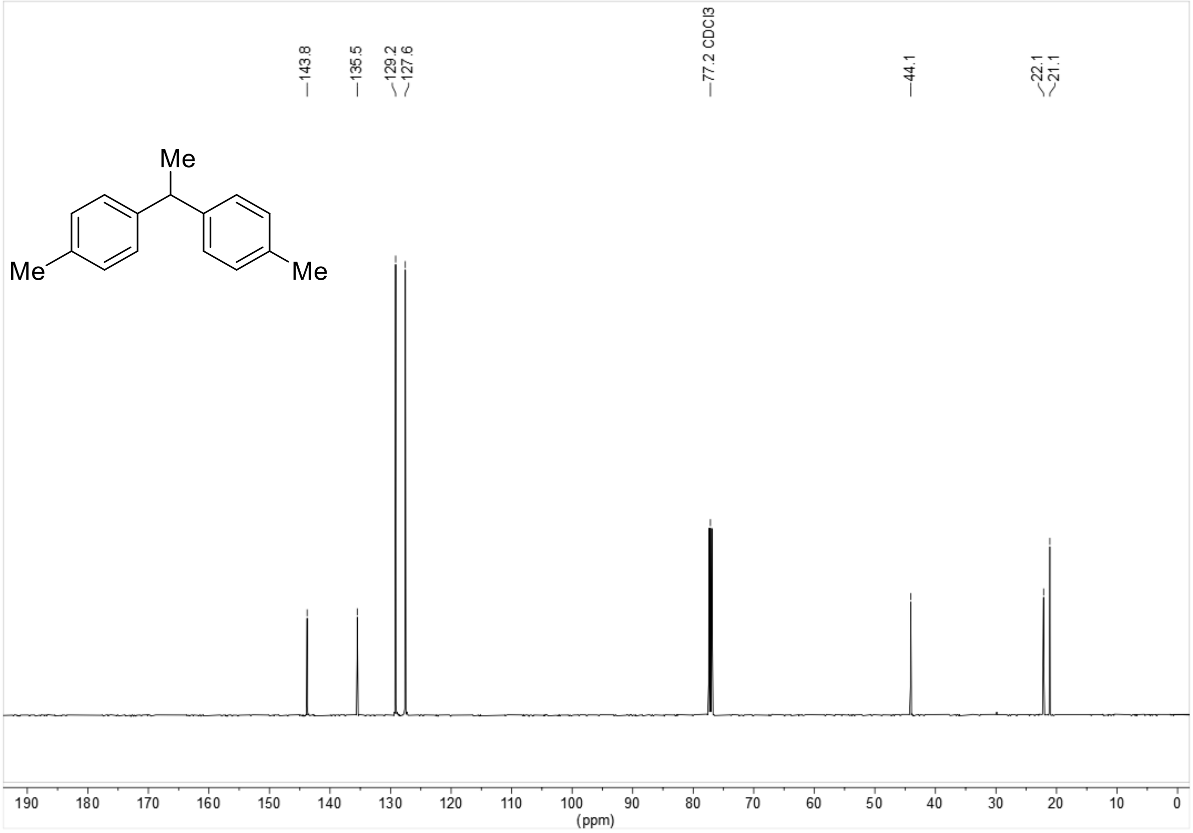

1-Methoxy-4-(1-phenylethyl)benzene (2d)

^{1}H-NMR spectrum in $CDCl_3$ at 500 MHz and rt.

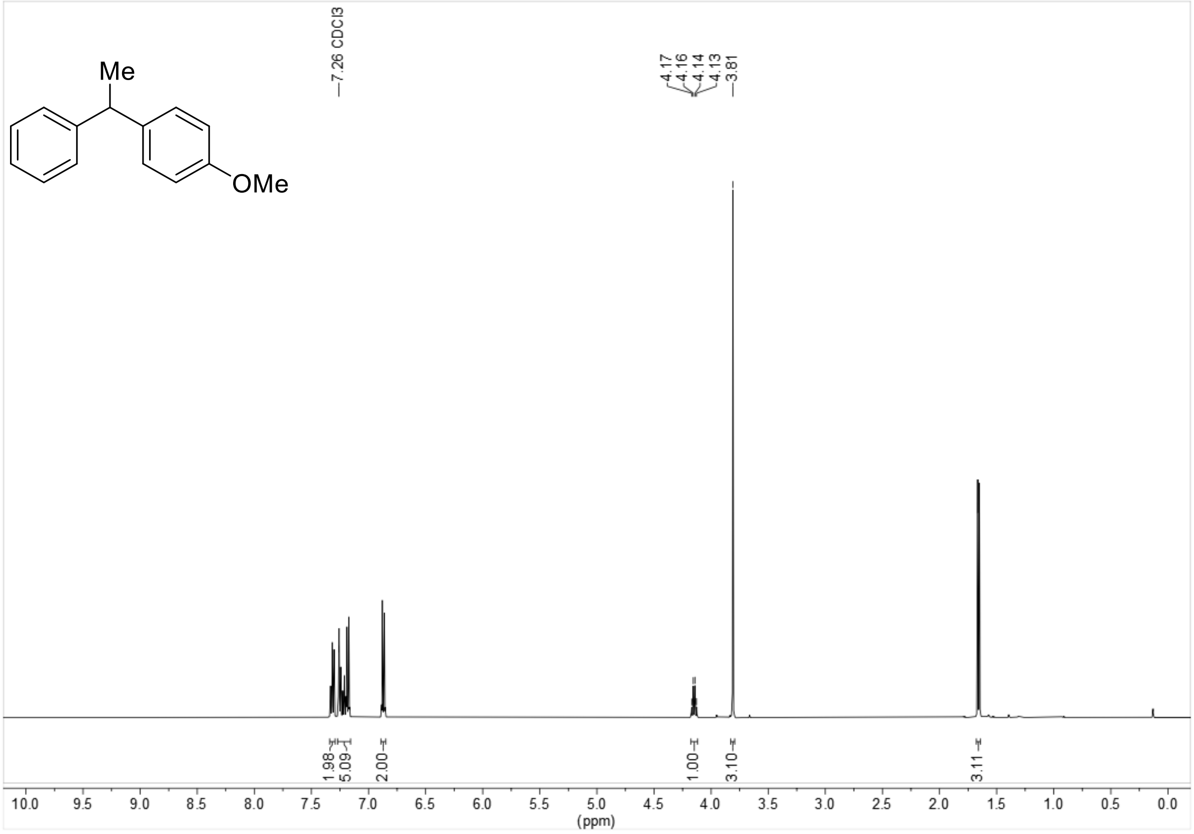

^{13}C-NMR spectrum in $CDCl_3$ at 125 MHz and rt.

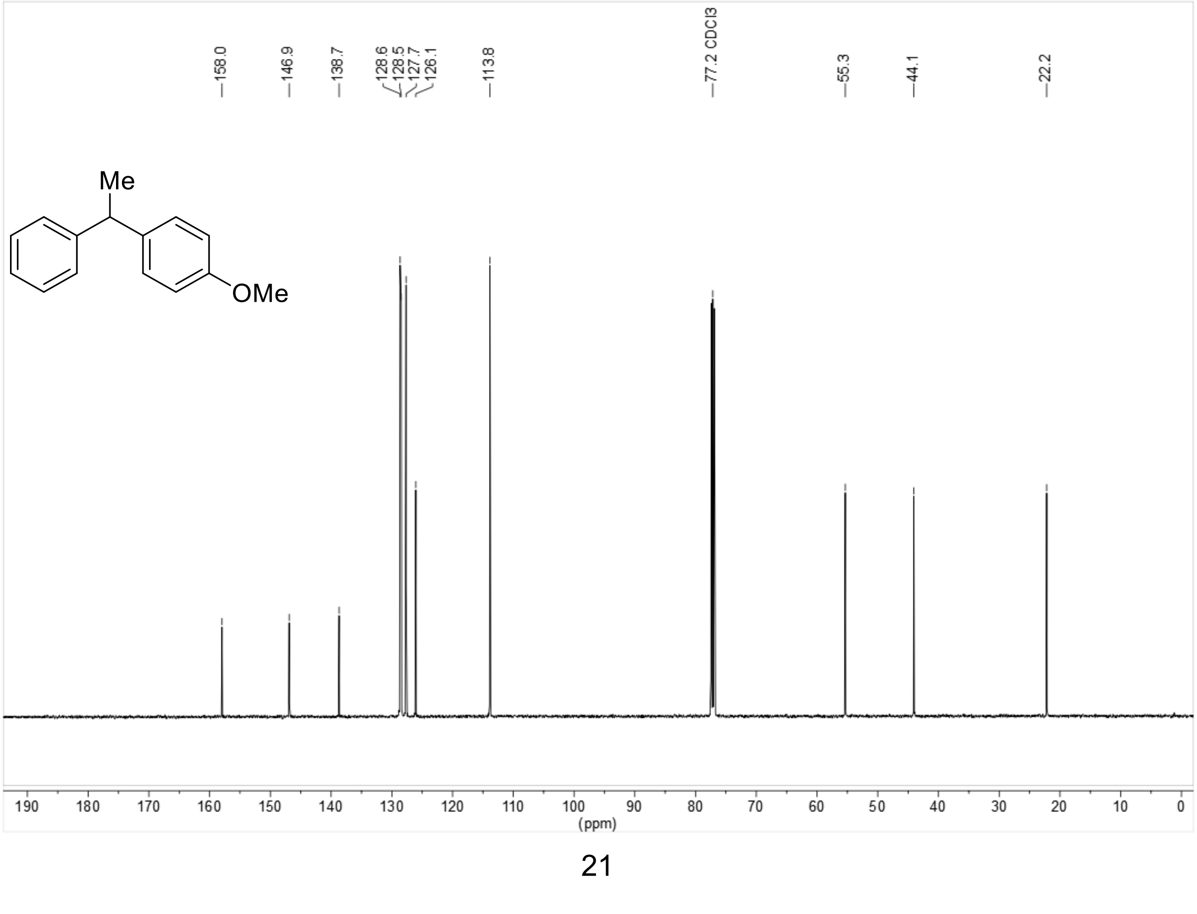

1-Methoxy-4-(1-(p-tolyl)ethyl)benzene (2e)

^{1}H-NMR spectrum in $CDCl_3$ at 500 MHz and rt.

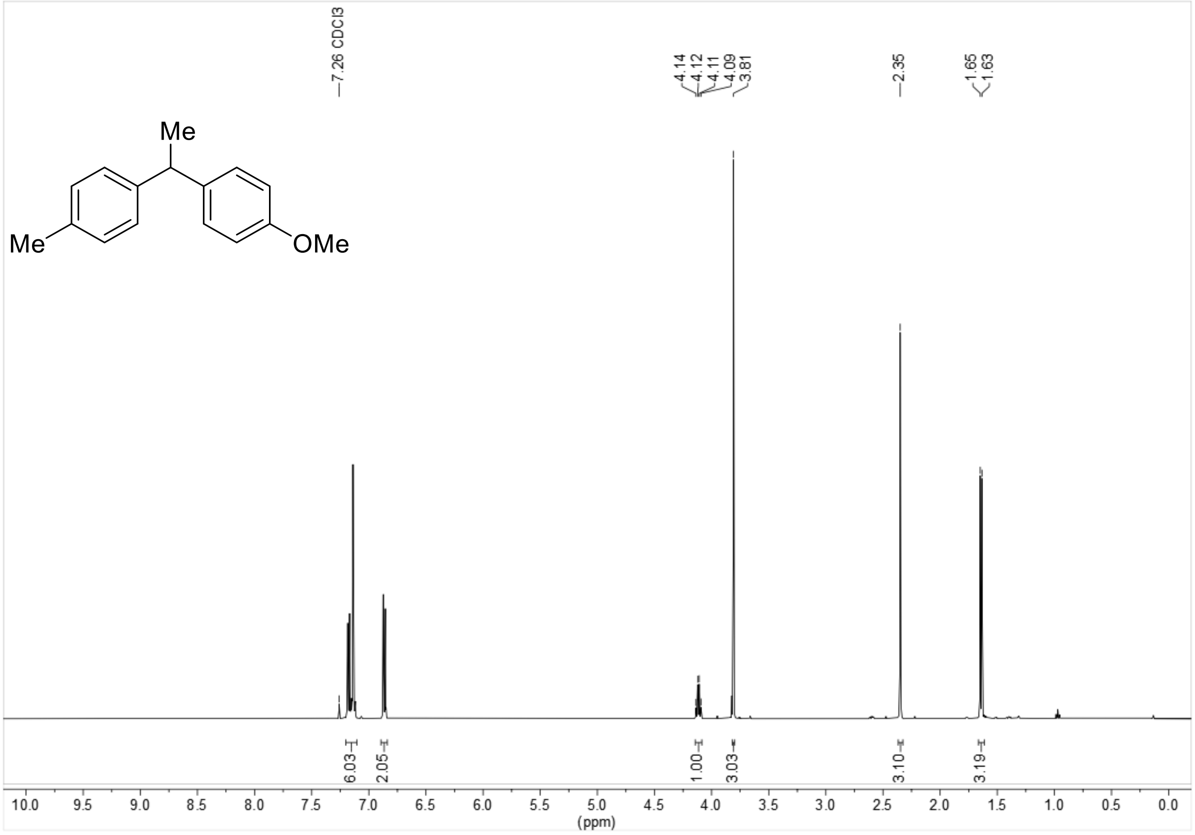

^{13}C-NMR spectrum in $CDCl_3$ at 125 MHz and rt.

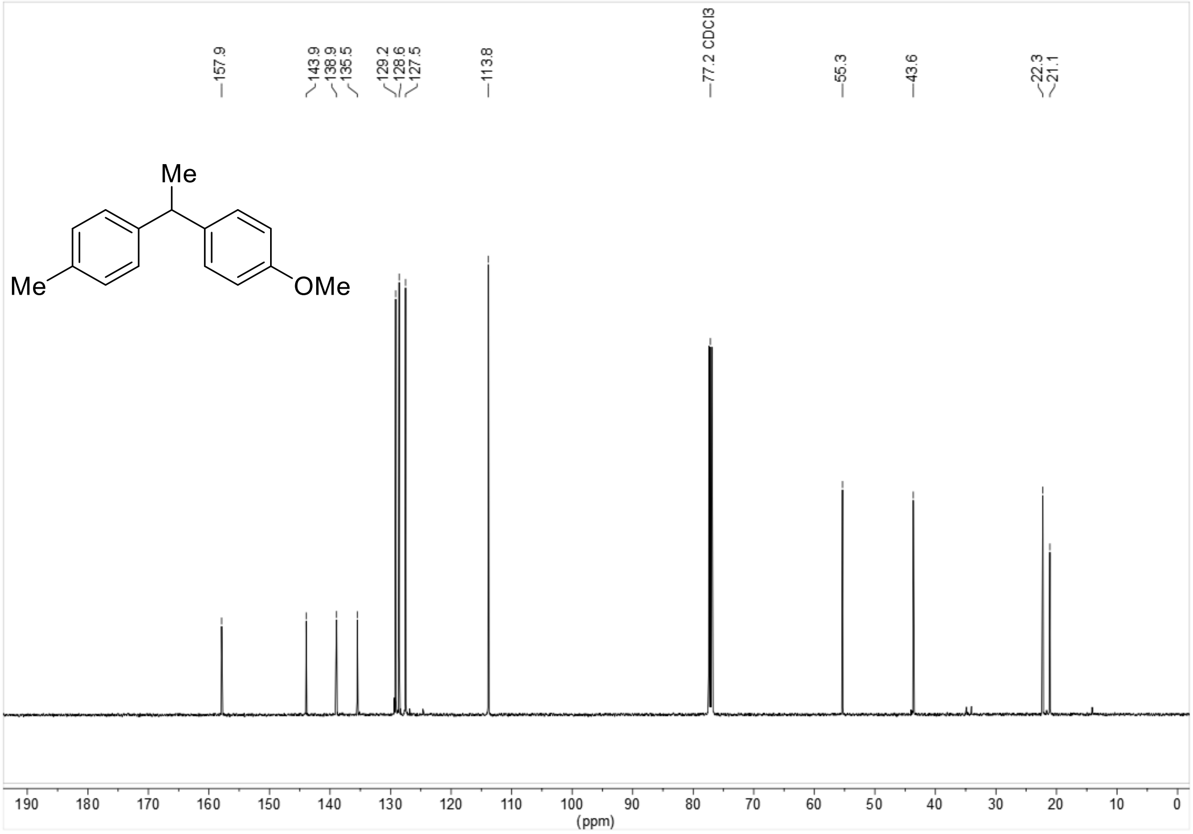

Methyl(4-(1-phenylethyl)phenyl)sulfane (2f)

^{1}H-NMR spectrum in $CDCl_3$ at 500 MHz and rt.

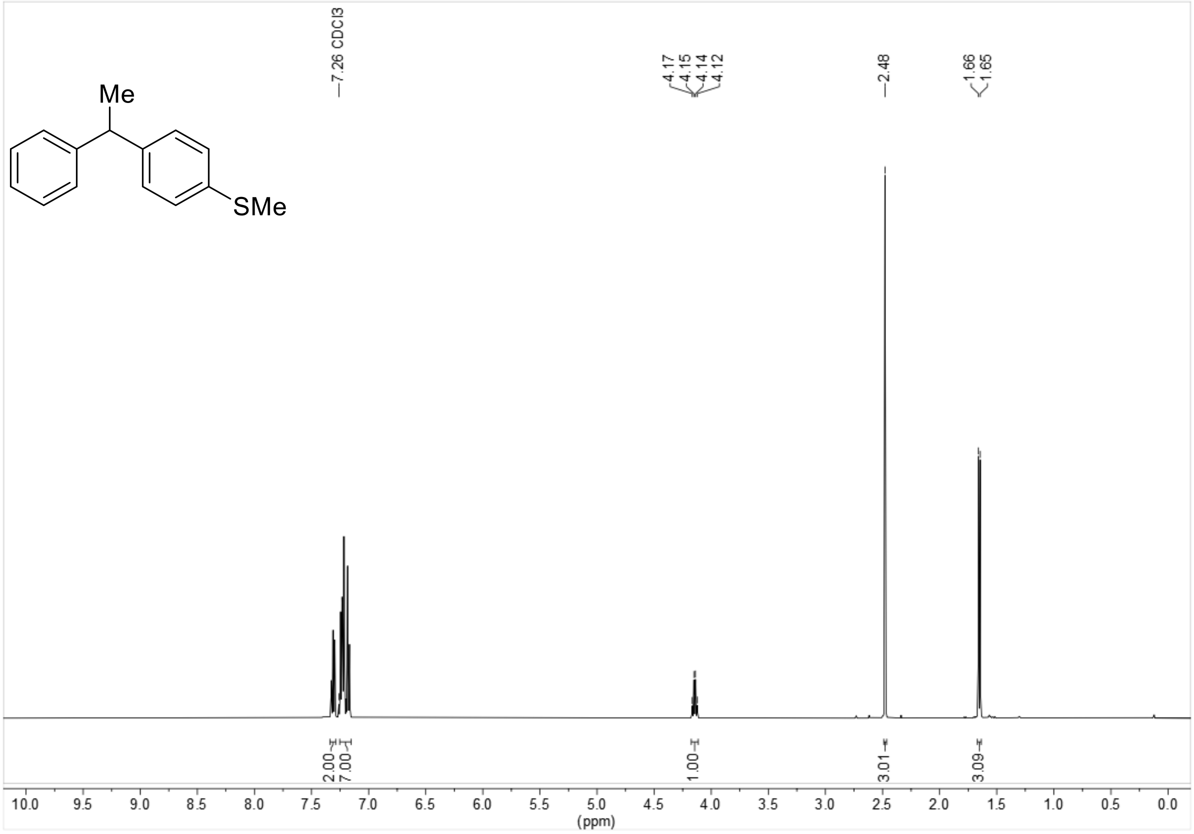

^{13}C-NMR spectrum in $CDCl_3$ at 125 MHz and rt.

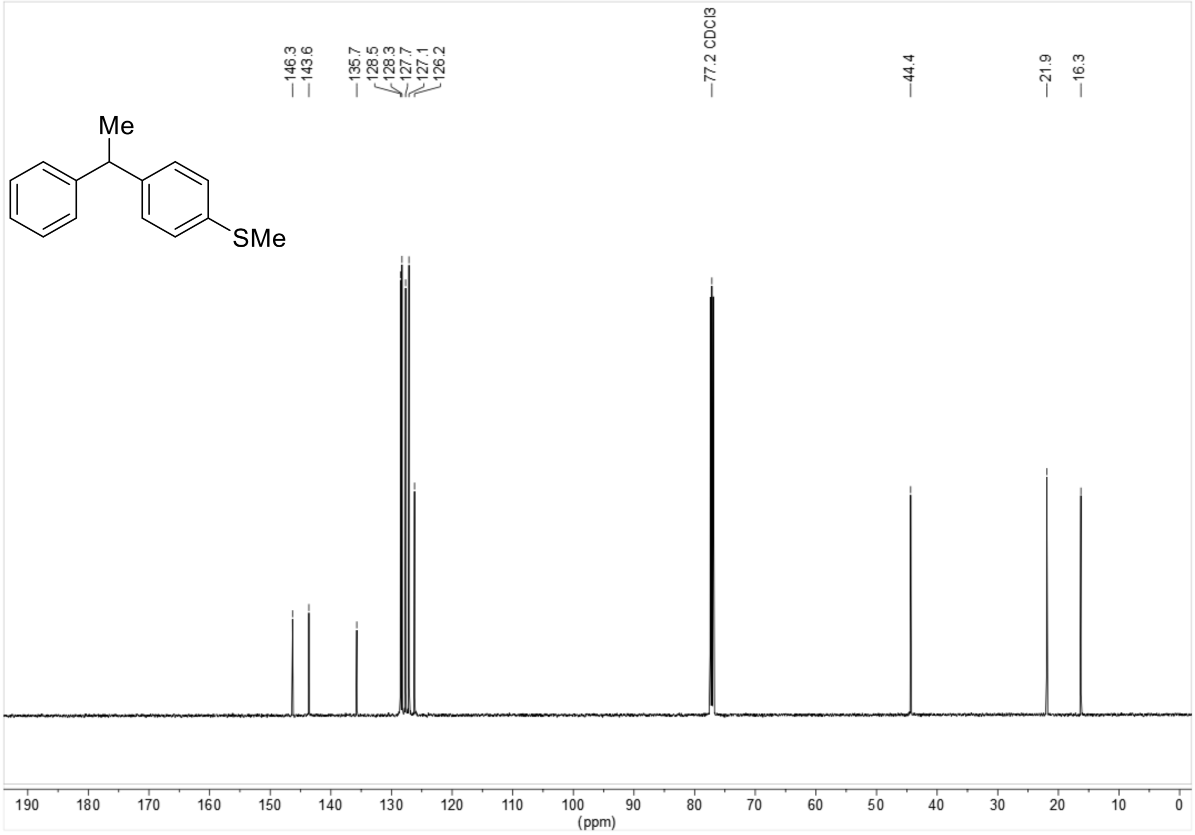

4,4'-(Ethane-1,1-diyl)bis(fluorobenzene) (2g)

^{1}H-NMR spectrum in $CDCl_3$ at 500 MHz and rt.

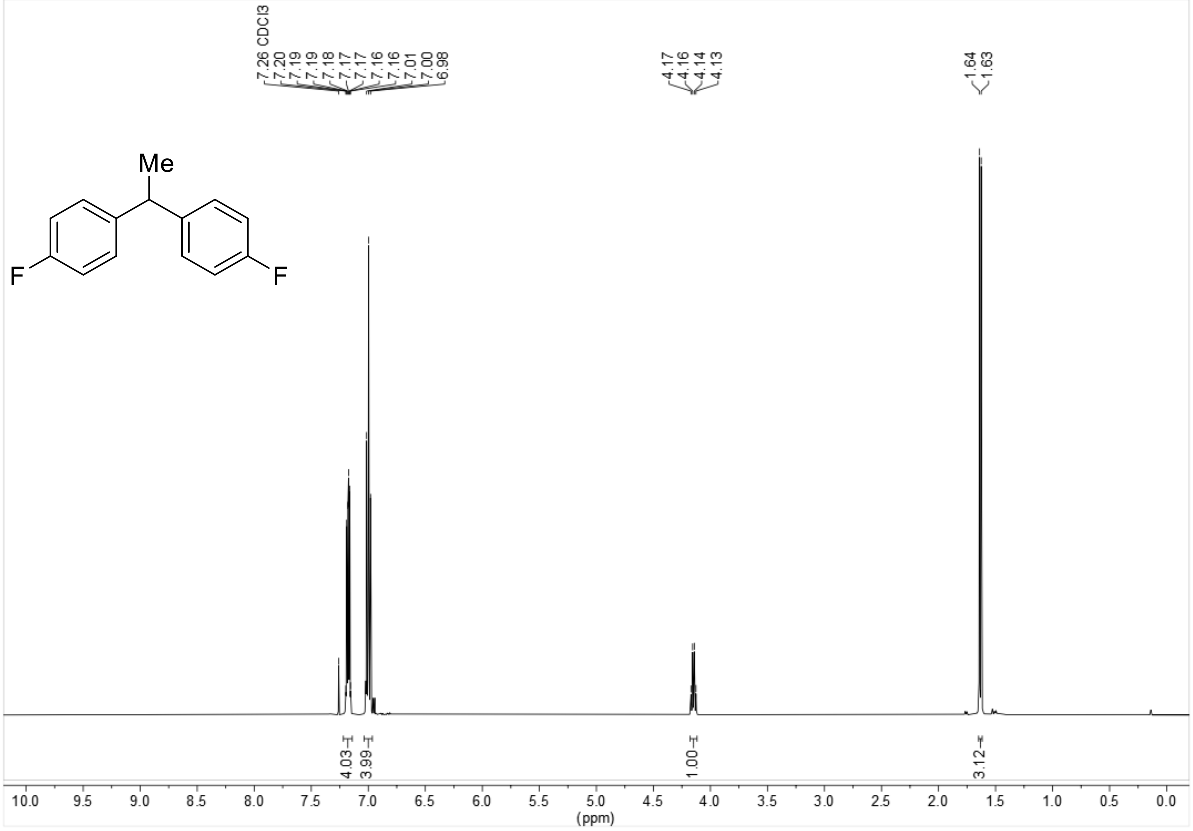

^{13}C-NMR spectrum in $CDCl_3$ at 125 MHz and rt.

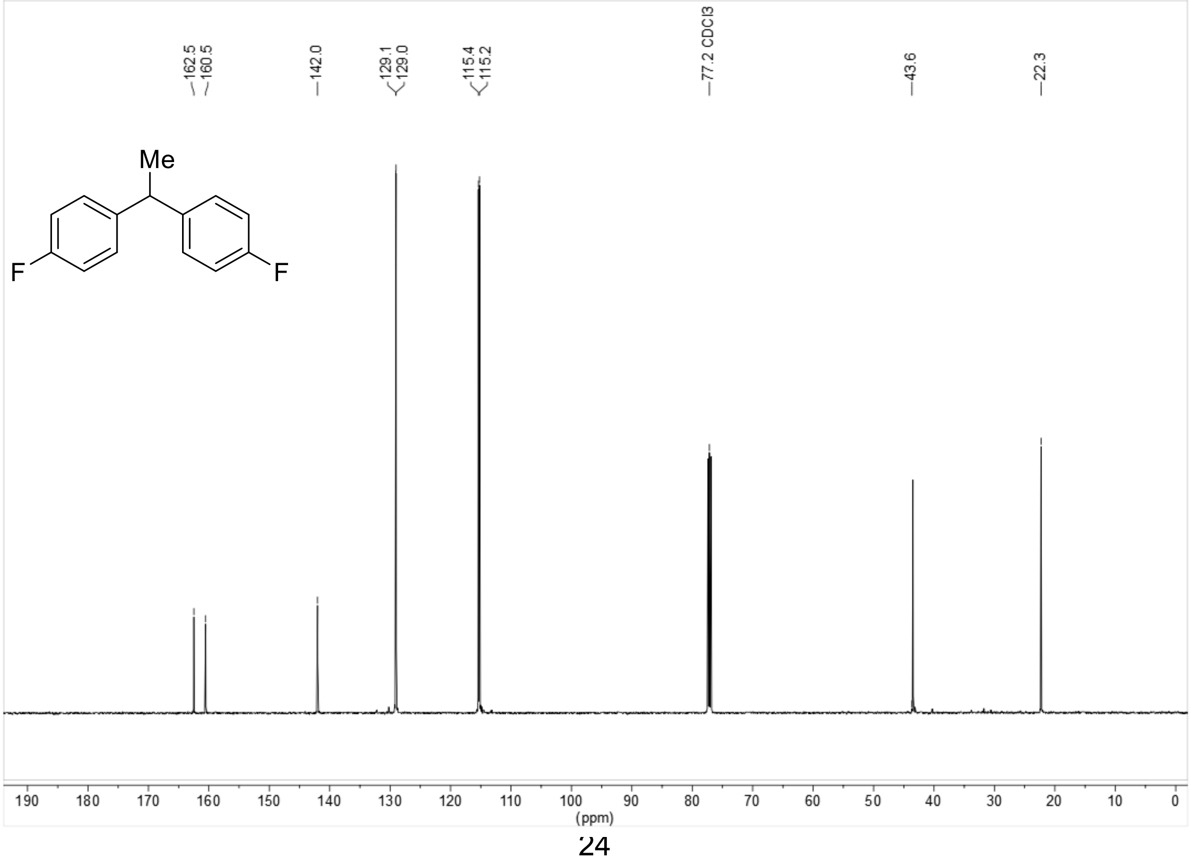

^{19}F-NMR spectrum in $CDCl_3$ at 470 MHz and rt.

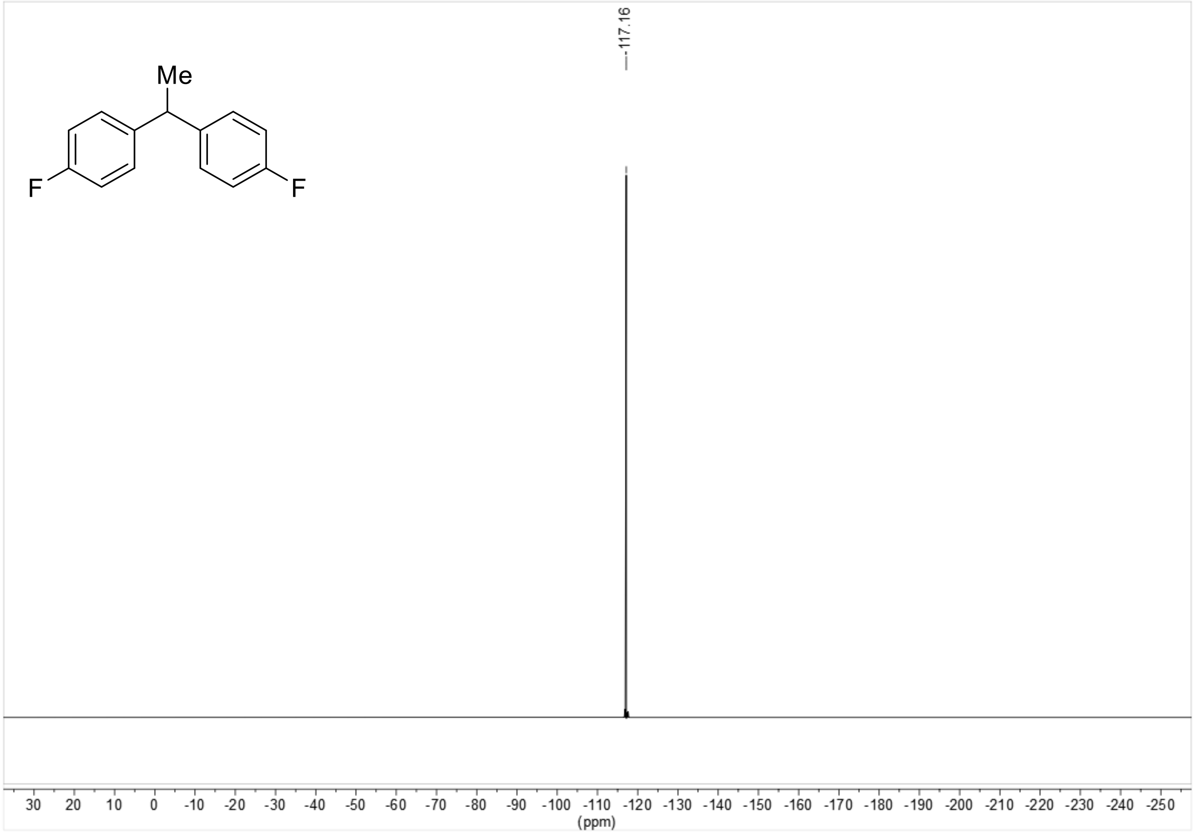

1-Chloro-4-(1-phenylethyl)benzene (2h)

^{1}H-NMR spectrum in $CDCl_3$ at 500 MHz and rt.

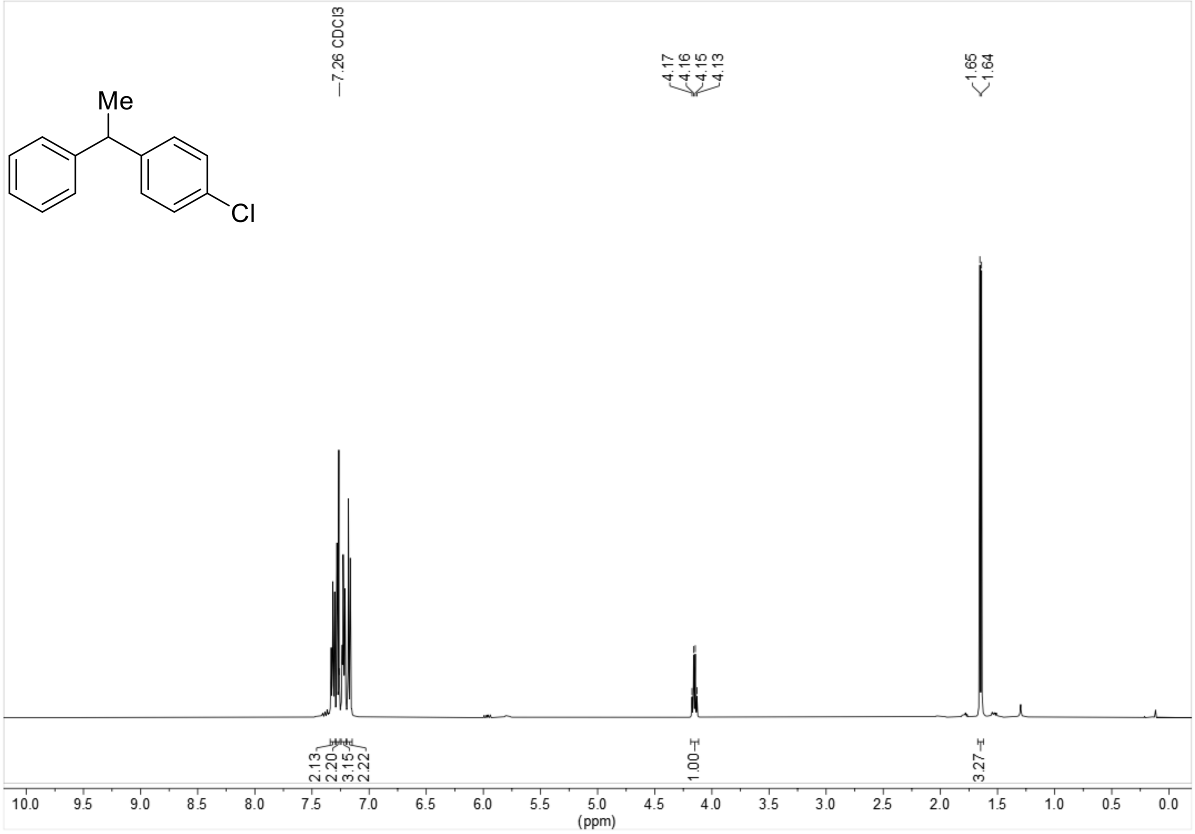

^{13}C-NMR spectrum in $CDCl_3$ at 125 MHz and rt.

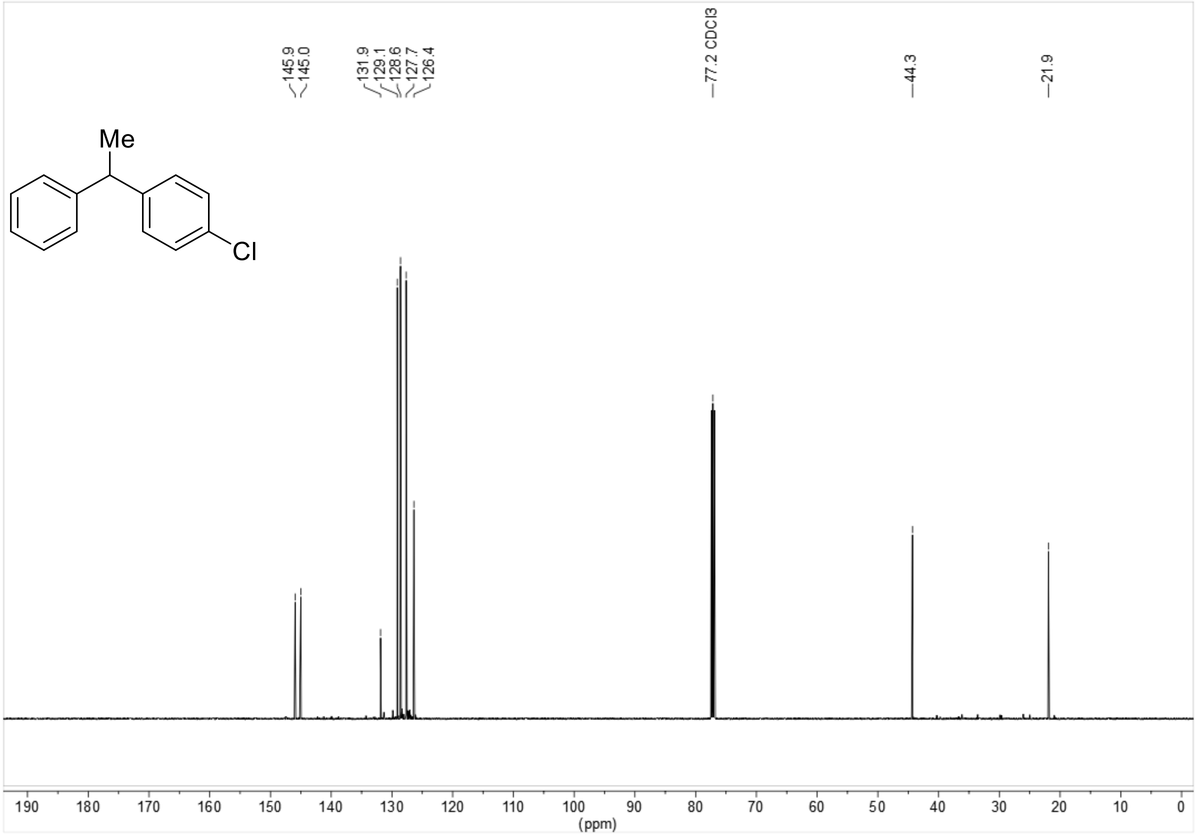

1-Bromo-3-(1-phenylethyl)benzene (2i)

^{1}H-NMR spectrum in $CDCl_3$ at 500 MHz and rt.

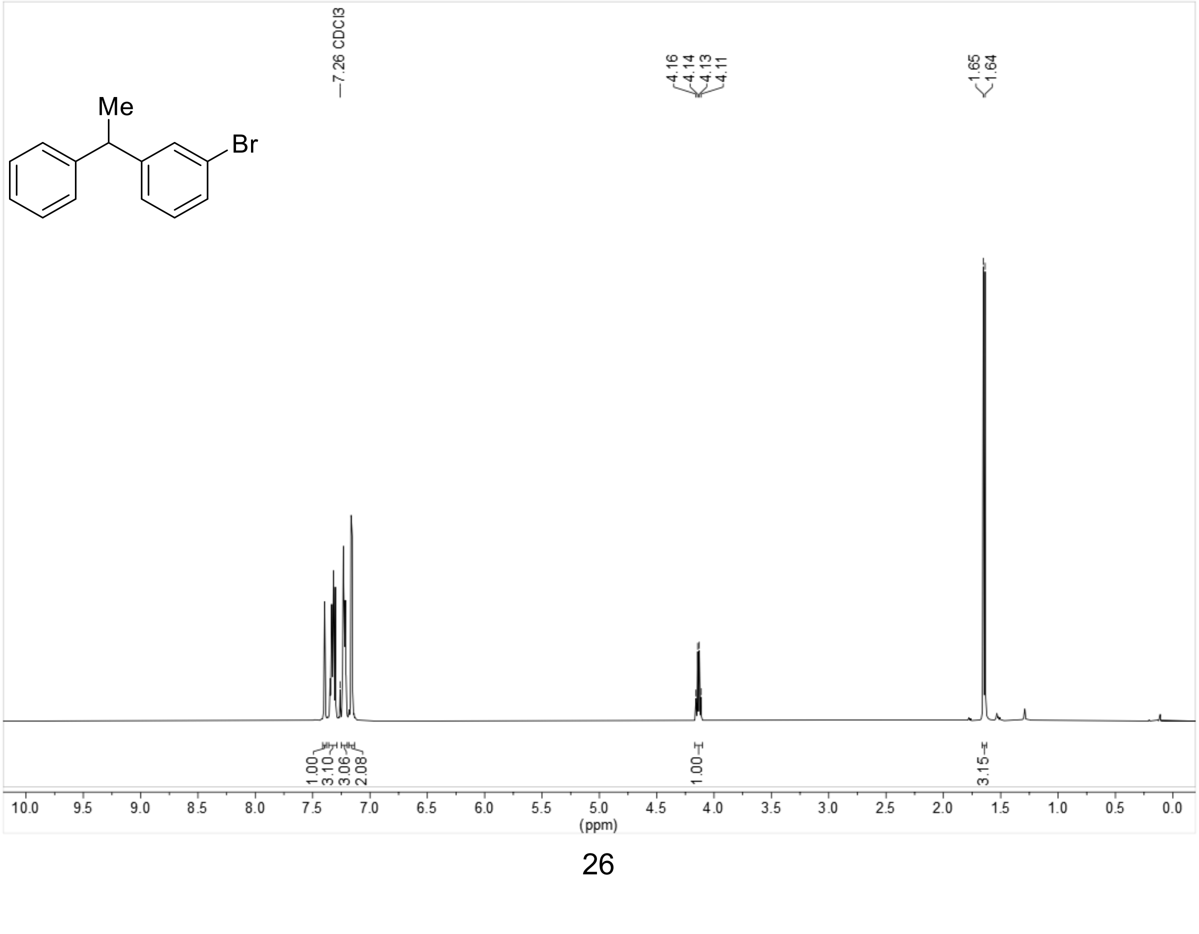

^{13}C-NMR spectrum in $CDCl_3$ at 125 MHz and rt.

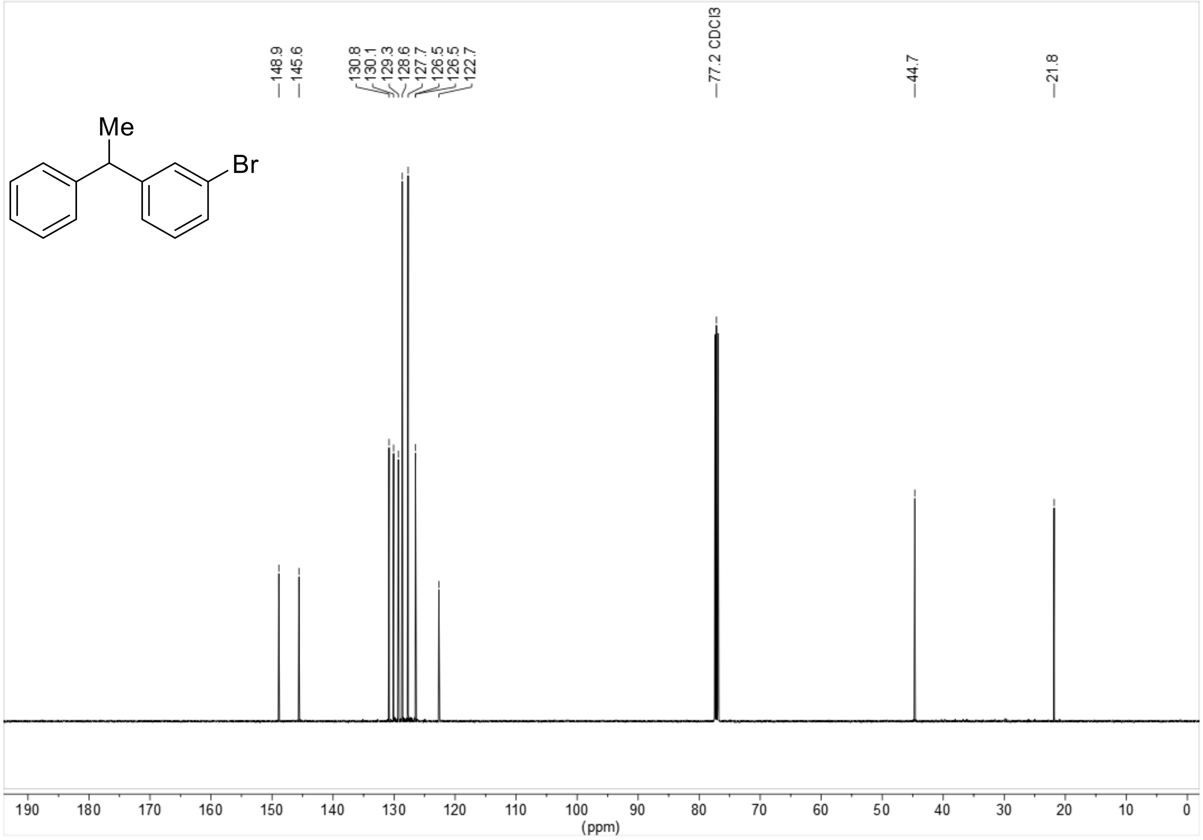

4,4’-(Ethane-1,1-diyl)bis(1-bromobenzene) (2j)

^{1}H-NMR spectrum in $CDCl_3$ at 500 MHz and rt.

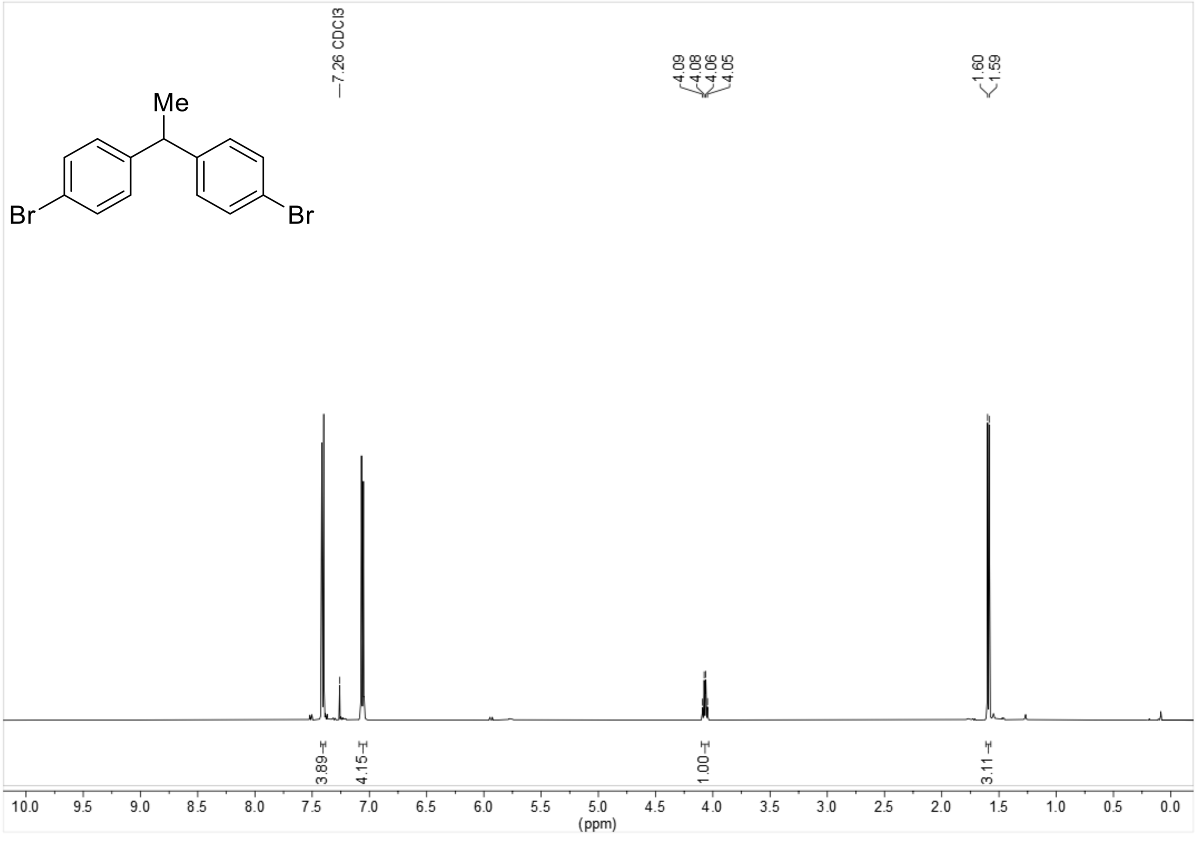

^{13}C-NMR spectrum in $CDCl_3$ at 125 MHz and rt.

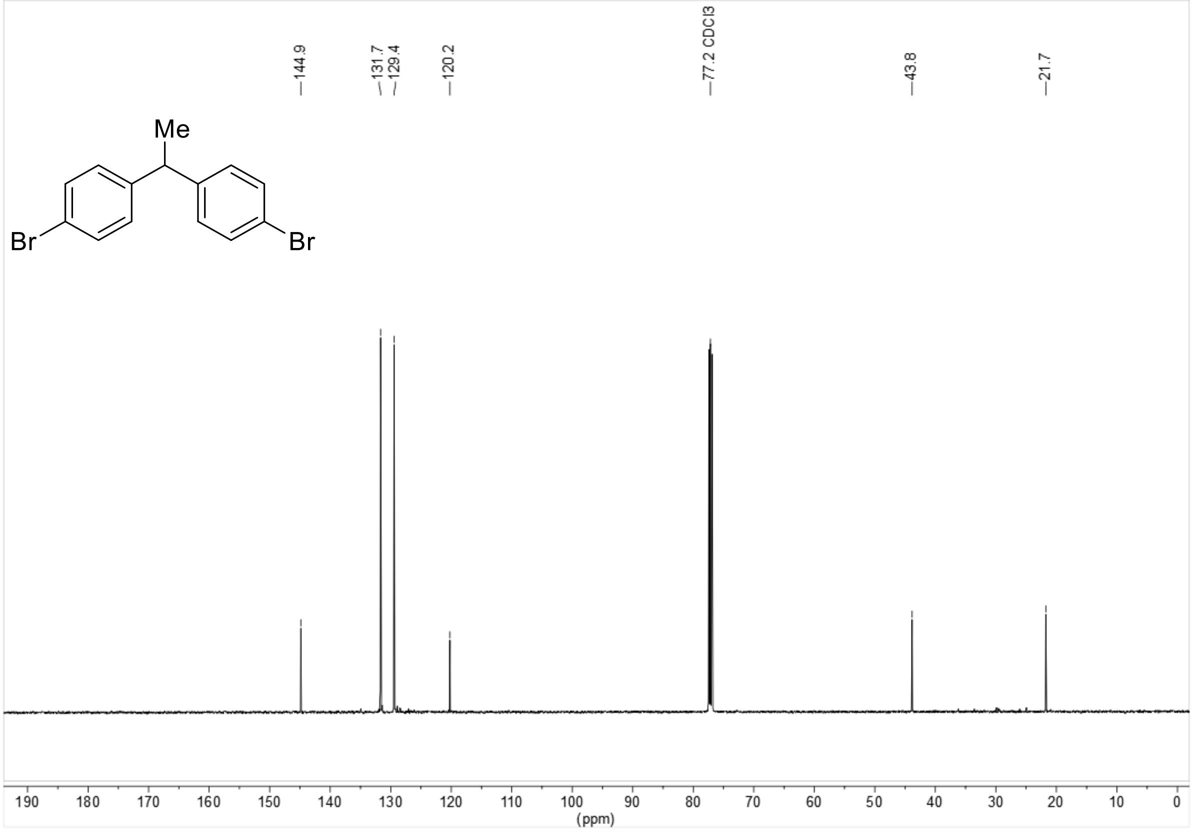

1-(1-Phenylethyl)-4-(trifluoromethyl)benzene (2k)

^{1}H-NMR spectrum in $CDCl_3$ at 500 MHz and rt.

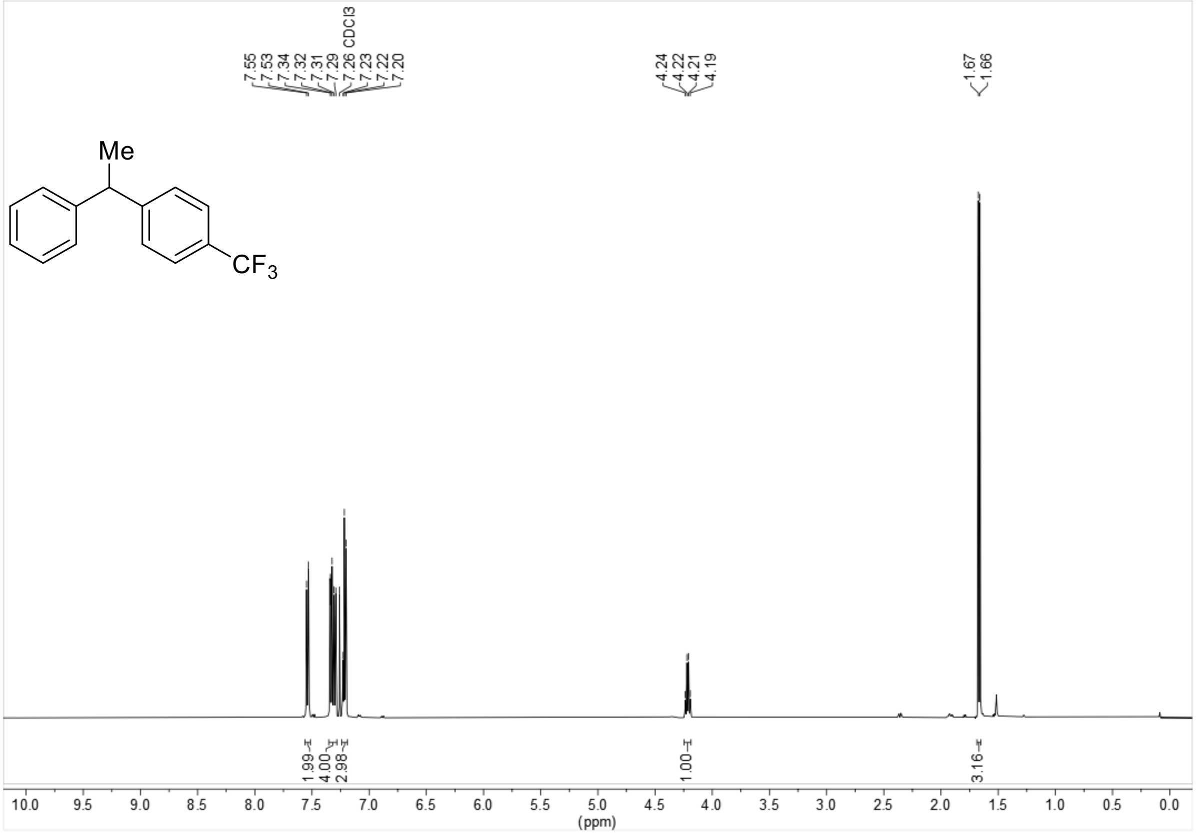

^{13}C-NMR spectrum in $CDCl_3$ at 125 MHz and rt.

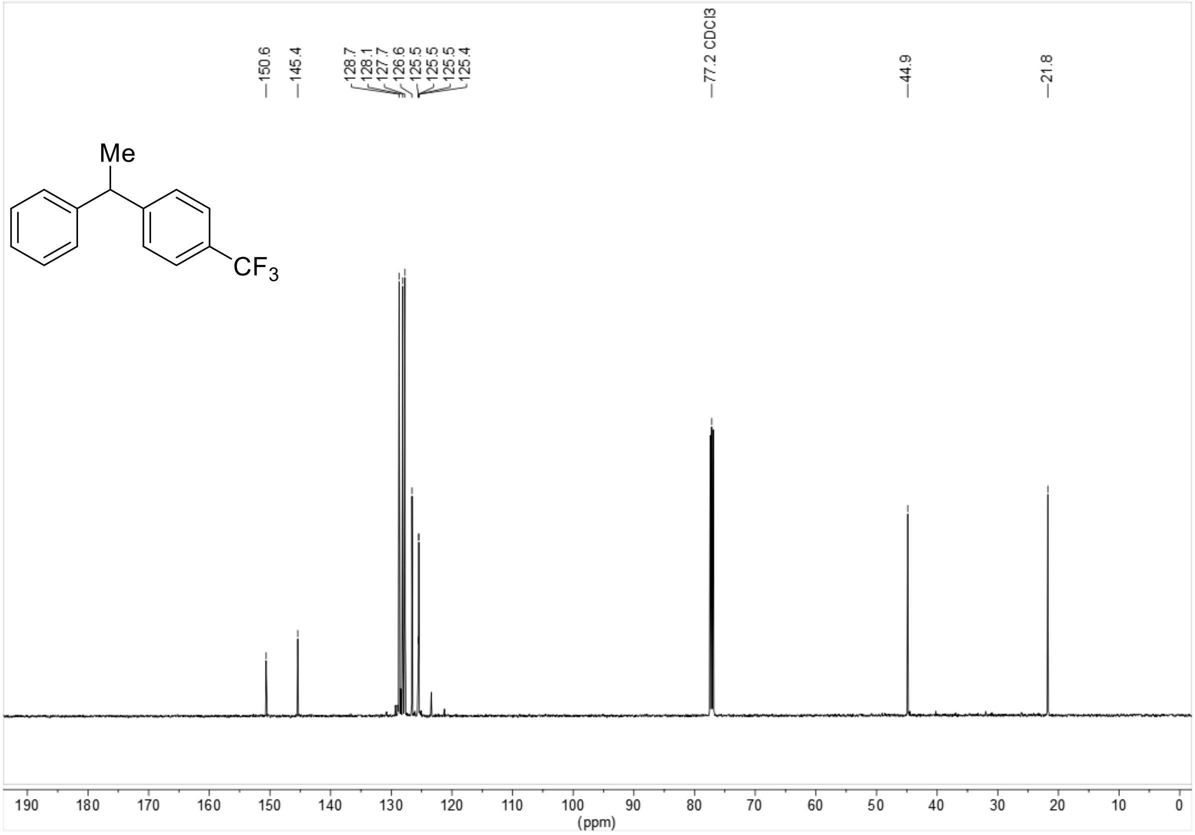

^{19}F-NMR spectrum in $CDCl_3$ at 470 MHz and rt.

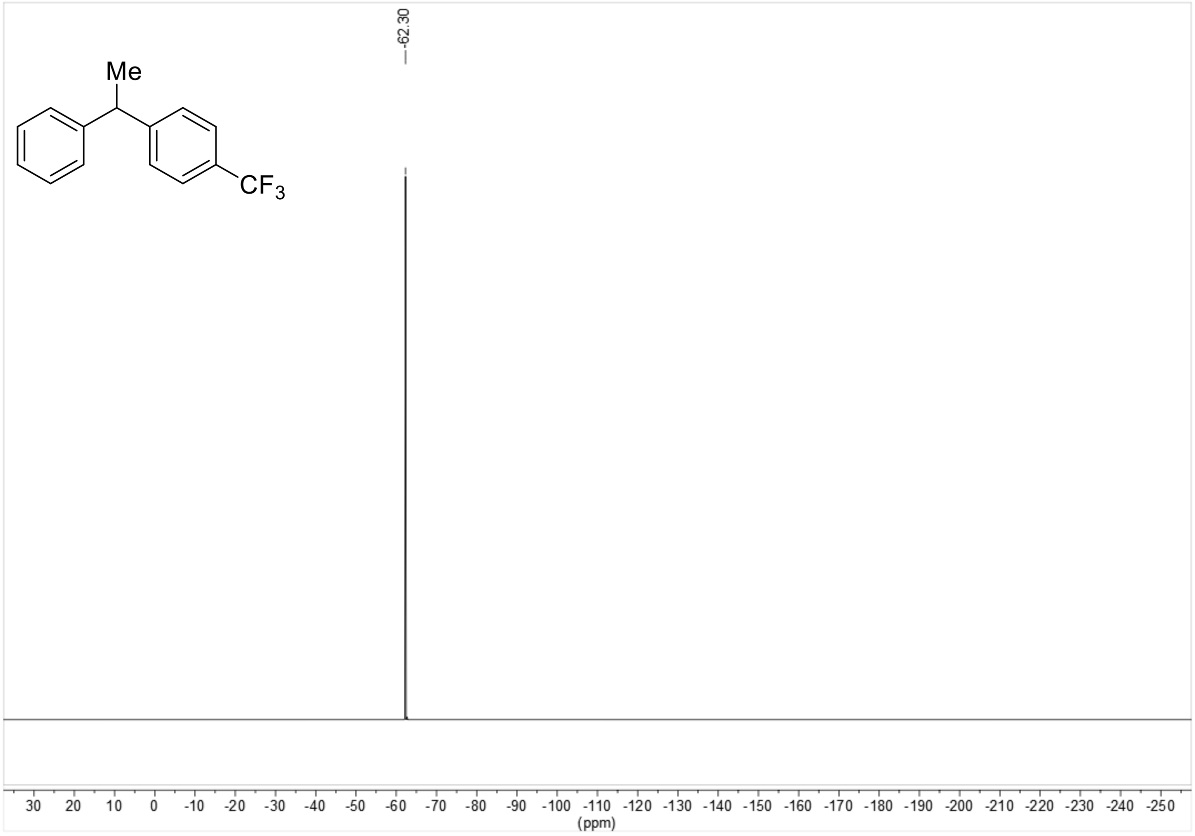

(1-Cyclohexylethyl)benzene (2l)

^{1}H-NMR spectrum in $CDCl_3$ at 500 MHz and rt.

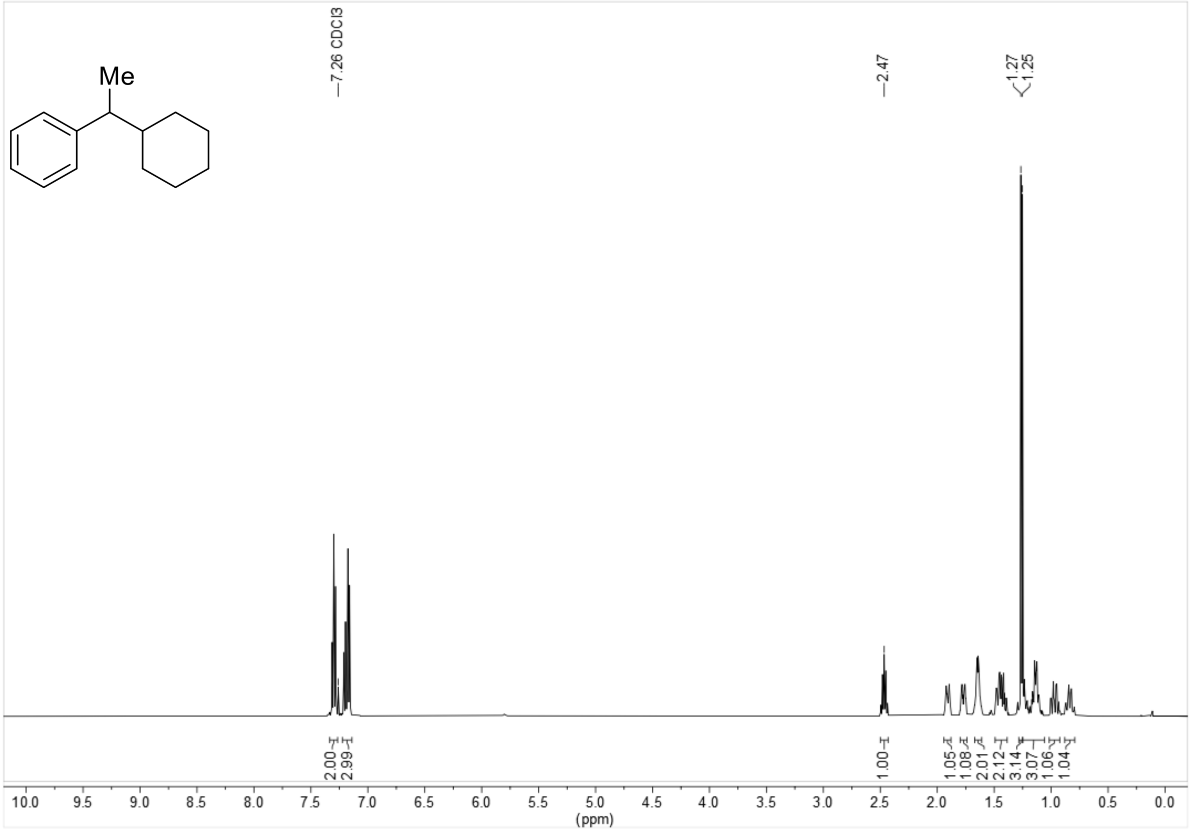

^{13}C-NMR spectrum in $CDCl_3$ at 125 MHz and rt.

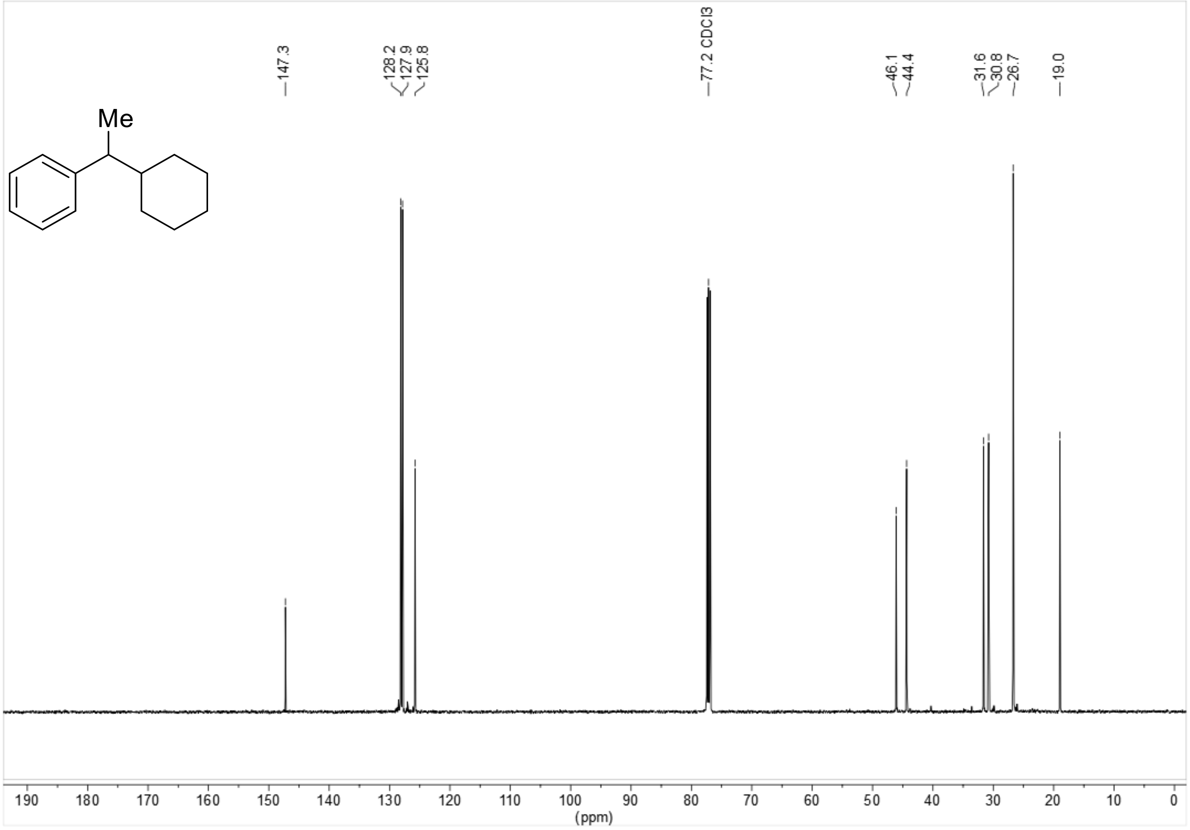

1-Phenyl-1-cyclohexane (2m, 4i)

^{1}H-NMR spectrum in $CDCl_3$ at 500 MHz and rt.

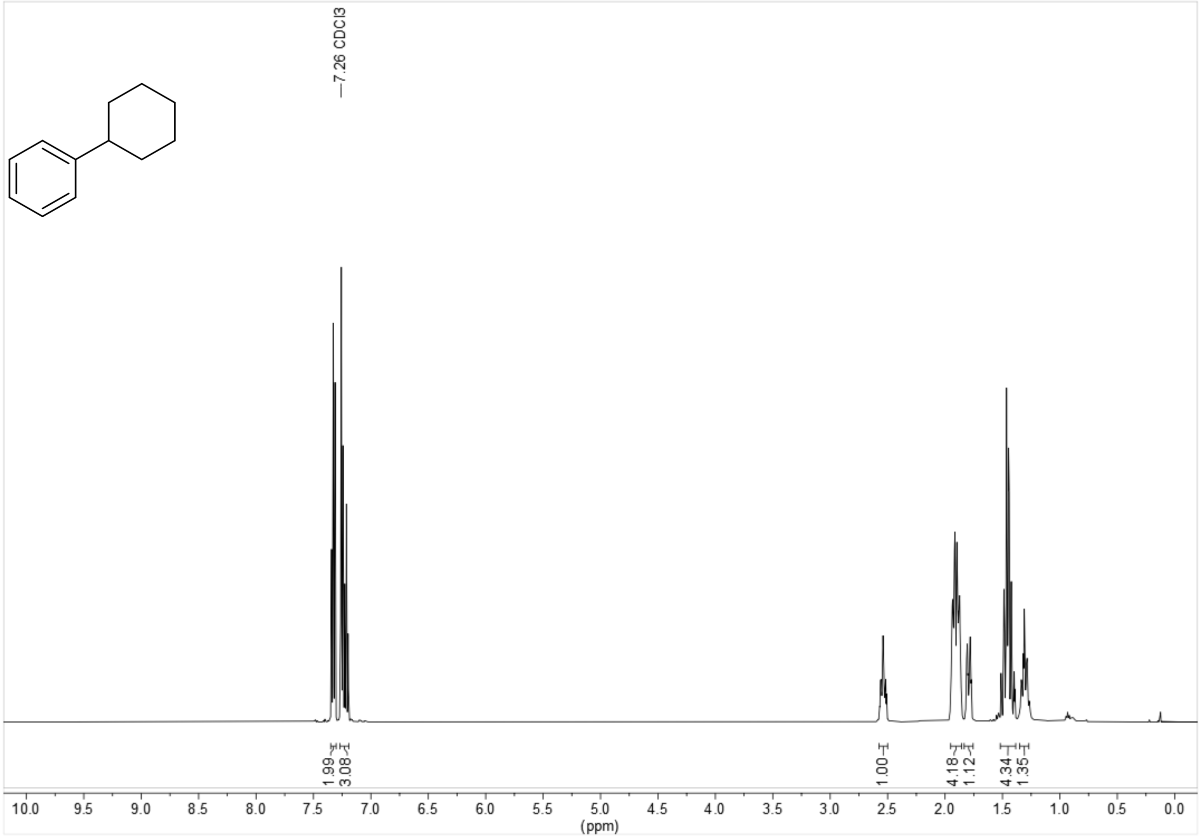

^{13}C-NMR spectrum in $CDCl_3$ at 125 MHz and rt.

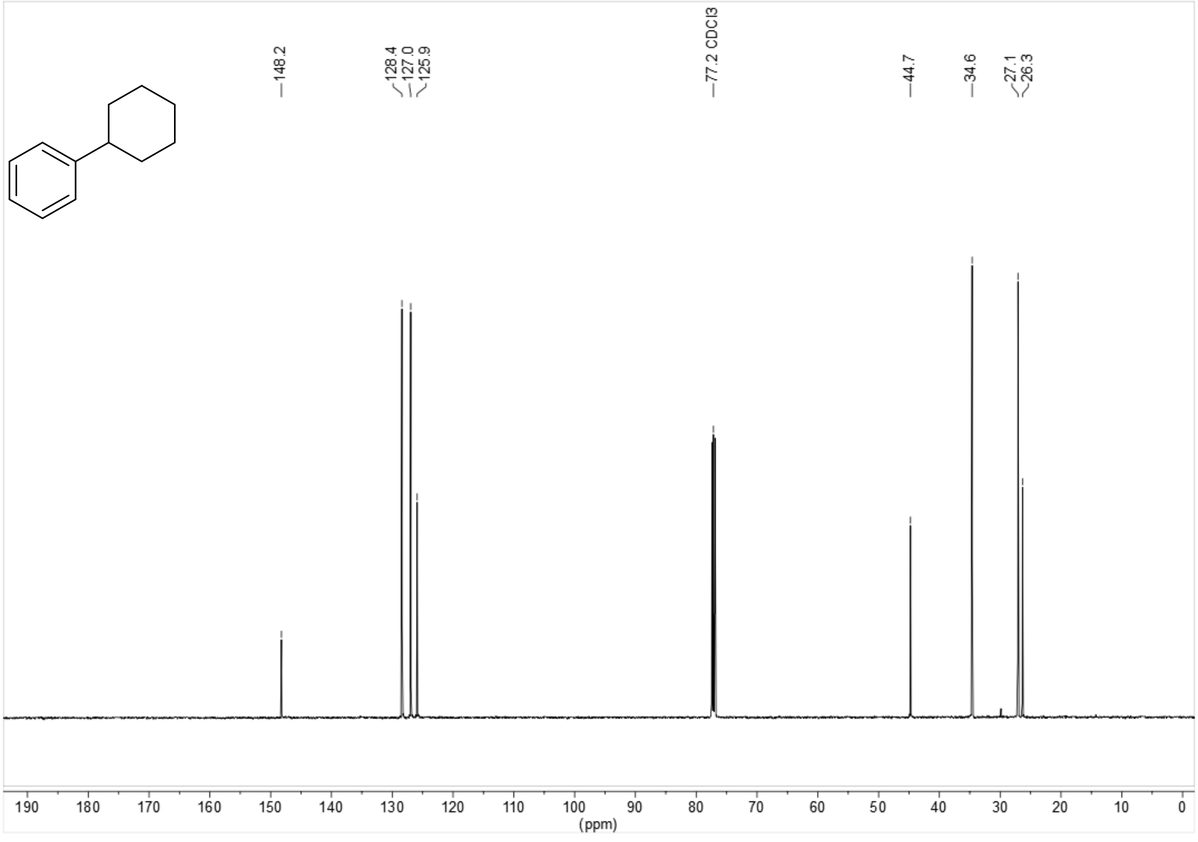

Diphenylmethane (4a)

^{1}H-NMR spectrum in $CDCl_3$ at 500 MHz and rt.

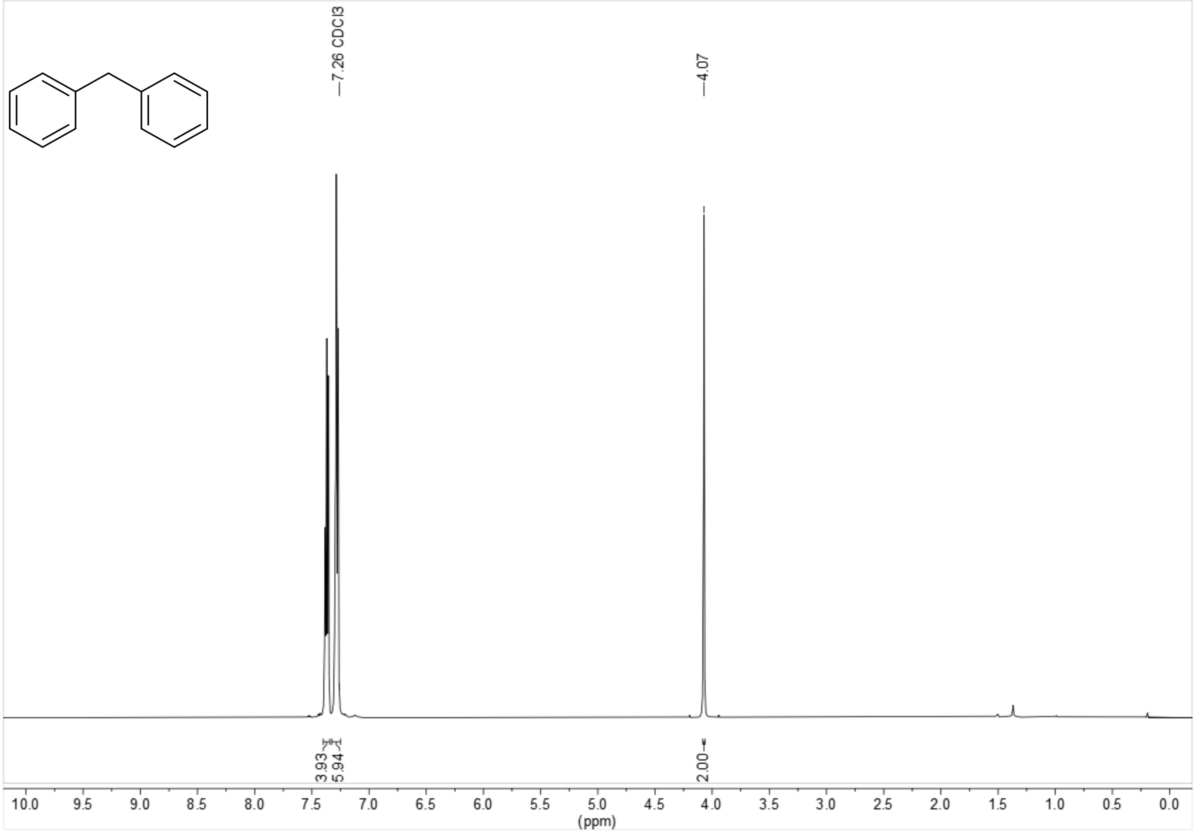

^{13}C-NMR spectrum in $CDCl_3$ at 125 MHz and rt.

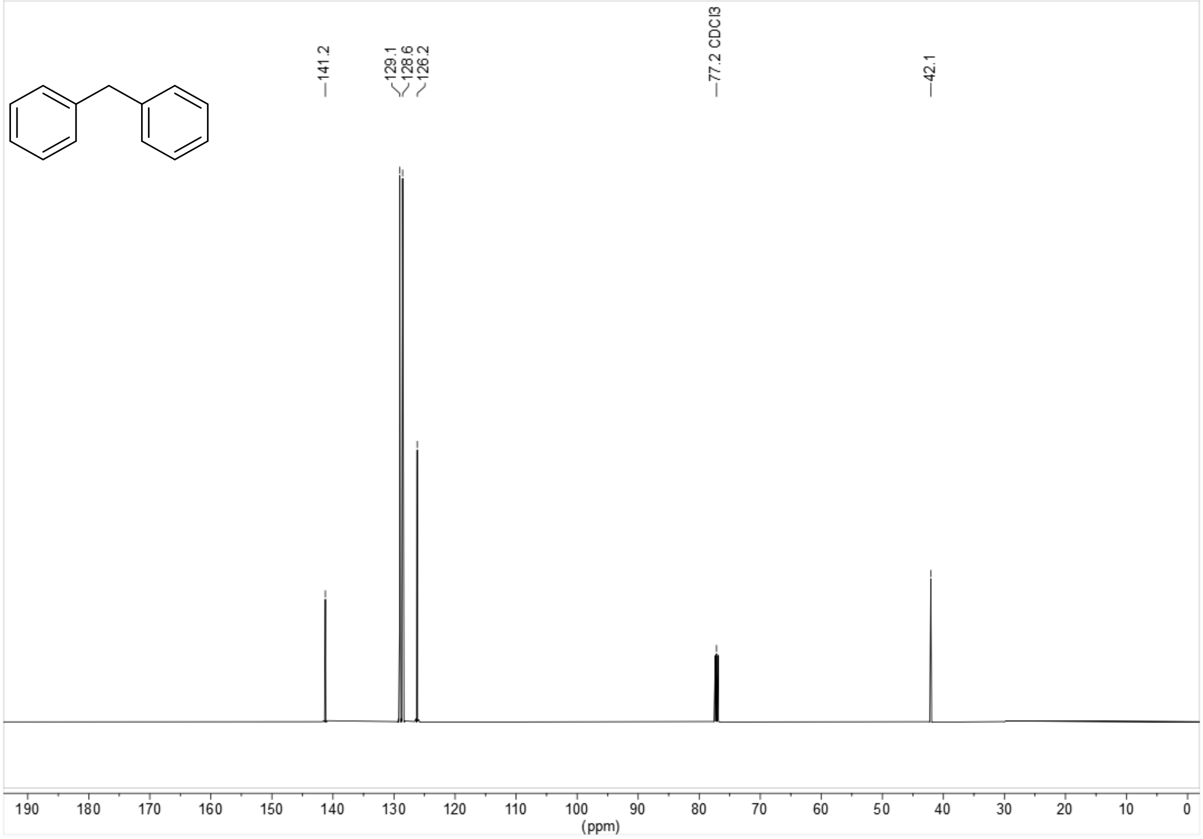

4,4'-Dimethyldiphenylmethane (4b)

^{1}H-NMR spectrum in $CDCl_3$ at 500 MHz and rt.

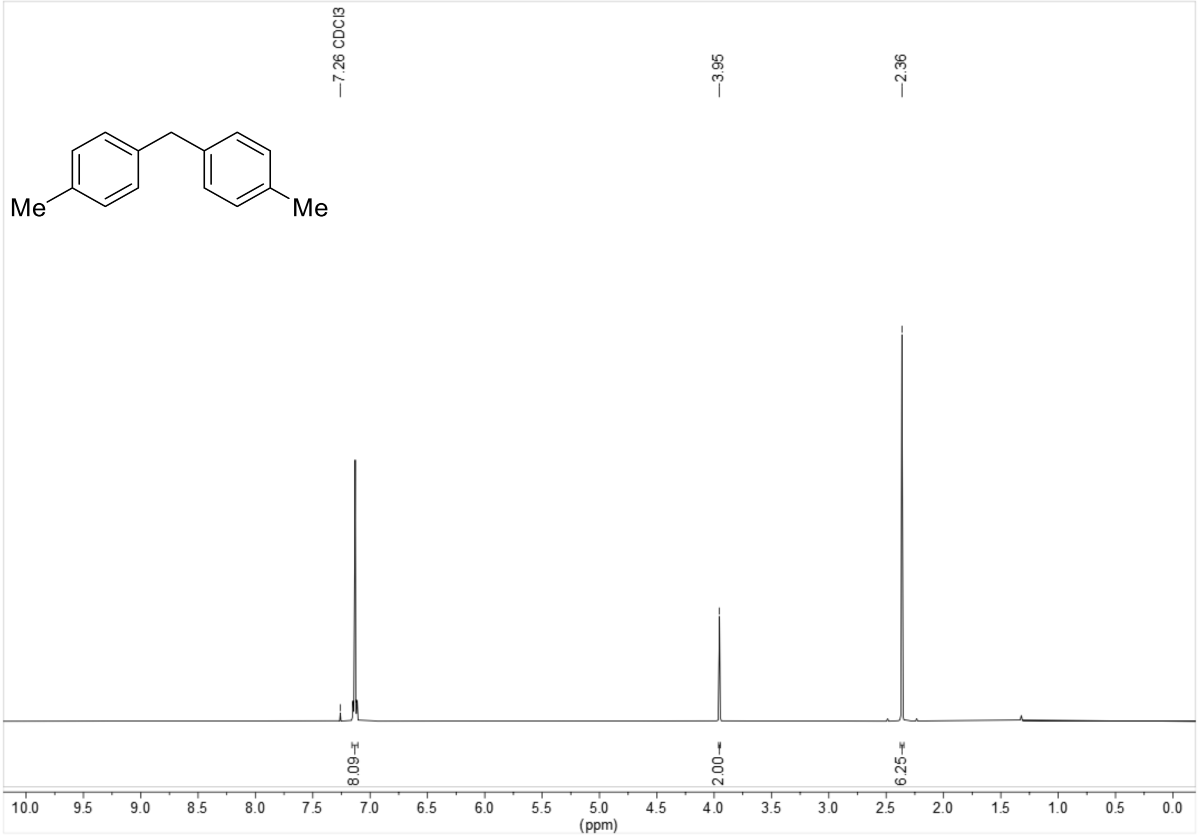

^{13}C-NMR spectrum in $CDCl_3$ at 125 MHz and rt.

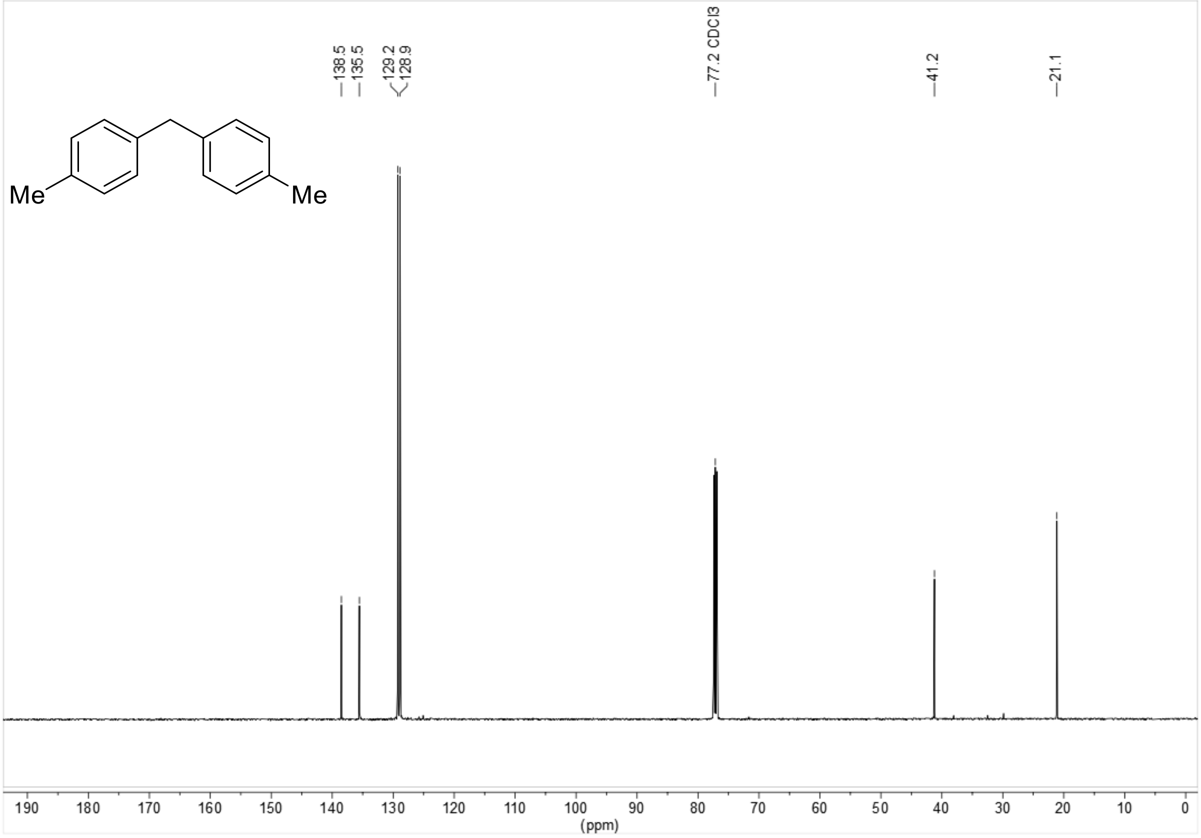

2-Benzyltoluene (4c)

^{1}H-NMR spectrum in $CDCl_3$ at 500 MHz and rt.

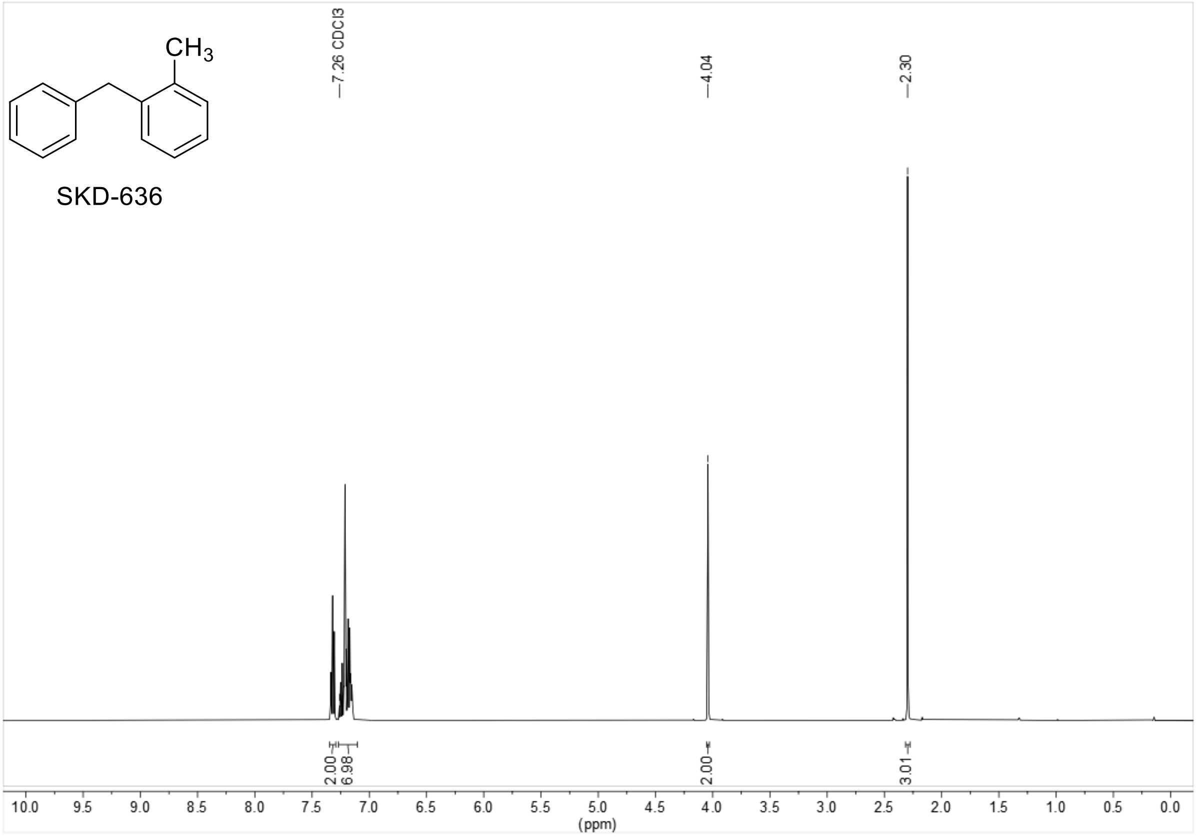

^{13}C-NMR spectrum in $CDCl_3$ at 125 MHz and rt.

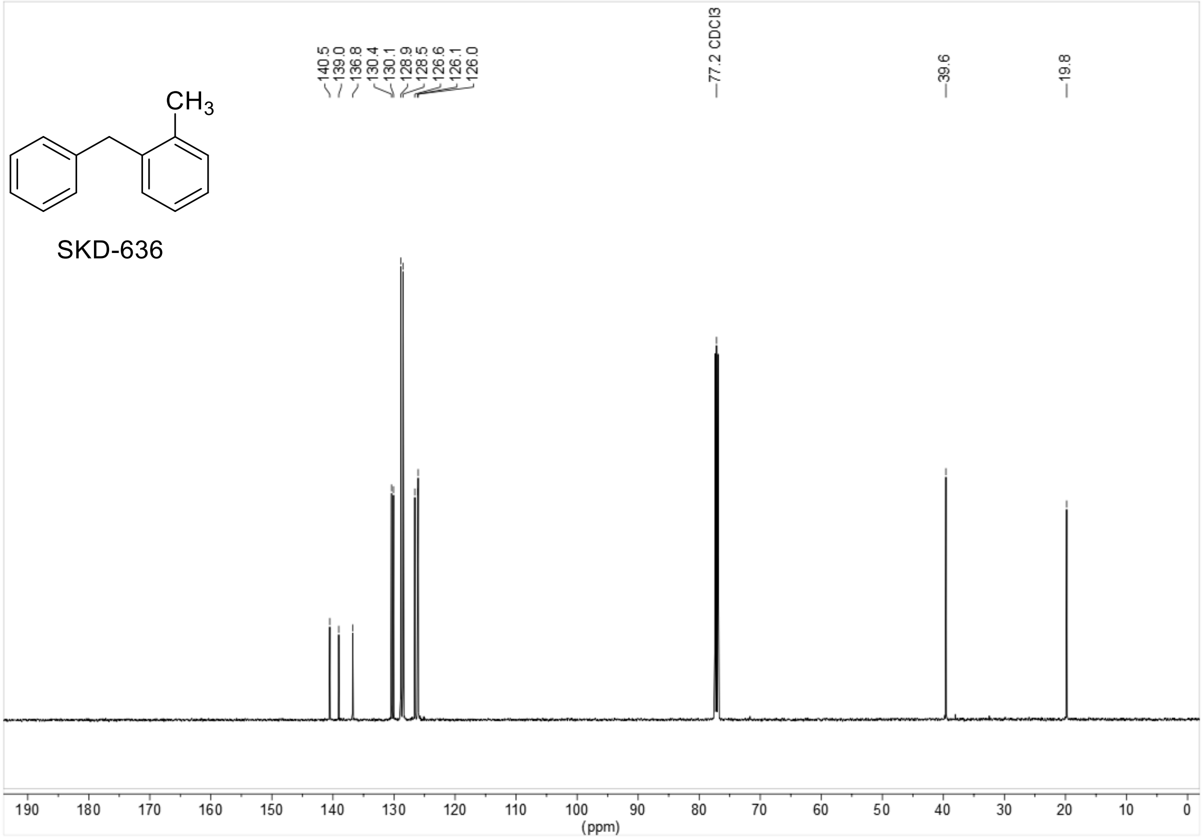

4,4’-Dianisylmethane (4e)

^{1}H-NMR spectrum in $CDCl_3$ at 500 MHz and rt.

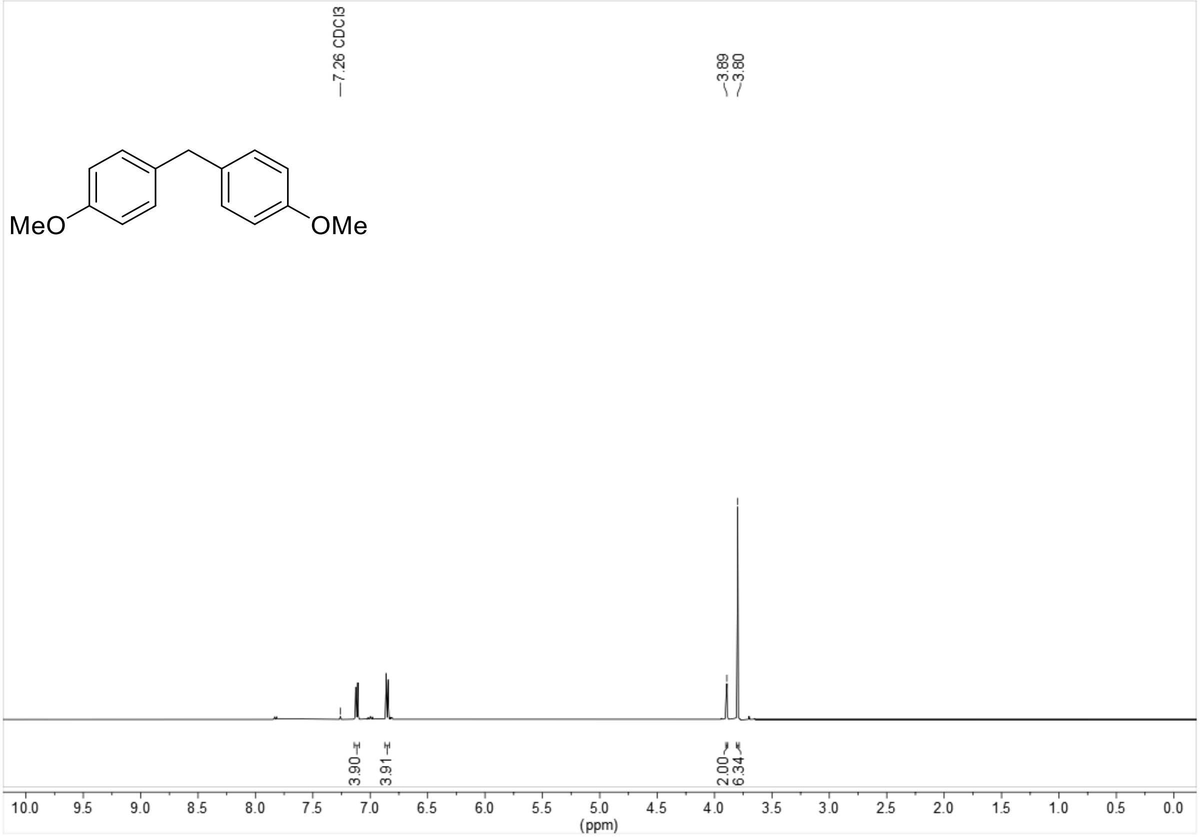

^{13}C-NMR spectrum in $CDCl_3$ at 125 MHz and rt.

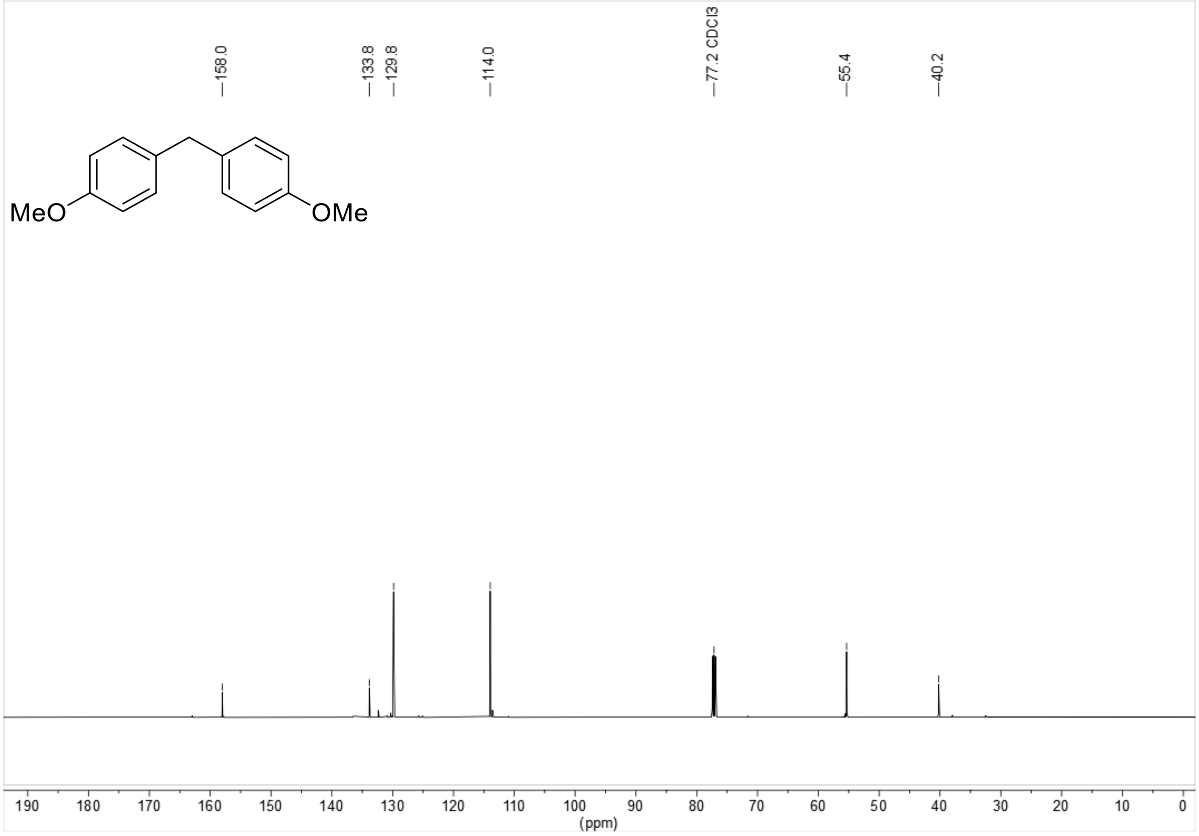

4,4'-Difluorodiphenylmethane (4f)

^{1}H-NMR spectrum in $CDCl_3$ at 500 MHz and rt.

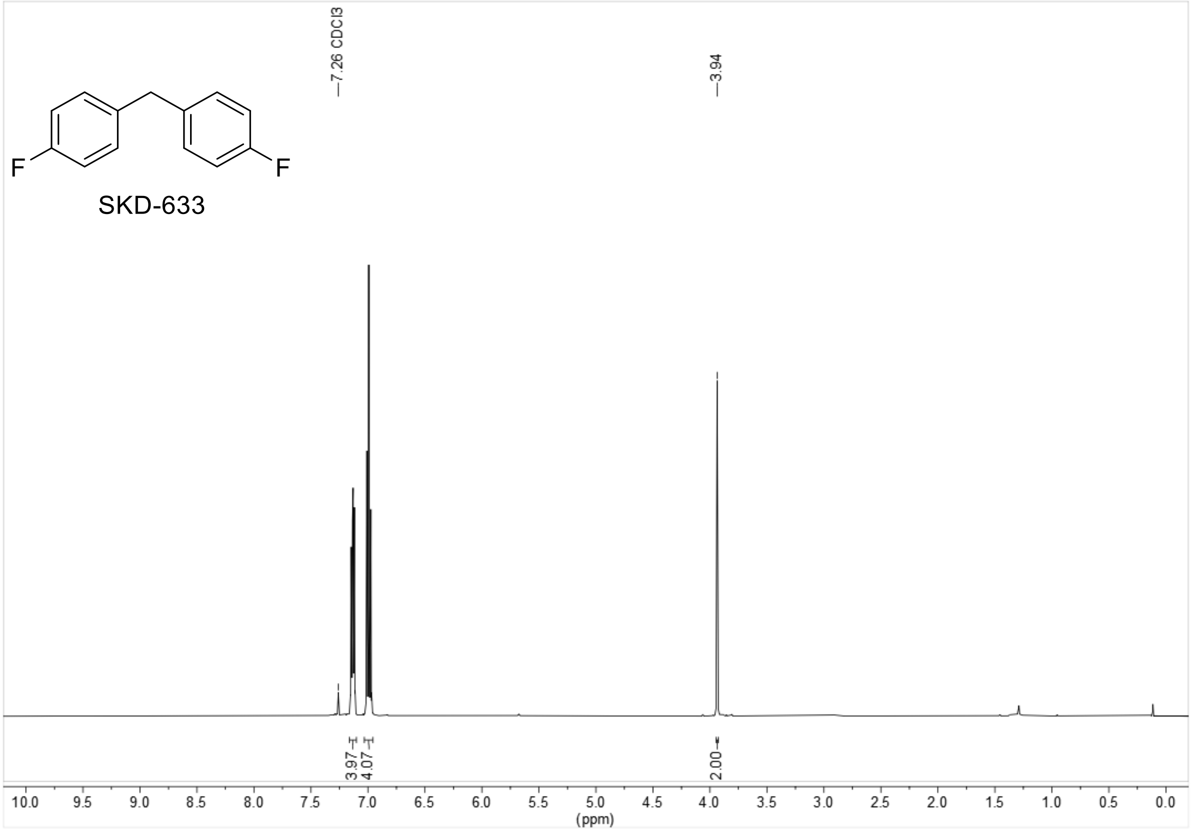

^{13}C-NMR spectrum in $CDCl_3$ at 125 MHz and rt.

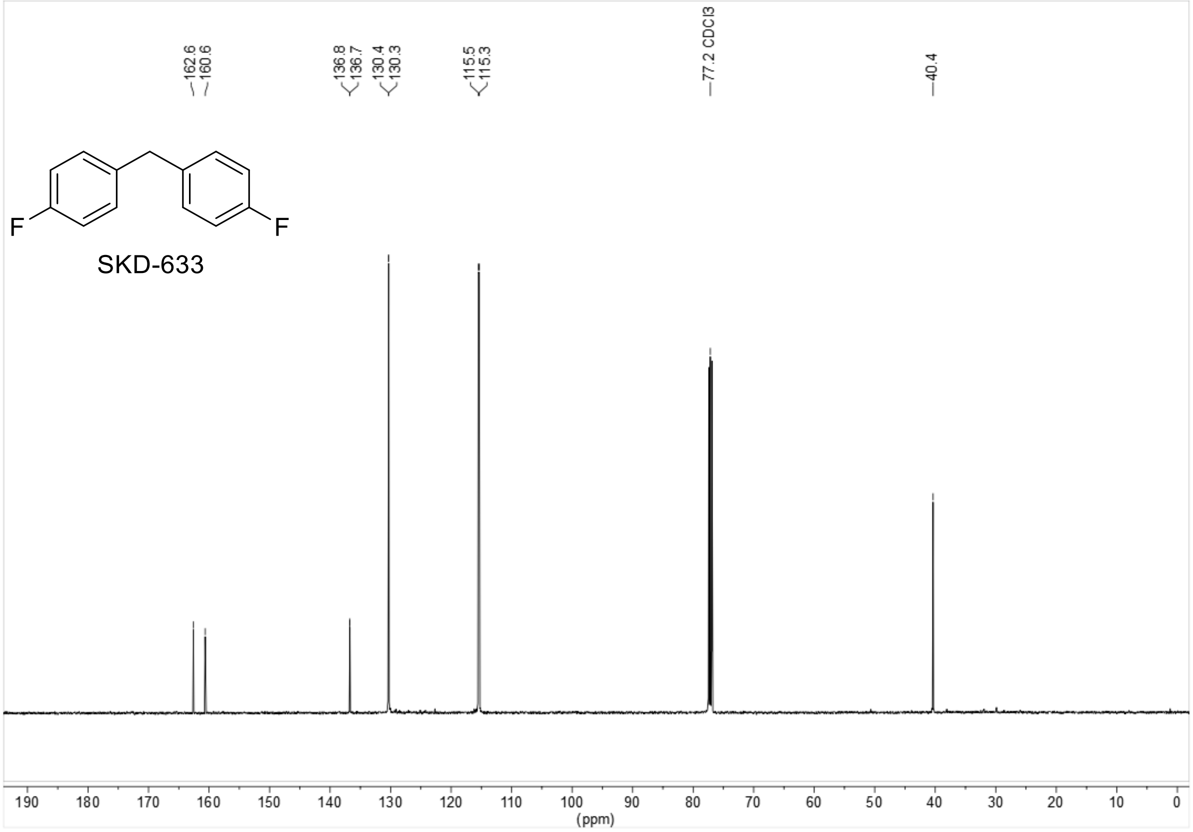

^{19}F-NMR spectrum in $CDCl_3$ at 470 MHz and rt.

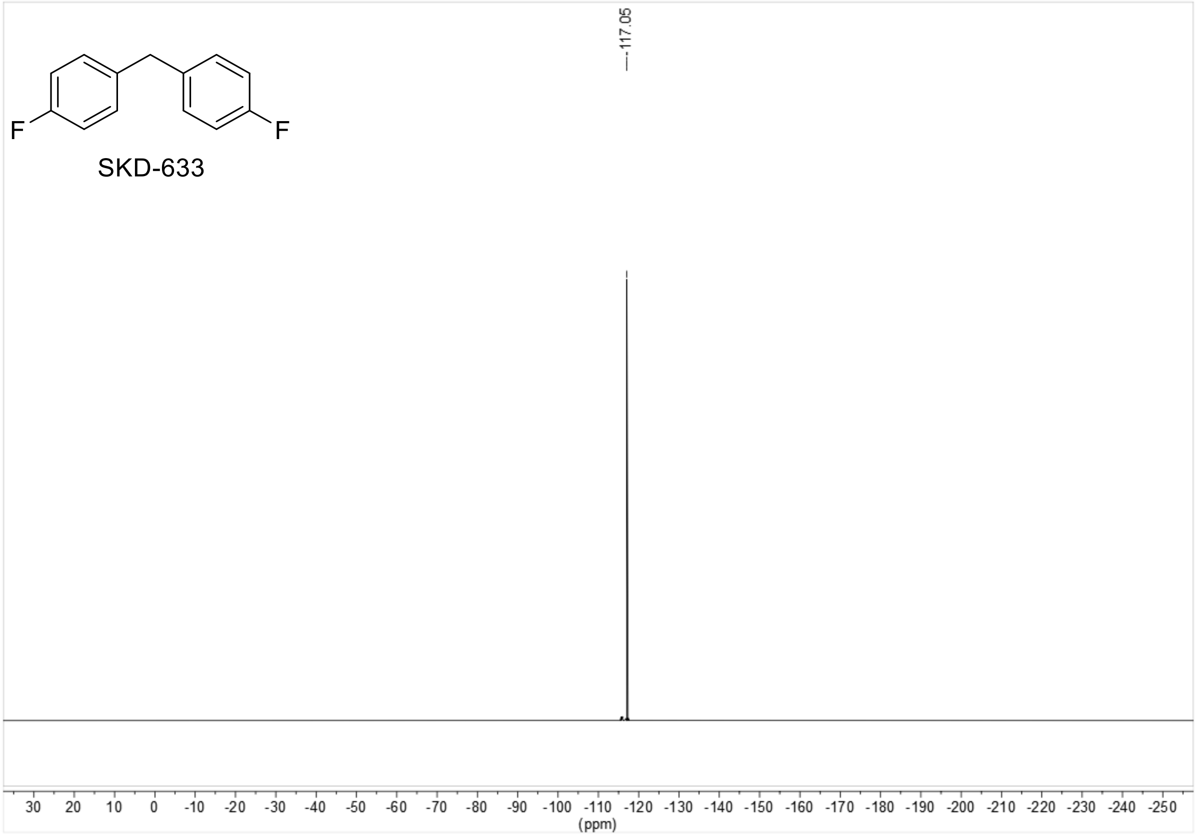

4-Chlorophenyl(phenyl)methane (4g)

^{1}H-NMR spectrum in $CDCl_3$ at 500 MHz and rt.

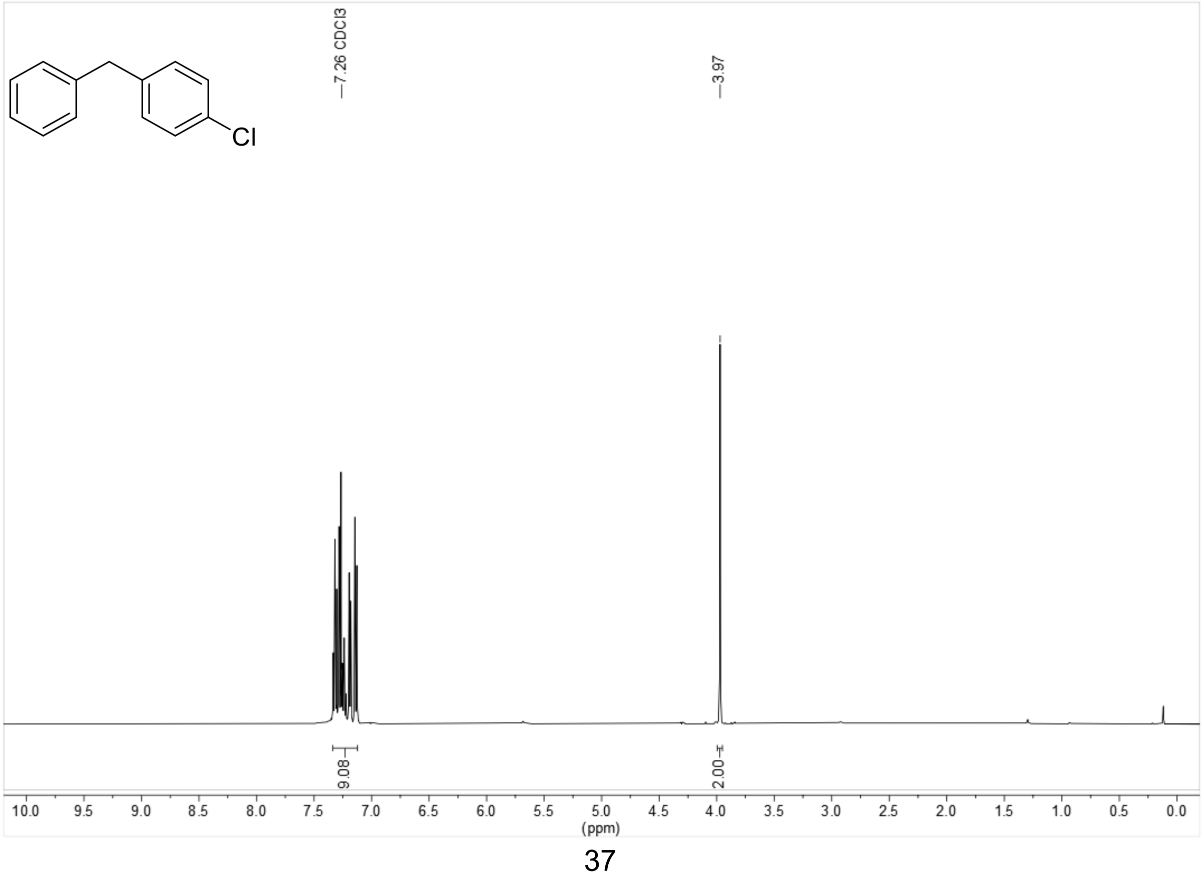

^{13}C-NMR spectrum in $CDCl_3$ at 125 MHz and rt.

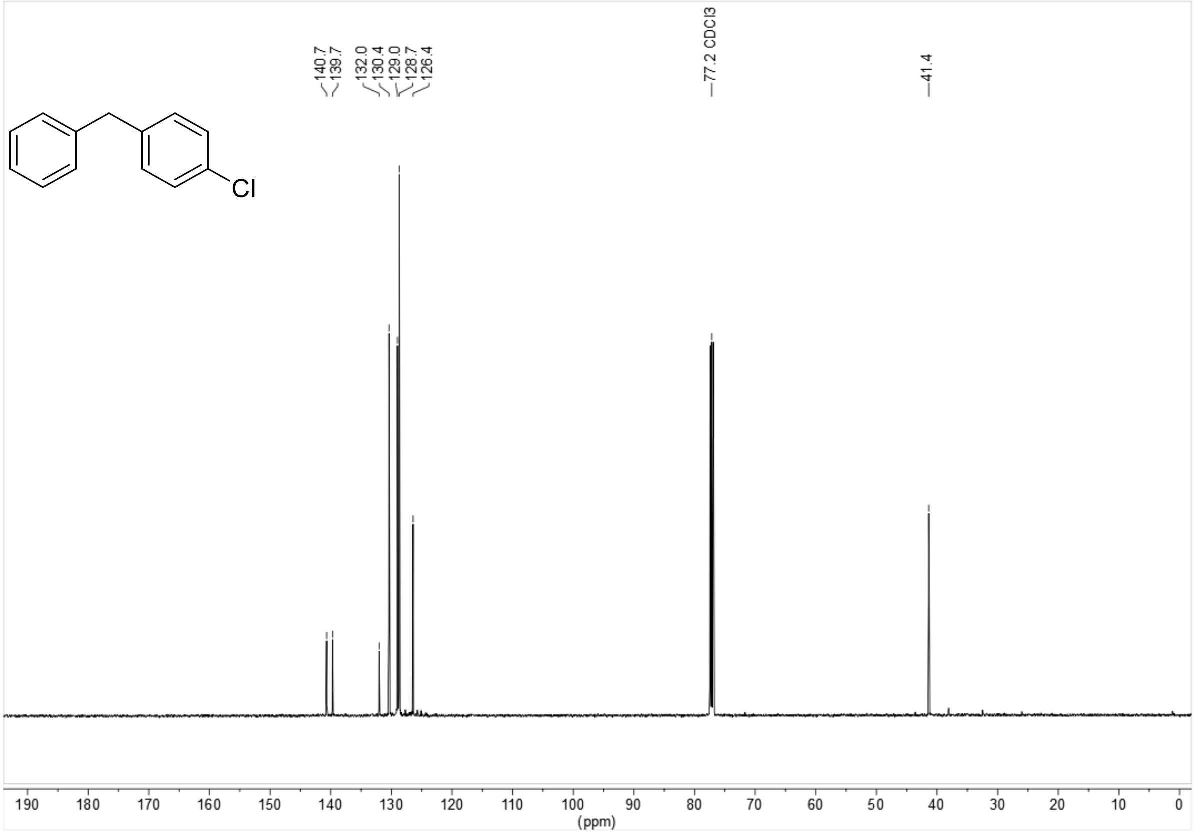

1-Benzyl-4-bromo-benzene (4h)

^{1}H-NMR spectrum in $CDCl_3$ at 500 MHz and rt.

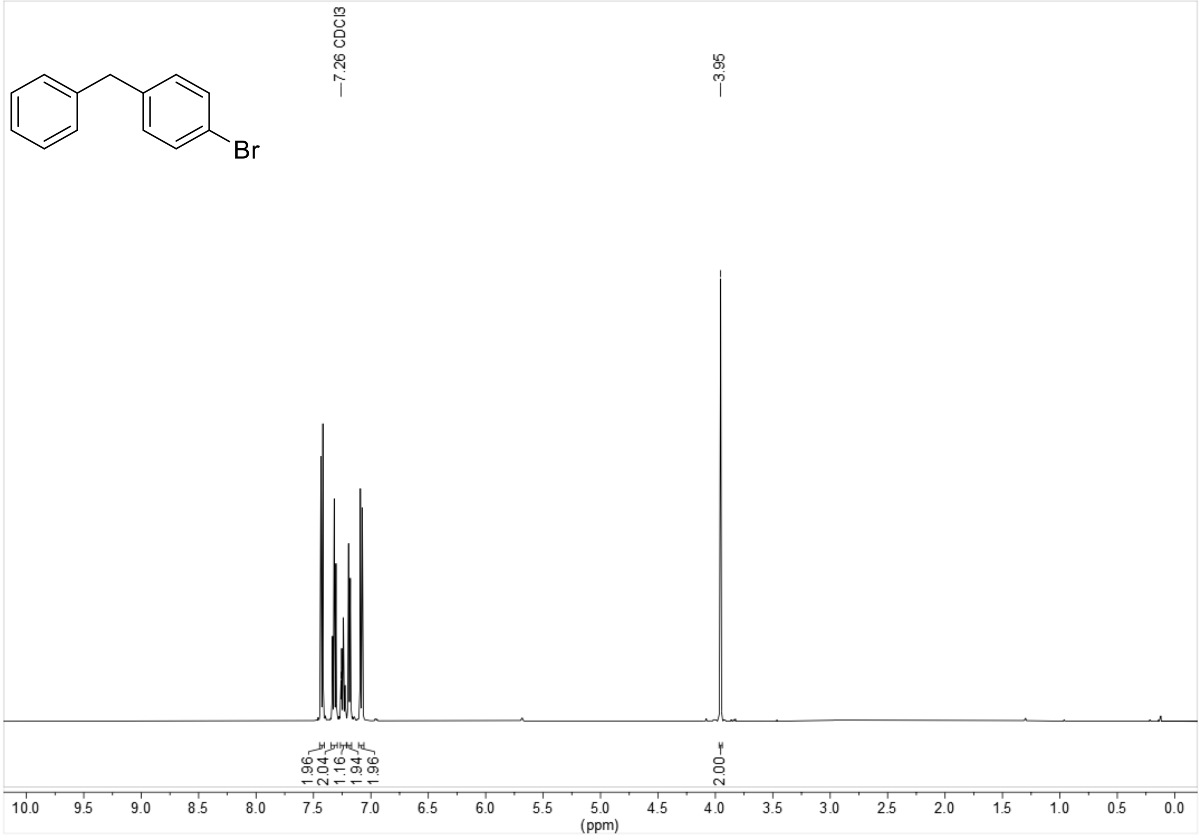

^{13}C-NMR spectrum in $CDCl_3$ at 125 MHz and rt.

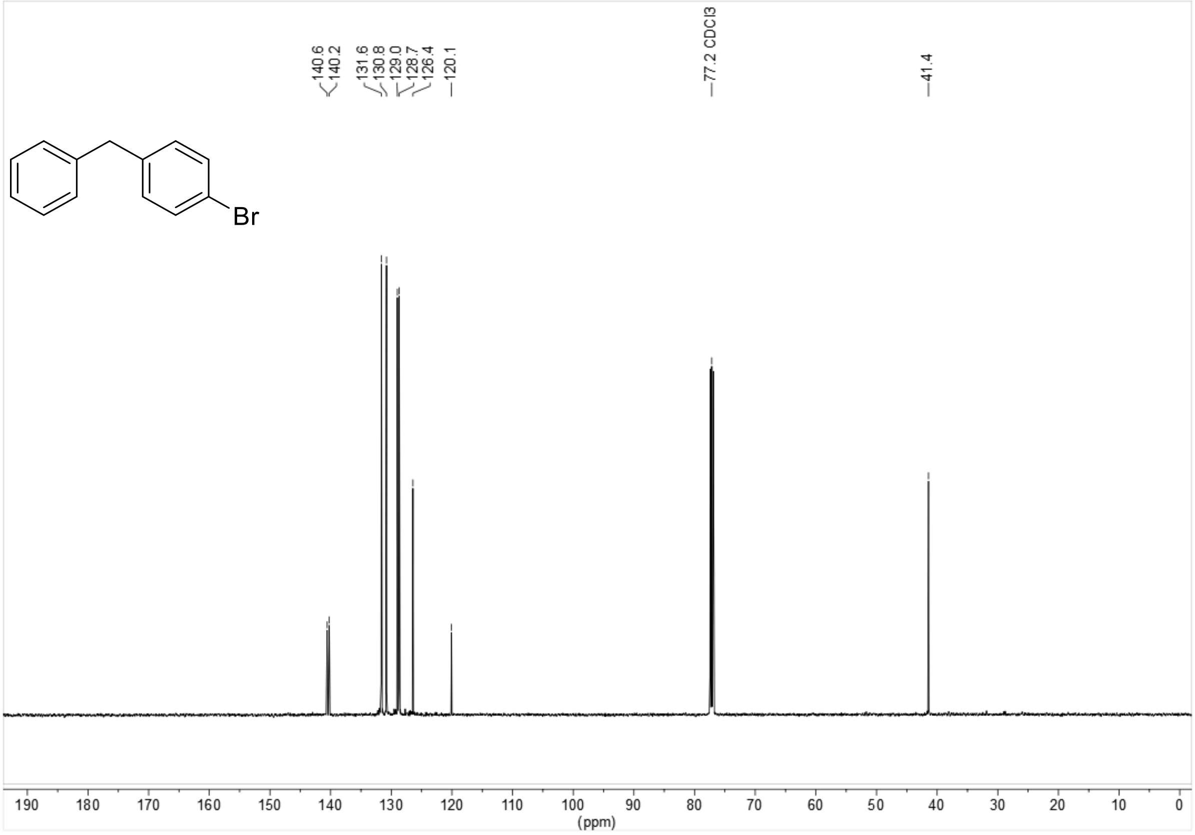

1,2-Diphenylpropane (4j)

^{1}H-NMR spectrum in $CDCl_3$ at 500 MHz and rt.

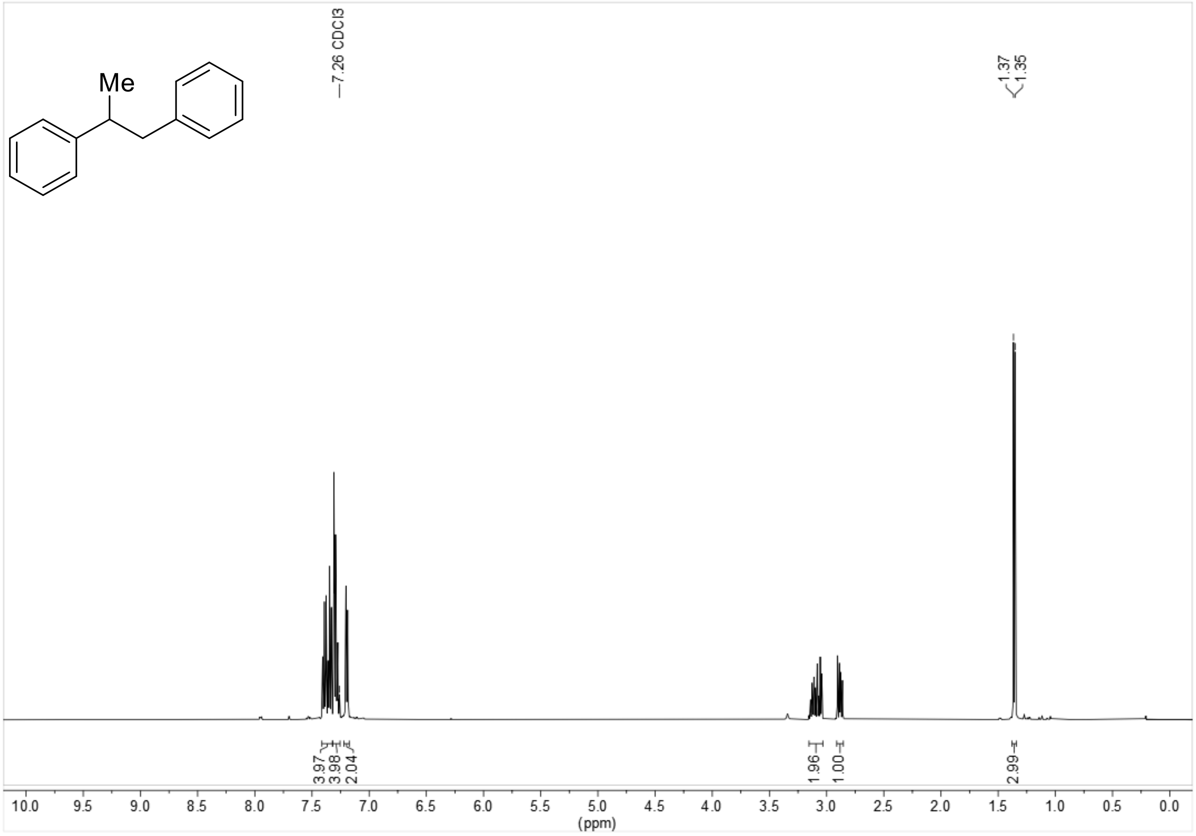

^{13}C-NMR spectrum in $CDCl_3$ at 125 MHz and rt.

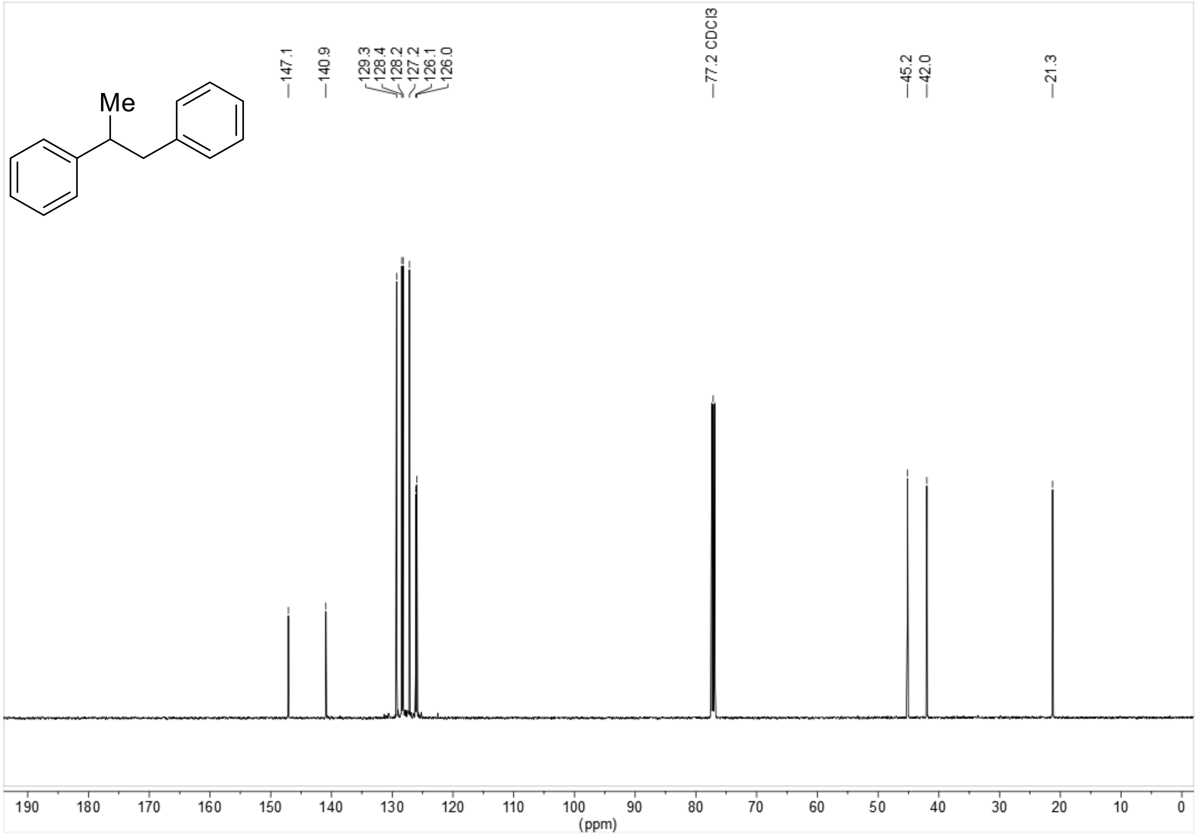

2-Ethyladamantane (4k)

^{1}H-NMR spectrum in $CDCl_3$ at 500 MHz and rt.

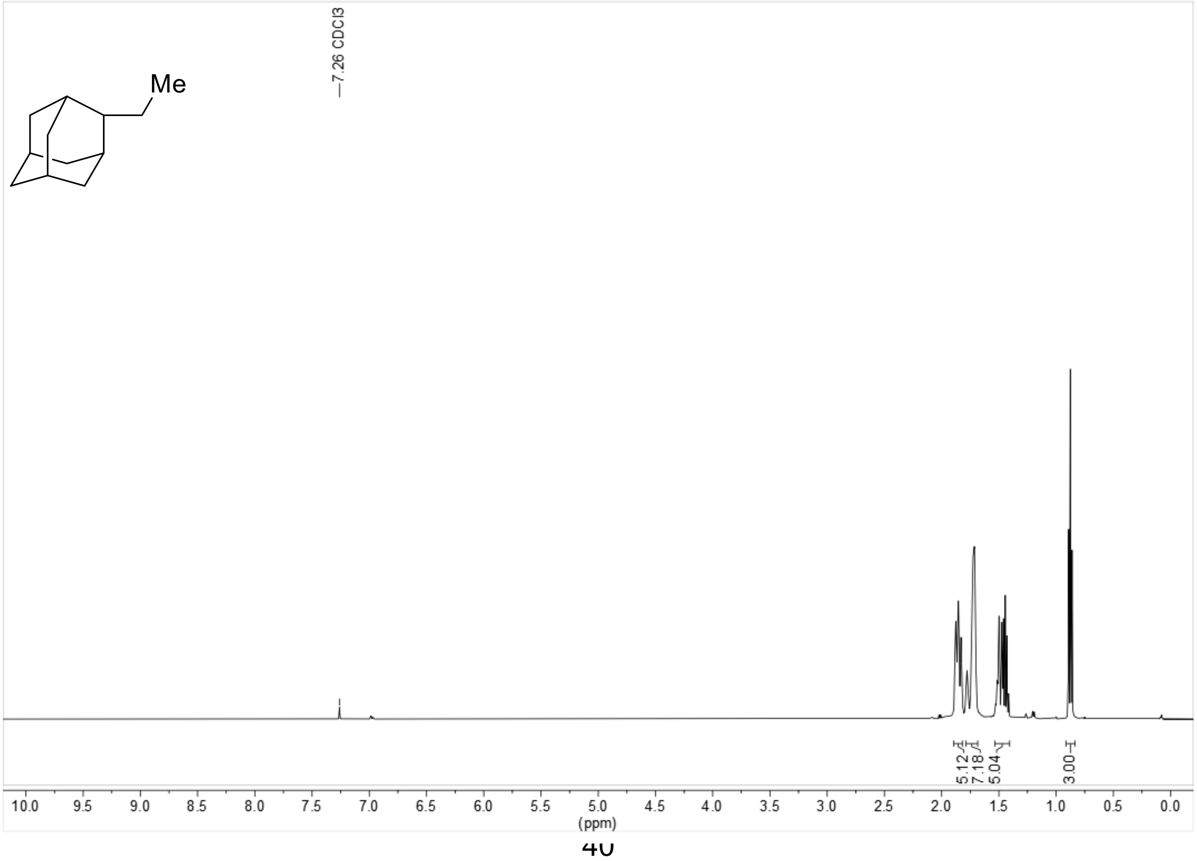

^{13}C-NMR spectrum in $CDCl_3$ at 125 MHz and rt.

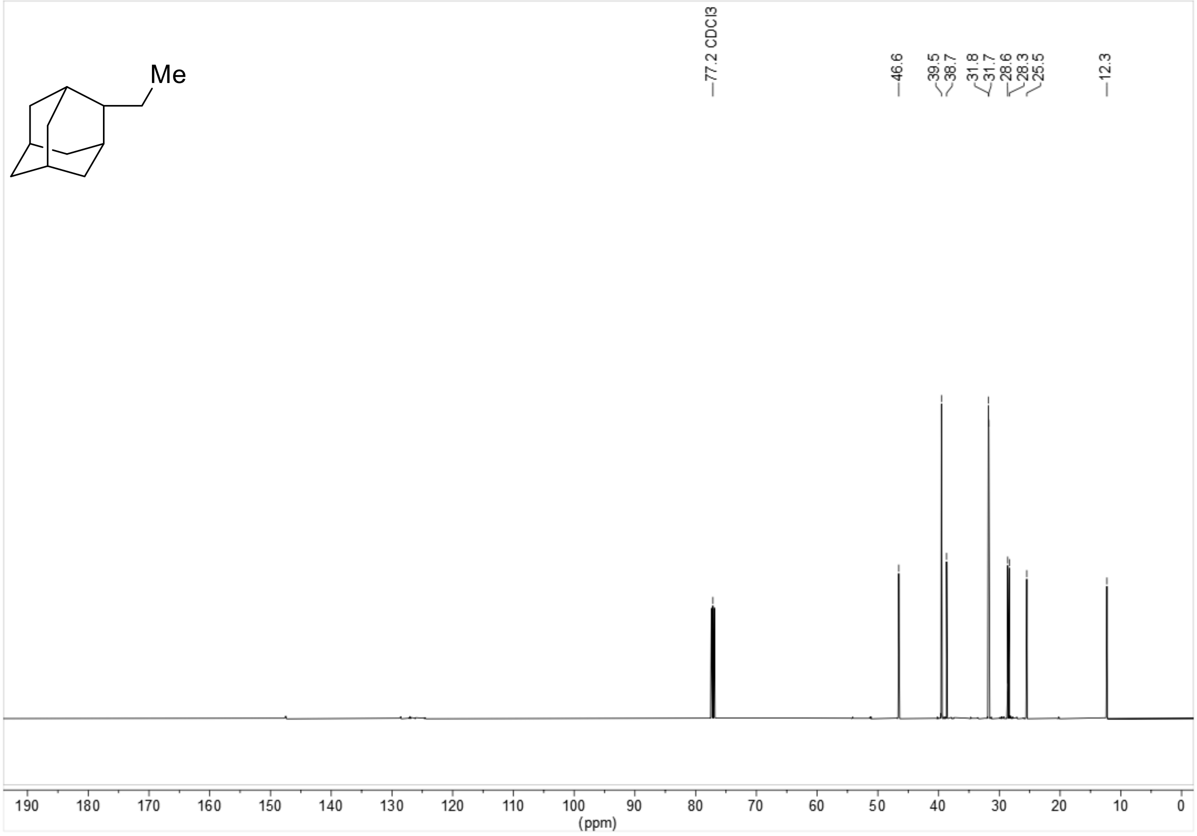

5. References

[1] S. Kail, G. Hilt, *Synlett* **2024**, *35*, 1011-1014.

[2] Y. Duan, W. Zhong, Z. Zeng, J. Feng, J. Xu, F. Yang, J. Liu, *Chem. Commun.* **2023**, *60*, 75-78.

[3] L. Li, G. Hilt, *Org. Lett.* **2020**, *22*, 1628-1632.

[4] Y. Bao, S. Ma, J. Zhu, Z. Dai, Q. Zhou, X. Yang, Q. Zhou, F. Yang, *Green Chem.* **2024**, *26*, 1356-1362.

[5] I. Chatterjee, M. Oestreich, *Org. Lett.* **2016**, *18*, 2463-2466.

[6] X. Li, K. Wang, Y. G. Li, Q. Zhao, Y. N. Ma, X. Chen, *J. Am. Chem. Soc.* **2025**, *147*, 1893-1902.

[7] S. Tu, L. Ling, R. Wei, M. Luo, X. Zeng, *Org. Lett.* **2025**, *27*, 5709-5714.

[8] J. Zhu, M. Perez, C. B. Caputo, D. W. Stephan, *Angew. Chem. Int Ed.* **2016**, *55*, 1417-1421.

[9] M. Hajek, L. Vodicka, Z. Ksandr, S. Landa, *Tetrahedron Lett.* **1972**, *40*, 4103-4106.
